# *Pitx* controls amphioxus asymmetric morphogenesis by promoting left-side development and repressing right-side formation

**DOI:** 10.1186/s12915-021-01095-0

**Published:** 2021-08-20

**Authors:** Chaofan Xing, Rongrong Pan, Guangwei Hu, Xian Liu, Yiquan Wang, Guang Li

**Affiliations:** 1 State Key Laboratory of Cellular Stress Biology, School of Life Sciences, Xiamen University, Xiangan District, Xiamen, 361102 Fujian China; 2 Jiangsu Key Laboratory of Marine Biotechnology, School of Marine Science and Fisheries, Jiangsu Ocean University, Lianyungang, 222005 China

## Abstract

**Background:**

Left-right (LR) asymmetry is an essential feature of bilateral animals. Studies in vertebrates show that LR asymmetry formation comprises three major steps: symmetry breaking, asymmetric gene expression, and LR morphogenesis. Although much progress has been made in the first two events, mechanisms underlying asymmetric morphogenesis remain largely unknown due to the complex developmental processes deployed by vertebrate organs.

**Results:**

We here addressed this question by studying *Pitx* gene function in the basal chordate amphioxus whose asymmetric organogenesis, unlike that in vertebrates, occurs essentially in situ and does not rely on cell migration. *Pitx* null mutation in amphioxus causes loss of all left-sided organs and incomplete ectopic formation of all right-sided organs on the left side, whereas *Pitx* partial loss-of-function leads to milder phenotypes with only some LR organs lost or ectopically formed. At the N1 to N3 stages, *Pitx* expression is gradually expanded from the dorsal anterior domain to surrounding regions. This leads to activation of genes like *Lhx3* and/or *Prop1* and *Pit*, which are essential for left-side organs, and downregulation of genes like *Hex* and/or *Nkx2.1* and *FoxE4*, which are required for right-side organs to form ectopically on the left side. In *Pitx* mutants, the left-side expressed genes are not activated, while the right-side genes fail to decrease expression on the left side. In contrast, in embryos overexpressing *Pitx* genes, the left-side genes are induced ectopically on the right side, and the right-side genes are inhibited. Several *Pitx* binding sites are identified in the upstream sequences of the left-side and right-side genes which are essential for activation of the former and repression of the latter by *Pitx*.

**Conclusions:**

Our results demonstrate that (1) *Pitx* is a major (although not the only) determinant of asymmetric morphogenesis in amphioxus, (2) the development of different LR organs have distinct requirements for *Pitx* activity, and (3) *Pitx* controls amphioxus LR morphogenesis probably through inducing left-side organs and inhibiting right-side organs directly. These findings show much more dependence of LR organogenesis on *Pitx* in amphioxus than in vertebrates. They also provide insight into the molecular developmental mechanism of some vertebrate LR organs like the lungs and atria, since they show a right-isomerism phenotype in *Pitx2* knockout mice like right-sided organs in *Pitx* mutant amphioxus. Our results also explain why some organs like the adenohypophysis are asymmetrically located in amphioxus but symmetrically positioned in vertebrates.

**Supplementary Information:**

The online version contains supplementary material available at 10.1186/s12915-021-01095-0.

## Background

While vertebrates exhibit external left-right (LR) symmetry, their visceral organs are LR asymmetric in terms of shape, size, position, and rotation direction [1]. For example, the human heart, spleen, and stomach are offset to the left while the gall bladder and liver sit to the right, and the left and right lung and kidney are of different size and shape [2]. Establishment of LR asymmetry is highly conserved among vertebrates and comprises three major steps: symmetry breaking, asymmetric gene expression, and LR morphogenesis [1, 3]. Symmetry breaking in most vertebrate groups is initiated by a cilia-driven fluid flow present in the LR organizer of early somite stage embryos [4]. Influenced by this flow, the *Dand5* gene first exhibits an asymmetric expression pattern (R > L) through unilateral mRNA decay in cells around the LR organizer. The Dand5 protein is a Nodal signaling inhibitor, thus its right side-biased expression leads to R < L asymmetric Nodal signaling at the LR organizer. The Nodal signaling pathway is transferred further to the left lateral plate mesoderm (LPM) from the organizer and induces asymmetric expression of *Nodal* itself, followed by *Lefty* and *Pitx2* [5].

Although progress has been made in elucidating the upstream patterning events, mechanisms controlling LR morphogenesis of visceral organs remain largely unknown [3]. This has been complicated by the complex developmental process of vertebrate LR organs, which involves many asymmetric cell behaviors and coordination of various organs within the cavity [3]. Among genes induced by the Nodal signaling pathway, *Pitx2* encodes a homeodomain-containing transcription factor and its asymmetric expression persists in developing visceral organs after asymmetric Nodal expression disappears [6]. Therefore, *Pitx2* was thought to play an essential role in asymmetric organogenesis. Consistently, knockout of *Pitx2* in mice or knockdown in chicken and frogs cause laterality defects in many visceral organs, including randomized positioning of several visceral organs and right isomerism of the lungs and atrium [7–10]. In mice, development of different LR organs requires distinct dosages of *Pitx2* activity [11–13]. During mouse and chicken gut development, *Pitx2* induces asymmetric cell condensation of dorsal mesentery (DM) attached to the gut by targeting the non-canonical Wnt signaling mediator *Daam2*, resulting in the tilting of the gut tube toward the left [14]. However, *Pitx2* appears to play a very limited role in zebrafish LR development, since its mutation or knockdown causes either no or very weak LR defects [15–17]. This indicates different requirements of *Pitx2* for LR development among vertebrates. While the development of most LR asymmetric organs relies on both *Nodal* and *Pitx2*, some asymmetric morphogenetic events depend on Nodal but not *Pitx2*. For example, in mice, *Nodal* deficiency randomizes directions of heart looping, axial rotation, and stomach sidedness [18], but none of these events are affected in *Pitx2* null mutants [11, 12]. This indicates genes other than *Pitx2* downstream of the Nodal signaling pathway also play certain roles in LR morphogenesis in vertebrates [19]. Moreover, a recent study suggests that heart looping in zebrafish, chick, and mouse is controlled by a BMP-Prrx1/Snail1 cascade on the right side [20, 21]. However, this suggestion has been questioned by a later study, which shows that neither single nor double mutants of *Prrx1a* and *Prrx1b* genes cause LR defects in zebrafish embryos [22]. This leaves it uncertain as to whether vertebrates have a right-side determinant signal.

Orthologues of the *Nodal* and *Pitx2* gene have been identified in several diverse groups of invertebrate bilaterians [23]. In most of these animals, the two genes show asymmetric expression along the LR axis. In addition, inhibiting the Nodal signaling pathway also abolishes *Pitx* expression and leads to defects in LR establishment [24–28]. This demonstrates a certain level of conservation for the genetic control of LR development between invertebrates and vertebrates. However, as in vertebrates, molecular mechanisms underlying LR morphogenesis in invertebrates are largely unknown. In addition, whether *Pitx* is required for LR asymmetry in invertebrates has not been functionally tested. To address these questions, we here analyzed *Pitx* gene function in the LR morphogenesis of the cephalochordate amphioxus. Amphioxus shows pronounced LR asymmetries in its pharynx at larval stages, with the mouth, preoral pit and the duct of the club-shaped gland (hereafter refer to as CSG) forming on the left side, and the gill slits, endostyle, and the glandular region of the CSG on the right side [28, 29]. The arrangement of the somites and peripheral nerves is also asymmetric in amphioxus beginning from the N3 stage. Importantly, unlike vertebrates, LR organogenesis of amphioxus occurs essentially in situ and does not rely on asymmetric cell behaviors, and knockout of genes essential for early LR patterning in amphioxus leads to simply either two-right-side (right isomerism) or two-left-side (left isomerism) phenotypes [24, 28, 30–33]. Despite these differences, the molecular mechanisms underlying the early development of LR asymmetry in amphioxus are very similar to that in vertebrates, as they also require cilia movement and *Dand5*, *Nodal*, and *Lefty* genes [24, 28, 30–32]. This, together with its position among chordates, indicates that amphioxus is a promising organism for understanding mechanisms and evolution of vertebrate LR morphogenesis.

We here show that *Pitx* is a major (although not the only) determinant in amphioxus LR morphogenesis, with its mutation causing loss of all left-side organs and ectopic formation of all right-side organs on the left side. We further demonstrate that different LR organs of amphioxus embryos have distinct requirements for *Pitx* dosage. Finally, we show that *Pitx* executes its functions in amphioxus LR morphogenesis probably through directly inducing left-side organs and inhibiting right-side structures. These results demonstrate a conserved role for *Pitx* in LR development among chordates, and also shed important insights into the molecular mechanisms underlying animal LR morphogenesis.

## Results

### *Pitx* encodes two isoforms with different expression patterns in amphioxus embryos

**Fig. 1:**
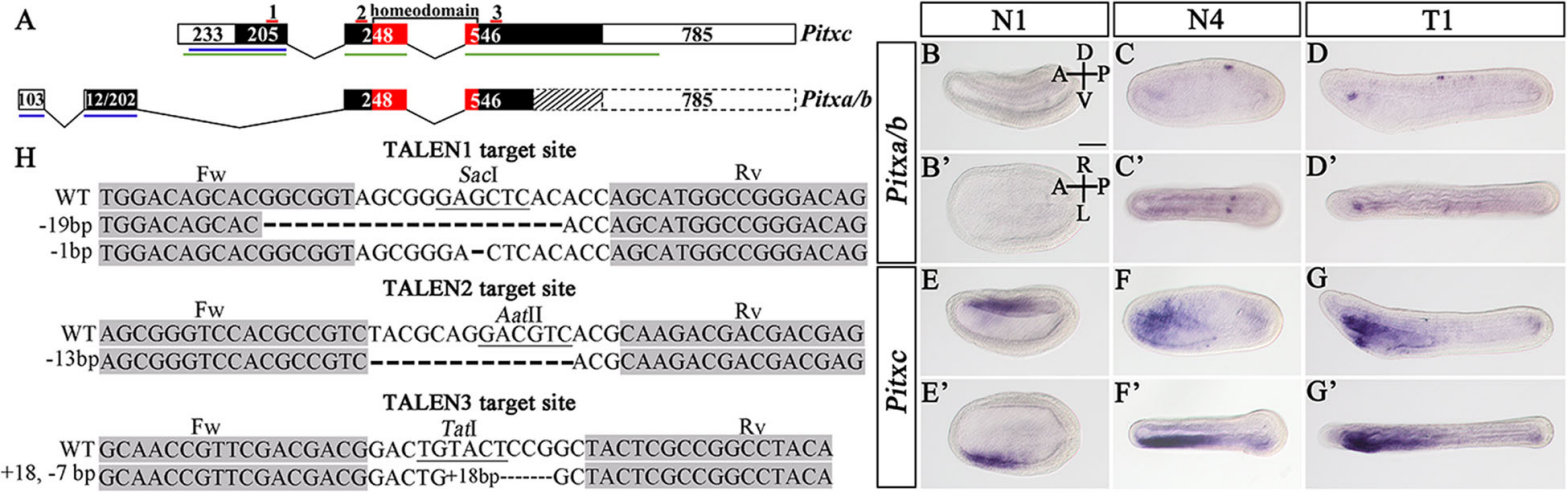
Expression patterns of amphioxus *Pitx* isoforms and TALEN target sites of *Pitx* gene. **a** Amphioxus *Pitx* isoforms. White boxes represent untranslated regions (UTR) and black and red boxes (*Pitx* homeodomain) represent coding sequences. Broken lines indicate introns. We obtained the partial sequence of *Pitxa/b* according to available amphioxus EST data and the predicted remaining sequence is supplemented as a dotted slashed box (coding sequence) and dotted white box (UTR). **b–b’** Expression of *Pitxa/b* in left lateral (**b–d)** and dorsal (**b’–d’**) views. **e–g’** Expression of *Pitxc* in left lateral (**e–g**) and dorsal (**e’–g’**) views. Blue lines in **a** show the location of *Pitxc* or *Pitxa/b* probes and green lines in **a** show the location of *Pitx* probe used for whole-mount in situ hybridization (WISH). **h** Numbers 1, 2, and 3 in **a** show the location of TALEN target sites in *Pitxc* and *Pitxa/b*. Binding sites for TALEN pairs [forward (Fw) and reverse (Rv)] used in this study are highlighted in gray. The *Sac*I, *Aat*II, and *Tat*I restriction site in the spacer is underlined respectively. WT, wild type sequence. A, anterior; P, posterior; D, dorsal; V, ventral; L, left side; R; right side. Scale bars in (**b–g’**) are 50 μm

Vertebrates have three highly conserved *Pitx2* mRNA isoforms: *Pitx2a*, *Pitx2b*, and *Pitx2c*. Among them, *Pitx2c* is asymmetrically expressed, while the other two, transcribed from an alternative promoter, are not [34]. Two corresponding isoforms (*Pitxa/b* and *Pitxc*) have also been reported in tunicate embryos [35]. Our analysis revealed two isoforms for the amphioxus *Pitx* gene that were transcribed from different transcription initiation sites and were interrupted by two and three introns respectively (Fig. 1a). As they share similar exon/intron structures to that of the two tunicate *Pitx* isoforms, we named them *Pitxa/b* and *Pitxc* respectively*.*

The expression patterns of *Pitxa/b* and *Pitxc* in amphioxus embryos was analyzed using in situ hybridization with probes corresponding to their specific exons (Fig. 1b–g’). *Pitxa/b* expression was first detected in two patches of cells in the neural ectoderm of embryos at N4 stage (Fig. 1c, c’). Double in situ analysis revealed that these *Pitxa/b*-positive cells were located more posteriorly than the dorsal Hesse organ expressing *Mop* (Additional file 1: Fig. S1). At T1 stage, a new *Pitxa/b* expression domain was observed in the posterior half of the preoral pit (Fig. 1d, d’). *Pitxc* expression was detected in the left dorsal side of embryos at N1 stage (Fig. 1e, e’), and at N4 and T1 stages, *Pitxc* was strongly expressed in the anterior left pharyngeal region and weakly in the left side of the tailbud (Fig. 1f–g’). These results demonstrated that *Pitx* in amphioxus also encodes different isoforms with distinct expression patterns, similar to *Pitx* in vertebrates and tunicates.

### *Pitx* mutation affects amphioxus development while *Pitxc* mutation does not

**Fig. 2:**
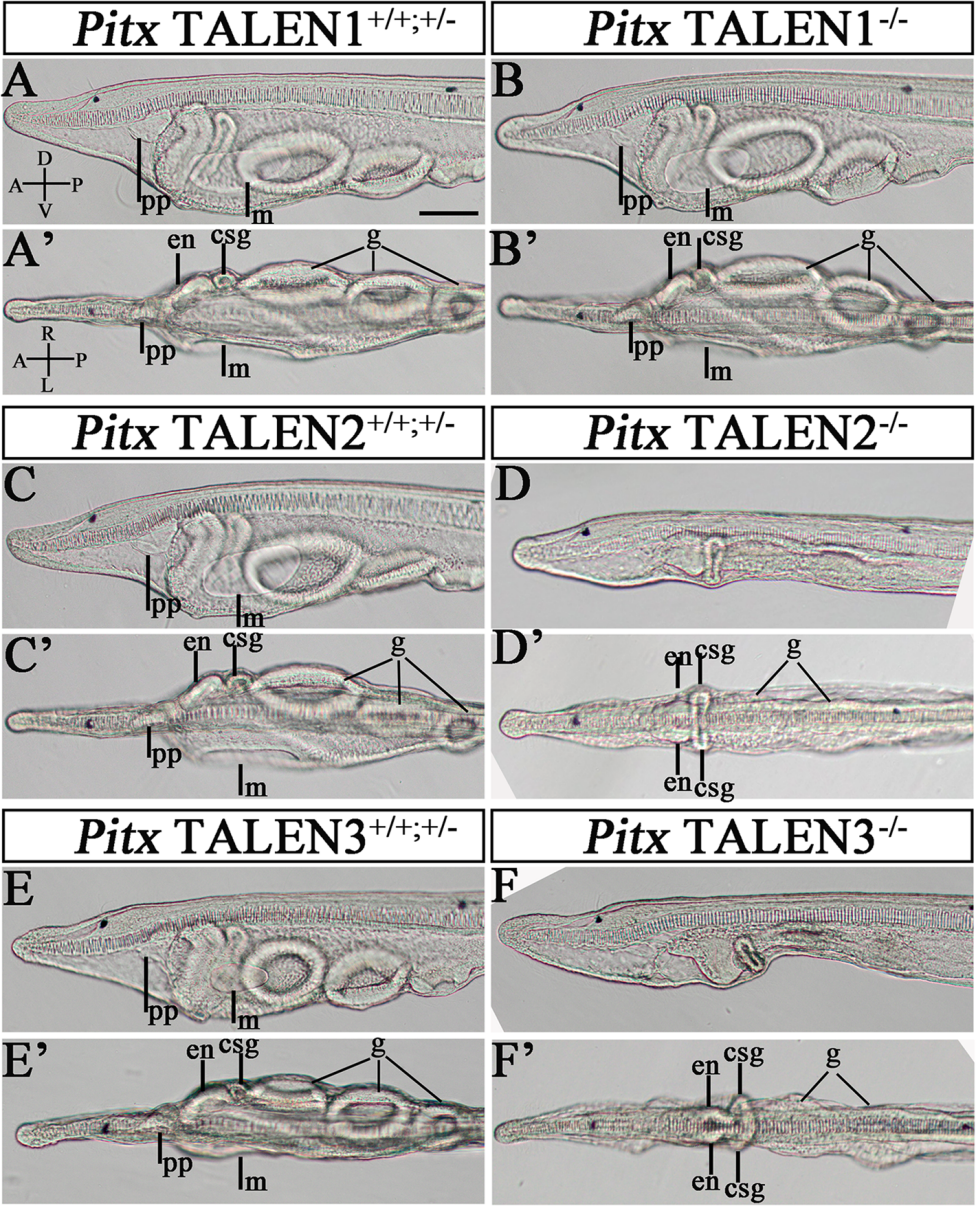
*Pitx* mutants phenotype. In *Pitx* TALEN1^+/+; +/−^ larvae (**a**, **a’**), *Pitx* TALEN2^+/+; +/−^ larvae (**c, c’**), and *Pitx* TALEN3^+/+; +/−^ larvae (**e**, **e’**), the preoral pit and mouth are formed on the left side, with the endostyle, club-shaped gland (CSG) and first gill slit developing on the right side. *Pitx* TALEN1^−/−^ mutants show normal phenotype (**b, b’**). *Pitx* TALEN2^−/−^ mutants (**d**, **d’**) and *Pitx* TALEN3^−*/*−^ mutants (**f**, **f’**) lack a mouth opening and preoral pit on the left side, forming an endostyle and CSG on each side. The larvae at L3 (3 gill slits larva) stage are observed in lateral views in (**a**, **b**, **c**, **d**, **e**, **f**) and dorsal views in (**a’**, **b’**, **c’**, **e’**, **f’**). m, mouth; pp: preoral pit; en, endostyle; csg, club-shaped gland; g: gill slit. A, anterior; P, posterior; D, dorsal; V, ventral; L, left side; R; right side. Scale bars:50 μm

To study the function of *Pitx* in amphioxus development, we generated *Pitx* mutants using the TALEN-mediated genome editing method [36, 37]. Three pairs of TALEN constructs (TALEN1-3) were assembled, which targeted the first coding exon of *Pitxc* isoform and two other coding exons shared by *Pitxc* and *Pitxa/b* (Fig. 1h). Injection of TALEN mRNAs into amphioxus embryos revealed efficient mutation (30–50%) at each of the target sites (Additional file 1: Fig. S2). Using these TALENs, we obtained four *Pitx* mutant lines (19-bp and 1-bp deletion for TALEN1, 13-bp deletion for TALEN2, and 18-bp insertion plus 7-bp deletion for TALEN3) (Fig. 1h). We further crossed these heterozygous animals (for TALEN1, females carrying the 19-bp deletion and males carrying the 1-bp deletion were used) to examine if the mutations affected embryonic development. Unexpectedly, we observed no LR or other defects in embryos derived from crosses between animals carrying mutations at the TALEN1 site (Fig. 2a–b’). Genotyping of 12 randomly selected 3-gill slit larvae (L3 stage) from the cross-identified 4 homozygous and 8 heterozygous/WT (wild type) individuals (Additional file 1: Fig. S3). The homozygous mutants can normally survive to adulthood. In contrast, we observed around 25% (136/542 for the TALEN2 site and 141/550 for the TALEN3 site) of embryos showing specific and identical phenotypes in the anterior pharyngeal region at L3 stage, from crosses of animals with mutations at the TALEN2 or TALEN3 site. Genotyping analysis confirmed that all individuals showing these phenotypes were homozygous mutants, while individuals showing normal morphology were either *Pitx* heterozygotes or wild type (Additional file 1: Fig. S4, S5). Homozygous *Pitx* TALEN2 or TALEN3 mutants died at L4 stage. Compared to WT/heterozygotes larvae (Fig. 2c, c’, e, e’), homozygous *Pitx* TALEN2 or TALEN3 mutants lacked a mouth opening, preoral pit, and CSG duct on the left side, and developed an endostyle and the glandular region of the CSG on each side (Fig. 2d, d’, f, f’). The gill slits, which first opened at the ventral midline and then extended up to the right side of the head in wild type larvae (Fig. 2c’, e’), were also affected in the two *Pitx* mutants: they appeared at the ventral side but failed to open and extended up to the right side (Fig. 2d’, f’). Notably, these phenotypes were very similar to that observed in amphioxus larvae lacking the Nodal signaling pathway activity [28]. Since *Pitx* TALEN2 and TALEN3 mutants showed identical phenotype, *Pitx* TALEN2 mutants were used for further analysis. To simplify wording, we refer to *Pitx* TALEN2 mutants as *Pitx* mutants and those carrying mutations at the TALEN1 site as *Pitxc* mutants hereafter.

### *Pitx* mutants lack left-side organs but form incomplete right-side organs on the left side

**Fig. 3:**
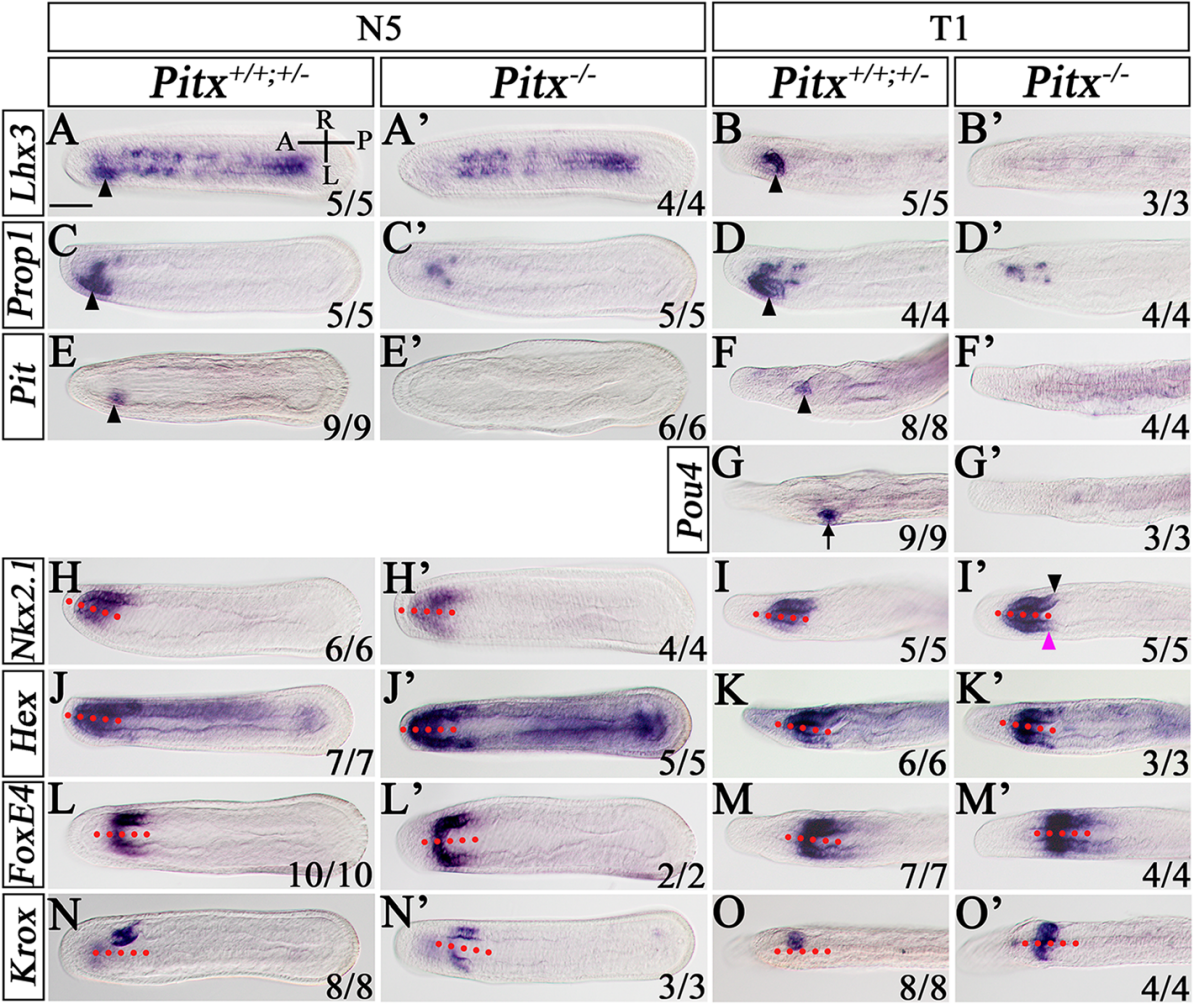
Pharyngeal organ-specific gene expression in *Pitx* TALEN2^−/−^ mutants. In situ hybridization to *Pitx* mutants at N5 and T1 stage for *Lhx3* (**a**, **a’**, **b**, **b’**), *Prop1* (**c**, **c’**, **d**, **d’**), and *Pit* (**e**, **e’**, **f**, **f’**). **g**, **g’** In situ hybridization to *Pitx* mutants at T1 stage for *Pou4*. Arrowheads indicate the preoral pit region, arrows indicate the mouth region. In situ hybridization to *Pitx* mutants at N5 and T1 stage for *Nkx2.1* (**h**, **h’**, **i**, **i’**), *Hex* (**j**, **j’**, **k**, **k’**), *FoxE4* (**l**, **l’**, **m**, **m’**), and *Krox* (**n**, **n’**, **o**, **o’**). In **i’**, the black arrowhead shows the expression boundary of *Nkx2.1* on the right side and red purple arrowhead shows expressing boundary on the left side. All images are dorsal views. Numbers in the bottom right corner of a panel show the number of times the phenotype depicted was observed, out of the total number of embryos from that genotype analyzed. A, anterior; P, posterior; L, left side; R; right side. Scale bars: 50 μm

The finding that *Pitx* mutants in amphioxus showed a symmetrical pharyngeal phenotype as observed in embryos deficient of the Nodal signaling pathway is unexpected, as *Pitx* is generally thought as a determinant of left-side development but not a LR patterning gene. To verify this, we examined expression of marker genes of pharyngeal organs including the preoral pit, mouth, CSG, and endostyle in N5 and T1 embryos. In WT and *Pitx*^+/−^ embryos, *Lhx3*, *Prop1*, and *Pit* were expressed in the left anterior endoderm where the preoral pit will later form (Fig. 3a–f, arrowheads). Consistent with loss of the preoral pit in *Pitx*^−/−^ larvae, expression of these genes in the prospective preoral pit region were lost in *Pitx*^−/−^ embryos (Fig. 3a’–f’). Likewise, *Pou4*, which is normally expressed in the developing mouth on the left side (Fig. 3g, arrow), also lost its expression in *Pitx*^−/−^ mutants (Fig. 3g’). *Nkx2.1* and *Hex* which are expressed in the endostyle are asymmetrically expressed in WT and *Pitx*^+/−^ embryos in a L < R manner (Fig. 3h–k). In *Pitx*^−/−^ mutants, *Nkx2.1* and *Hex* expression became symmetric, with the expression pattern on both sides being similar to that in the right side of wild type embryos (Fig. 3h’–k’). However, careful examination revealed that *Nkx2.1* expression on the right side (Fig. 3i’, black arrowhead) was not identical to its expression on the left side (Fig. 3i’, red purple arrowhead): the former was extended more posteriorly than the latter (Fig. 3i’). This indicated that the endostyle region was almost but not fully duplicated on the left side of *Pitx*^−/−^ mutants. A similar case was also observed in the CSG of *Pitx*^−/−^ mutants. In WT and *Pitx*^+/−^ embryos, *FoxE4* was expressed in the whole CSG including the right-sided glandular region and the left-sided duct (Fig. 3l, m), while *Krox* was transcribed only in the glandular region (Fig. 3n, o). In *Pitx*^−/−^ embryos, although *FoxE4* expression on the right side seemed to be fully duplicated on the left side (Fig. 3l’, m’), *Krox* expression was not since its expression domain on the left side was slightly smaller than that on the right side (Fig. 3n’, o’). These results demonstrated that the pharyngeal structure of *Pitx*^−/−^ mutants was not fully symmetrical unlike that observed in embryos lacking Nodal signaling pathway [28]. Consistent with this, asymmetric expression patterns of the LR patterning genes, like *Dand5*, *Nodal*, *Lefty*, and *Pitx* were not altered in *Pitx*^−/−^ embryos at N0 (Additional file 1: Fig. S6a’), N2 (Additional file 1: Fig. S6b’, c’, d’), N4 (Additional file 1: Fig. S6e’, f’, g’), or T1 stages (Additional file 1: Fig. S6h’, i’, j’), although *Pitx* expression was slightly downregulated in *Pitx*^−/−^ embryos at all examined stages (Additional file 1: Fig. S6d’, g’, j’) and *Nodal*, *Lefty*, and *Pitx* expression in the forming preoral pit was lost at T1 stage (Additional file 1: Fig. S6h’, i’, j’).

**Fig. 4:**
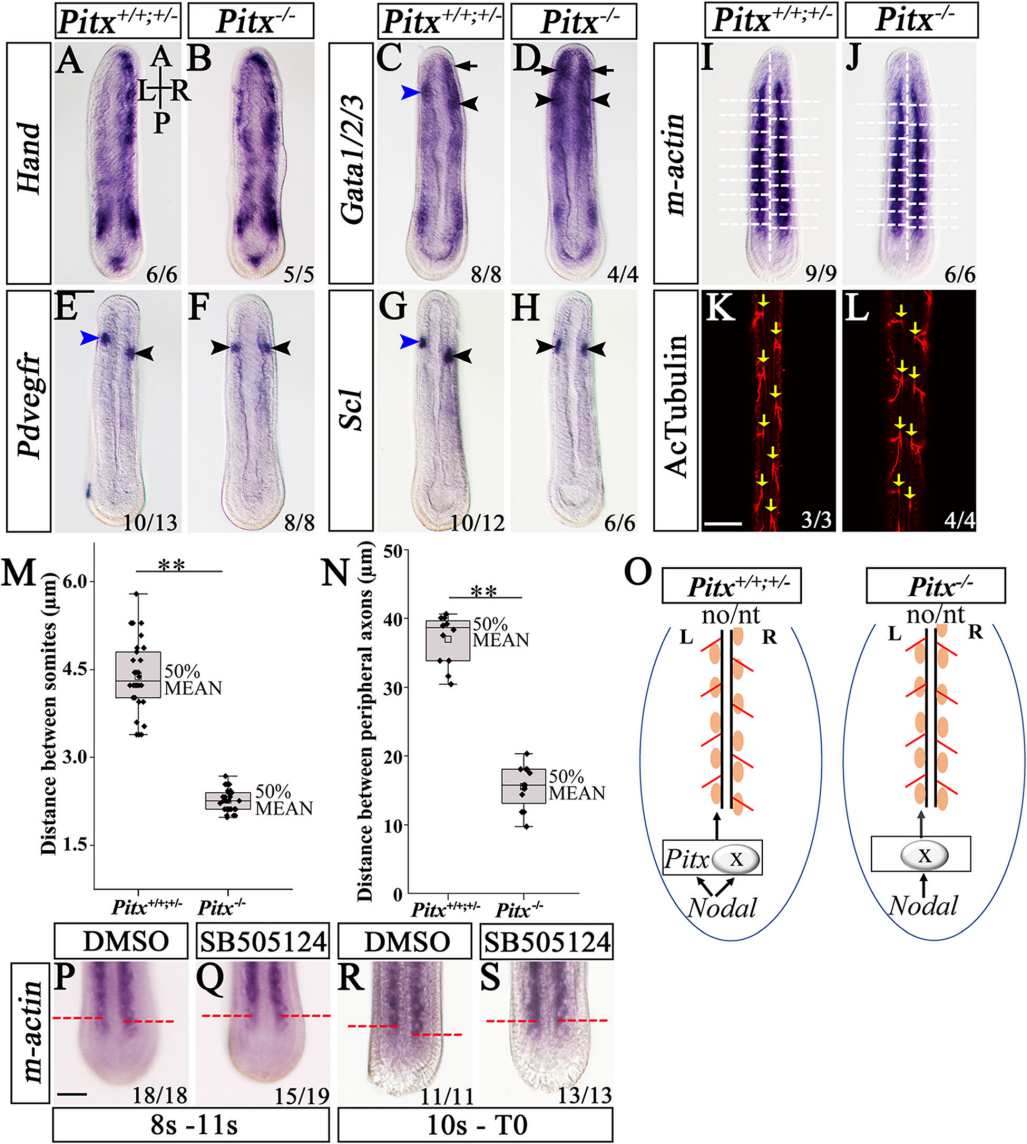
Arrangement of mesoderm-derived organs and peripheral nerves in *Pitx* mutants and pharmacological treatment. In situ hybridization to *Pitx* mutants at N5 stage for *Hand* (**a**, **b**), *Gata1/2/3* (**c**, **d**), *Pdvegfr* (**e**, **f**), and *Scl* (**g**, **h**). Blue arrowheads are Hatschek’s nephridium and black arrowheads are hematopoietic region. Black arrows are the HRD. **i**, **j** The arrangement of somites is marked by *m-actin* in N5 stage embryos. Longitudinal dashed lines indicate notochord, transversal dashed lines mark somite outlines. **k**, **l** Yellow arrows indicate peripheral nerves marked by acetylated α-tubulin. Quantification of the distance between a pair of somites (**m**), and the distance between a pair of peripheral nerves (**n**). Posterior five pairs of somites are used for measure in one embryo, and four pairs of peripheral nerves shown in the image are used for measure. Significance determined by *t*-test is indicated by asterisks: **P < 0.01. **p–s** The arrangement of posterior somites is marked by *m-actin.* Dashed lines mark outlines of newly budded somites. **o** The yellow ellipses indicate somites and red lines indicate peripheral nerves. no, notochord, nt, neural tube. All the embryos in above images are dorsal views. Numbers in the bottom right corner of a panel show the number of times the phenotype was seen, out of the total number of embryos from that genotype analyzed (**a–l**), or the phenotype was observed in the total number of embryos examined (**p–s**). A, anterior; P, posterior; L, left side; R; right side. Scale bars: 50 μm

In addition to the LR organs examined above, Hatschek’s nephridium, hematopoietic domains, and Hatschek’s right diverticulum (short for HRD) are also positioned asymmetrically on one side of amphioxus embryos. Among them, the HRD is derived from the right anterior-dorsal endoderm, and Hatschek’s nephridium and hematopoietic domains are developed from the left first and right first somites, respectively [38, 39]. To evaluate if the development or positioning of these organs were disrupted due to *Pitx* loss-of-function, we analyzed genes specifically expressed in them in *Pitx*^−/−^ mutants at N5 stage. In WT and *Pitx*^+/−^ embryos, *Gata1/2/3*, *Pdvegfr*, and *Scl* exhibited two bilateral and slightly asymmetrical expression domains, which respectively denoted left-side Hatschek’s nephridium (Fig. 4c, e, g, blue arrowhead) and the right-side hematopoietic domain (Fig. 4c, e, g, black arrowhead) [38]. In *Pitx*^−/−^ mutant embryos, however, their expression was bilaterally symmetric (Fig. 4d, f, h, black arrowhead). To determine the identity of the expression domains, we analyzed *Pax2/5/8* gene, which is expressed in Hatschek’s nephridium but not in the hematopoietic center [40]. We found that *Pax2/5/8* expression in the prospective nephridium is absent in *Pitx*^−/−^ mutants (Additional file 1: Fig. S7c, d). This result indicated that *Pitx*^−/−^ mutants lacked Hatschek’s nephridium but formed a hematopoietic domain on each side. We also noticed a *Gata1/2/3* expression domain in the right-side HRD in normal embryos (Fig. 4c, arrow) and found that this domain was present on both sides of *Pitx*^−/−^ mutants (Fig. 4d, arrow). Moreover, asymmetric expression (R > L) of the *Hand* gene in the lateral/ventral mesoderm derived from amphioxus somites [38] became bilaterally expressed in *Pitx*^−/−^ mutants although still in a R > L pattern (Fig. 4b).

Together, the above results indicate that in *Pitx* mutants, all left-side organs are lost while the right-side organs form normally on the right side and ectopically (albeit not incompletely) on the left side.

### *Pitx* acts downstream of Nodal signaling to control asymmetric arrangement of amphioxus somites and peripheral nerves

Different from vertebrates, amphioxus somites and peripheral nerves (aligned with the somites) are asymmetrically arranged along the LR axis, with the left ones being offset about half a segment anterior to the right ones [41]. These asymmetries become first recognizable at mid-neurula (with 8–10 somites) when somitogenesis shifts from a symmetrical way by enterocoelous outpocketing of paraxial mesoderm, to a left-to-right alternate fashion by pinching off from the tailbud [42]. To assay if the arrangement of the somites and peripheral nerves were affected in *Pitx*^−/−^ mutants, we examined expression of the *m-actin* gene, a marker of the somites at stage N5, and acetylated tubulin, a marker of the peripheral nerves at L3 stage [28]. We found both markers were asymmetrically expressed in the mutants, similar to that observed in WT and heterozygous embryos (Fig. 4i, k). However, compared to WT and heterozygous embryos, the mutants exhibited significantly shorter distances between the left somites or peripheral nerves and their corresponding ones on the right side (Fig. 4j, l, m, n). These results showed that the asymmetric arrangement of the somites and peripheral nerves was weakened but not abolished in *Pitx*^−/−^ mutants.

Between N5 and early larvae stages, both *Nodal* and *Pitx* are constantly expressed in the left side of the amphioxus tailbud (Additional file 1: Fig. S6e, g, h, j, arrowheads). We speculated that Nodal signaling pathway acts upstream of *Pitx* to determine somite asymmetry. To test this, we treated embryos with the Nodal signaling inhibitor SB505124 in different developmental windows and examined *Pitx* expression as well as the asymmetry of newly formed somites. To exclude the influence of Nodal function in early LR patterning on the analysis, we treated embryos with the drug after the N4 stage when the LR axis is thought to have been fixed [28, 33], namely from 8S (neurula with 8 somites) to 11S (neurula with 11 somites), and from 10S (neurula with 10 somites) to around T0. As expected, both treatments eliminated *Pitx* expression in the tailbud of the treated embryos (Additional file 1: Fig. S8a’-d’) and abolished the asymmetry of the newly budded somites (Fig. 4p–s). These results indicated that the Nodal signaling pathway regulated the asymmetric generation of somites from the tailbud, and that besides *Pitx*, other factors (X) downstream of Nodal signal also played vital roles in the process (Fig. 4o). We also conducted treatments on later developmental stages, from T0 to T1, and from T1 to L0 stage. Similarly, both treatments could effectively eliminate *Pitx* expression from the tailbud (Additional file 1: Fig. S8i’-l’). We did not assess if the treated embryos had symmetric somites, since somites at these stages of embryos were not easy to recognize.

### *Pitx* is required for amphioxus LR organ development in a dosage-sensitive manner

**Fig. 5:**
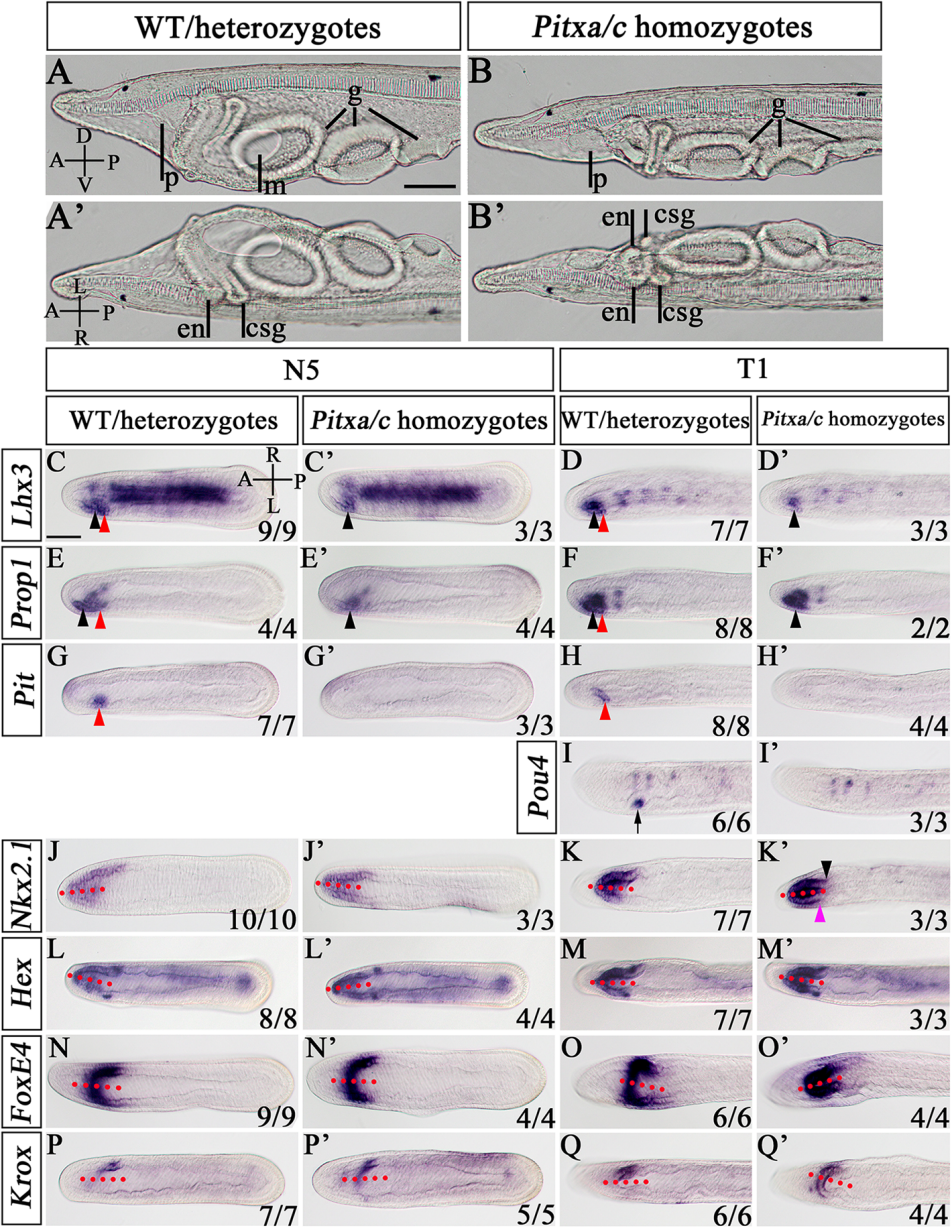
Partial loss of *Pitx* function exhibits a mild phenotype and disturbs pharyngeal organs asymmetry. **a, b** Left lateral view of the L3 larvae (3-gill slits). **a’, b’** The larvae are shown in a view between right lateral and ventral view, which clearly shows that the endostyle and CSG are symmetrical in *Pitxa/c* homozygotes (**b’**). m, mouth; pp: preoral pit; en, endostyle; csg, club-shaped gland; g: gill slit. In situ hybridization to WT/heterozygotes and *Pitxa/c* homozygotes at N5 and T1 stage for *Lhx3* (**c**, **c’**, **d**, **d’**), *Prop1* (**e**, **e’**, **f**, **f’**), and *Pit* (**g**, **g’**, **h**, **h’**). **i, i’** In situ hybridization to WT/heterozygotes and *Pitxa/c* homozygotes at T1 stage for *Pou4*. Black arrowheads indicate the anterior preoral pit region and red arrowheads indicate the posterior preoral pit region , arrows indicate the mouth region. In situ hybridization to WT/heterozygotes and *Pitxa/c* homozygotes at N5 and T1 stage for *Nkx2.1* (**j**, **j’**, **k**, **k’**), *Hex* (**l**, **l’**, **m**, **m’**), *FoxE4* (**n**, **n’**, **o**, **o’**), and *Krox* (**p**, **p’**, **q**, **q’**). In (**k’**), the black arrowhead shows the expression boundary of *Nkx2.1* on the right side and red purple arrowhead shows expressing boundary on the left side. Images are dorsal views in **c**–**q’**. Numbers in the bottom right corner of a panel show the number of times the phenotype depicted was observed, out of the total number of embryos from that genotype analyzed. A, anterior; P, posterior; D, dorsal; V, ventral; L, left side; R; right side. Scale bars: 50 μm

We also crossed heterozygous *Pitxc* animals (carrying mutation at the TALEN1 site) with heterozygous *Pitx* animals carrying mutations at the TALEN2 site or the TALEN3 site. Unexpectedly, in both crosses, we found around a quarter of the generated embryos showing a milder (compared to *Pitx*^−/−^ mutants), but specific phenotype at stage L3. Genotypic analysis confirmed that individuals showing this phenotype carry mutations at both TALEN1 site and TALEN2 or TALEN3 site (TALEN1^+/−^; TALEN2^+/−^ or TALEN1^+/−^; TALEN3^+/−^), while others of normal morphology harbor mutations only in one or in neither of the TALEN1 site and the TALEN2 or TALEN3 site (TALEN1^+/+^; TALEN2^+/+^, TALEN1^+/−^; TALEN2^+/+^, TALEN1^+/+^; TALEN2^+/−^, TALEN1^+/+^; TALEN3^+/+^, TALEN1^+/−^; TALEN3^+/+^, or TALEN1^+/+^; TALEN3^+/−^) (Additional file 1: Fig. S9, S10). For simplification, we hereafter refer to these two types of embryos as *Pitxa/c* homozygotes and WT/heterozygotes respectively and use embryos from crosses between TALEN1 and TALEN2 animals for further analysis. Compared to WT/heterozygotes (Fig. 5a, a’), *Pitxa/c* homozygotes had no mouth opening and malformed preoral pit but formed an endostyle and glandular region of the CSG on each side (Fig. 5b, b’). Importantly, their gill slits still formed and positioned normally as that of WT/heterozygotes (Fig. 5a’, b’).

**Fig. 6:**
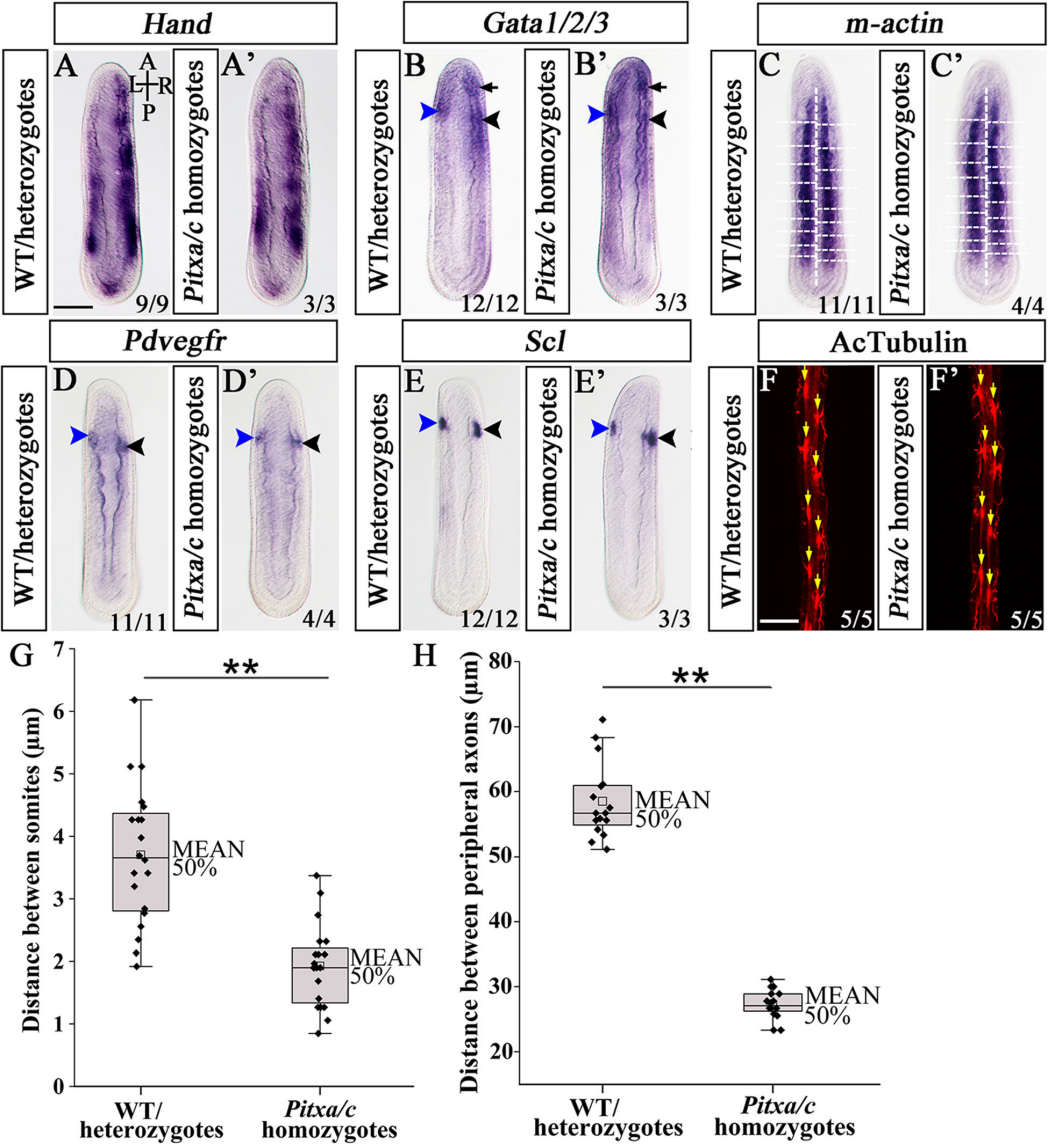
Arrangement of mesoderm-derived organs and peripheral nerves in partial loss of *Pitx* function. In situ hybridization to WT/heterozygotes and *Pitxa/c* homozygotes at N5 stage for *Hand* (**a**, **a’**), *Gata1/2/3* (**b**, **b’**), *Pdvegfr* (**d**, **d’**), and *Scl* (**e**, **e’**). Blue arrowheads are Hatschek’s nephridium and black arrowheads are hematopoietic region. Black arrows are the HRD. **c**, **c’** The arrangement of somites is marked by *m-actin* in N5 stage embryos. Longitudinal dashed lines indicate notochord, transversal dashed lines mark somite outlines. **f**, **f’** Yellow arrows indicate peripheral nerves marked by acetylated α-tubulin. Quantification of the distance between a pair of somites (**g**), and the distance between a pair of peripheral nerves (**h**). Posterior five pairs of somites are used for measure in one embryo, and four pairs of peripheral nerves shown in the image are used for measure. Significance determined by *t*-test is indicated by asterisks: **P < 0.01. Embryos are all dorsal views. Numbers in the bottom right corner of a panel show the number of times the phenotype depicted was seen, out of the total number of embryos from that genotype analyzed. A, anterior; P, posterior; L, left side; R; right side. Scale bars: 50 μm

To further clarify the phenotype of *Pitxa/c* homozygotes, we examined expression of marker genes of LR organs in the mutants at N5 and T1 stages. *Pitxa/c* homozygotes lacked *Pou4* expression (marker of the mouth) on the left side (Fig. 5i, i’, arrow), but expressed *Nkx2.1* (Fig. 5j, k) and *Hex* (Fig. 5l, m) (markers of the endostyle), and *FoxE4* (Fig. 5n, o) and *Krox* (Fig. 5p, q) (markers of the CSG) nearly symmetrically on both sides (Fig. 5j’–q’) as observed in *Pitx*^−/−^ mutants (Fig. 3h’–o’). Interestingly, *Lhx3*, *Prop1*, and *Pit* expression in the posterior preoral pit region (Fig. 5c–h, red arrowhead) was lost in *Pitxa/c* homozygotes (Fig. 5c’–h’) as in *Pitx*^−/−^ mutants (Fig. 3a’–f’), while that of *Lhx3* and *Prop1* in the anterior part of the organ remained in the former (Fig. 5c’–f’, black arrowhead) but was lost in the latter (Fig. 3a’–d’). The asymmetric expression of *Hand* in the lateral/ventral mesoderm (Fig. 6a, a’), *Gata1/2/3*, *Pdvegfr*, and *Scl* in Hatschek’s nephridium (Fig. 6b, d, e, blue arrowhead) and hematopoietic domain (Fig. 6b, d, e, black arrowhead), and *Gata1/2/3* in the HBD (Fig. 6b, arrow) were not affected in *Pitxa/c* mutants (Fig. 6b’, d’, e’), while that of *m-actin* in the somites (Fig. 6c, c’, g) and acetylated tubulin in the peripheral nerves (Fig. 6f, f’, h) was weakened as observed in *Pitx*^−/−^ mutants (Fig. 4i–n).

Together, the above results indicated that the mutation at the TALEN1 site actually affected *Pitxc* function, albeit very mildly, and that the development of various LR organs are of different sensibility to *Pitx* activity reduction. Among them, the mouth, endostyle, CSG, posterior part of the preoral pit, and the asymmetry of the somites and peripheral nerves were sensitive to an intermediate level of *Pitx* activity reduction (*Pitxa/c* homozygotes), while the lateral/ventral mesoderm, Hatschek’s nephridium, hematopoietic domain, HBD, and the anterior part of the preoral pit were sensitive to total loss of *Pitx* activity (*Pitx*^−/−^), with none of them sensitive to a low level of *Pitx* activity reduction (*Pitxc*^−/−^).

### *Pitx* controls development of pharyngeal LR asymmetric organs by directly activating genes essential for left-side organs and repressing genes of right-side organs on the left side

**Fig. 7:**
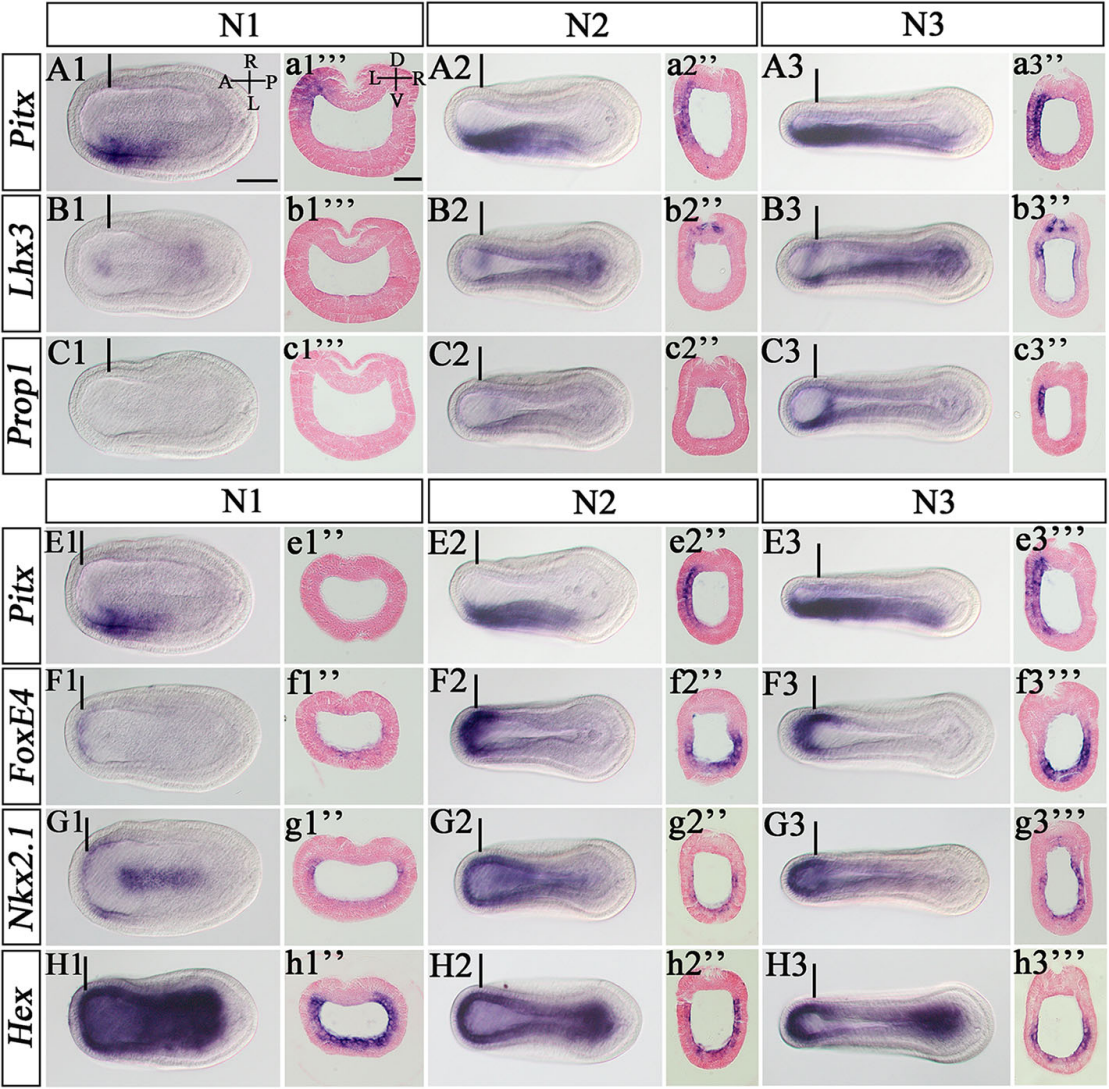
Expression pattern of *Pitx* and asymmetrical pharyngeal organs markers. **a1–c3**, **e1-e3** *Pitx* is expressed in the left anterior-dorsal half of the embryos from the N1 neurula stage to N3 neurula stage (**a1-a3**, **e1-e3**), *Lhx3*, *Prop1* is activated in the prospective preoral pit region from the N1 stage to N3 stage (**b1-b3**, **c1-c3**), as shown by WISH and transvers sections in (**a1”’–c3”**). **f1–h3** The expression of right-sided organ makers, *FoxE4* (**f1-f3**), *Nkx2.1* (**g1-g3**), and *Hex* (**h1-h3**) from N1 to N3 stage, as shown by WISH and transverse sections (**f1”–h3”’**). The lines in **a1–h3** indicate the position of transverse sections of the embryos. The embryos detected by WISH are all in dorsal views. A, anterior; P, posterior; D, dorsal; V, ventral; L, left side; R; right side. Scale bars: 50 μm

Among LR asymmetric organs within the amphioxus pharynx, the preoral pit and endostyle are thought to be homologs of the vertebrate pituitary and thyroid respectively [43, 44], while the CSG is an amphioxus-specific organ transiently present at larval stages. *Lhx3*-*Prop1*-*Pit* and *Hex*-*Nkx2.1* are required for the development of pituitary [43, 45, 46] and thyroid in vertebrates respectively [44, 47], and *FoxE4* is one of the earliest transcription factors activated specifically in the CSG [48]. To dissect how *Pitx* regulated development of the preoral pit, endostyle, and CSG in amphioxus, we examined the expressions of these genes and compared them with that of *Pitx* in embryos between N1 stage, when *Pitx* begins to express asymmetrically on the left side [24], and N4 stage, when LR morphogenesis is thought to initiate [28]. At the N1 stage, *Pitx* was detected in the left dorso-anterior half of the embryos (Fig. 7a1, a1”’), while *Lhx3* was weakly and *Prop1* was not yet activated in the left anterior endoderm that will later become preoral pit (Fig. 7b1, b1”’, c1, c1”’). At this stage, *FoxE4* began to be transcribed in the anterior most endoderm (Fig. 7f, f1”) where *Pitx* is not expressed (Fig. 7e1,e1”). *Nkx2.1* and *Hex* expression in the anterior mesendoderm, from which the endostyle will later form, are symmetrically activated from the early neurula stage [44, 47]. However, at the N1 stage, the expression levels of both genes in the left anterior paraxial mesoderm were somewhat lower than that in the right paraxial mesoderm (Additional file 1: Fig. S11g1, g1”’, h1and h1”’). At N2 stage, *Pitx* expression was enhanced and expanded both anteriorly, posteriorly and ventrally, forming a gradient along these axes (Fig. 7a2, a2”, e2 and e2”, Additional file 1: Fig. S11a2’-a2”’, e2’-e2”’). Along with this, *Lhx3* and *Prop1* were both transcribed in the region where the preoral pit will later form (Fig. 7b2, b2”, c2 and c2”), and *FoxE4*, *Nkx2.1*, and *Hex* were further downregulated on the left side (Fig. 7f2, f2”, g2, g2”, h2 and h2”). Interestingly, *FoxE4*, *Nkx2.1*, and *Hex* also showed a gradient of expression on the left side along the dorsal-ventral and anterior-posterior axis (Additional file 1: Fig. S11f2’-f2”’, g2’-g2”’, h2’-h2”’), which were inversely correlated to the gradient of *Pitx* expression (Additional file 1: Fig. S11e2’-e2”’). By N3 stage, *Pitx* expanded its expression domain more ventrally and posteriorly (Fig. 7a3, a3”, e3, and e3”’, Additional file 1: Fig. S11a3’-a3”’, e3’-e3”’), and the asymmetric expression of *Lhx3* and *Prop1* on the left side (Fig. 7b3, b3”, c3, c3”, Additional file 1: Fig. S11b3’-b3”’, e3’-e3”’) and that of *FoxE4*, *Nkx2.1*, and *Hex* on the right side became more pronounced (Fig. 7f3”’, g3”’ , h3”’, Additional file 1: Fig. S11f3’-f3”’, g3’-g3”’ , h3’-h3”’). Compared to those genes, *Pit* expression in the posterior part of the preoral pit and *Krox* expression in the endostyle were activated relatively late and became detectable until N4 stage (Additional file 1: Fig. S12).

**Fig. 8:**
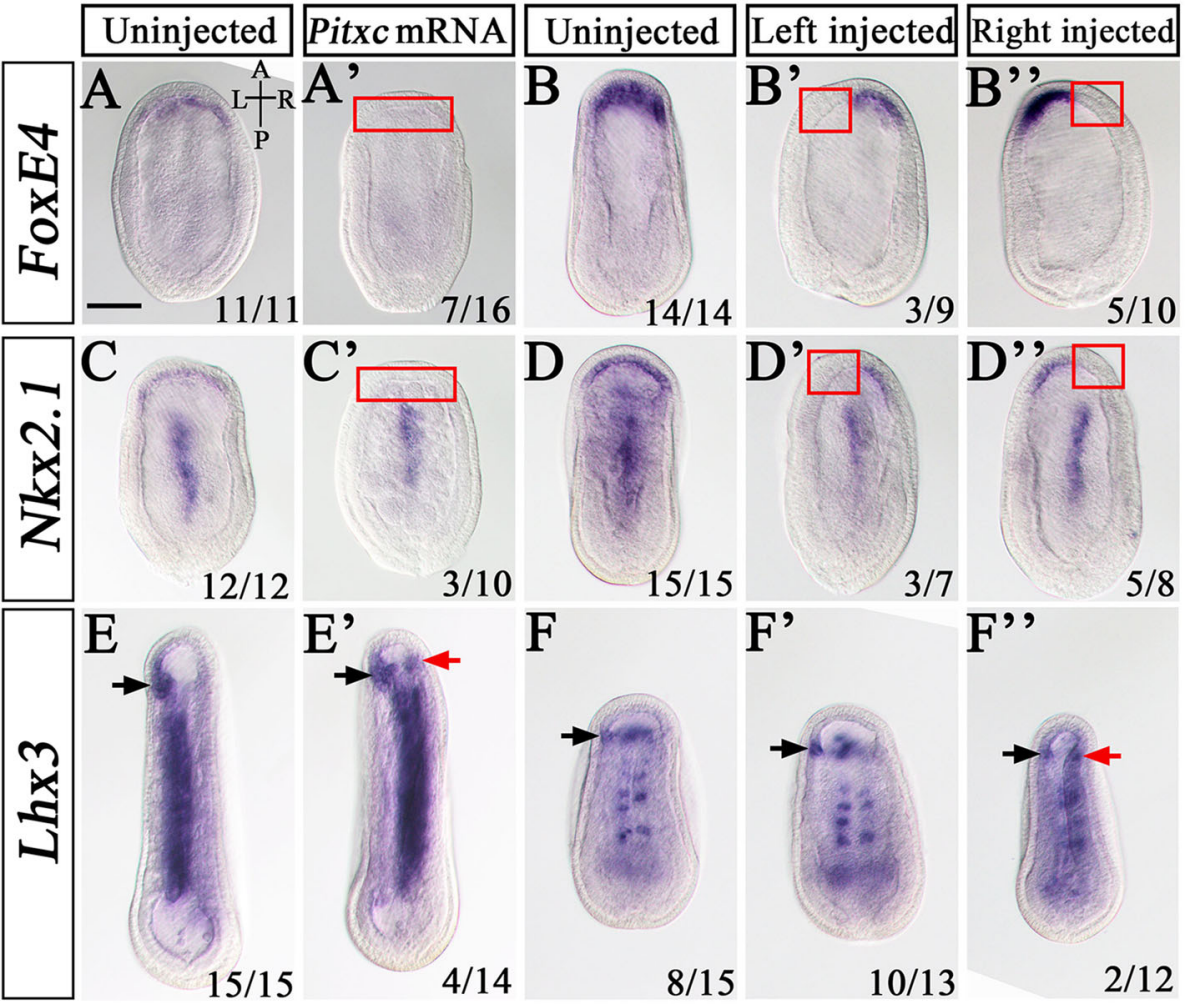
Overexpression of *Pitx* can activate *Lhx3* expression and inhibit the expression of *FoxE4* and *Nkx2.1*. **a–f** These control groups are not injected with *Pitxc* mRNA. **a’–e’** These groups are injected with *Pitxc* mRNA at the unfertilized egg stage. **b’–f”** *Pitxc* mRNA is injected into one blastomere at 2-cell stage. Red boxes indicate the region in which *FoxE4* or *Nkx2.1* expression disappeared. The black arrows indicate left preoral pit marked by *Lhx3* and red arrows indicate the new induced preoral pit. The embryos detected is at N1 stage in (**a**, **a’**, **c**, **c’**), N2 stage in **b–b”**, **d–d”**, **f–f”**, N4 stage in **e**, **e’**. Numbers in the bottom right comer of a panel show the number of times in phenotype was observed in total number of embryos examined. The remaining injected embryos not shown showed similar gene expression patterns like the uninjected embryos. All the images are dorsal view. A, anterior; P, posterior; L, left side; R; right side. Scale bars: 50 μm

The activation of *Lhx3* and *Prop1* and downregulation of *FoxE4*, *Nkx2.1* and *Hex* in areas of *Pitx* expression suggests that *Pitx* could induce expression of the former while inhibiting expression of the latter. To test this, we first examined the expression of these genes in *Pitx*^−/−^ mutants at N2 and N3 neurula stages (Additional file 1: Fig. S13). As described above, *FoxE4*, *Nkx2.1*, and *Hex* were asymmetrically expressed in the anterior endoderm of WT and *Pitx*^+/−^ embryos in an apparently L < R manner at the N2 stage (Additional file 1: Fig. S13a, a’-a”’, c, c’-c”’, e, e’-e”’). However, in *Pitx*^−/−^ mutants the asymmetric expression pattern of these genes was somewhat weakened and became less pronounced due to an upregulation of their expression on the left side (Additional file 1: Fig. S13b, b’-b”’, d, d’-d”’, f, f’-f”’). At the N3 stage, asymmetry of *FoxE4*, *Nkx2.1*, and *Hex* expression became more pronounced in WT and *Pitx*^+/−^ embryos (Additional file 1: Fig. S13g, g’-g”’, i, i’-i”’, k, k’-k”’), but were still ambiguous in *Pitx*^−/−^ mutants due to a failure of downregulation on the left side (Additional file 1: Fig. S13h, h’-h”’, j, j’-j”’, i, i’-i”’). At this stage, both *Lhx3* and *Prop1* expression were not activated in the presumptive preoral pit of the *Pitx*^−/−^ mutants (Additional file 1: Fig. S13m-p). To further clarify the speculation above, we injected unfertilized amphioxus eggs with *Pitxc* mRNA and examined its effect on the expression of *FoxE4*, *Nkx2.1,* and *Lhx3* genes. *FoxE4* was expressed specifically in the anterior endoderm of all (11/11) examined uninjected embryos (Fig. 8a). However, after injection of *Pitxc* mRNA, 43.8% (7/16) of embryos showed no *FoxE4* expression in the corresponding region (Fig. 8a’). A similar case was observed for *Nkx2.1* (Fig. 8c, c’). Different to these, injection caused 28.6% (4/14) of embryos to express *Lhx3* ectopically in the right anterior endoderm (Fig. 8e’, red arrow). We also injected *Pitxc* mRNA into one blastomere at a 2-cell stage, divided them into left-side injected and right-side injected ones at late gastrula stage, and then assayed the expression of the above genes. Among left-side injected embryos, 33.3% (3/9) and 42.9% (3/7) of them showed no *FoxE4* and *Nkx2.1* expression in the left anterior endoderm, respectively (Fig. 8b, b’, d, d’), while 76.9% (10/13) exhibited *Lhx3* expression in the left-side presumptive preoral pit region, which is significantly more than that (53.3%, 8/15) observed in the uninjected embryos (Fig. 8f, f’, arrow). In contrast, among right-side injected embryos, 50% (5/10) and 62.5% (5/8) of them respectively lost *FoxE4* and *Nkx2.1* expression in the right anterior endoderm (Fig. 8b, b”, d, d”), while 16.7% (2/12) showed ectopic *Lhx3* expression in the right side (Fig. 8f, f”, red arrow). Together, these results indicated that *Pitx* was required and sufficient for inducing *Lhx3* and *Prop1* expression and inhibiting *FoxE4*, *Nkx2.1*, and *Hex* expression in amphioxus embryos.

**Fig. 9:**
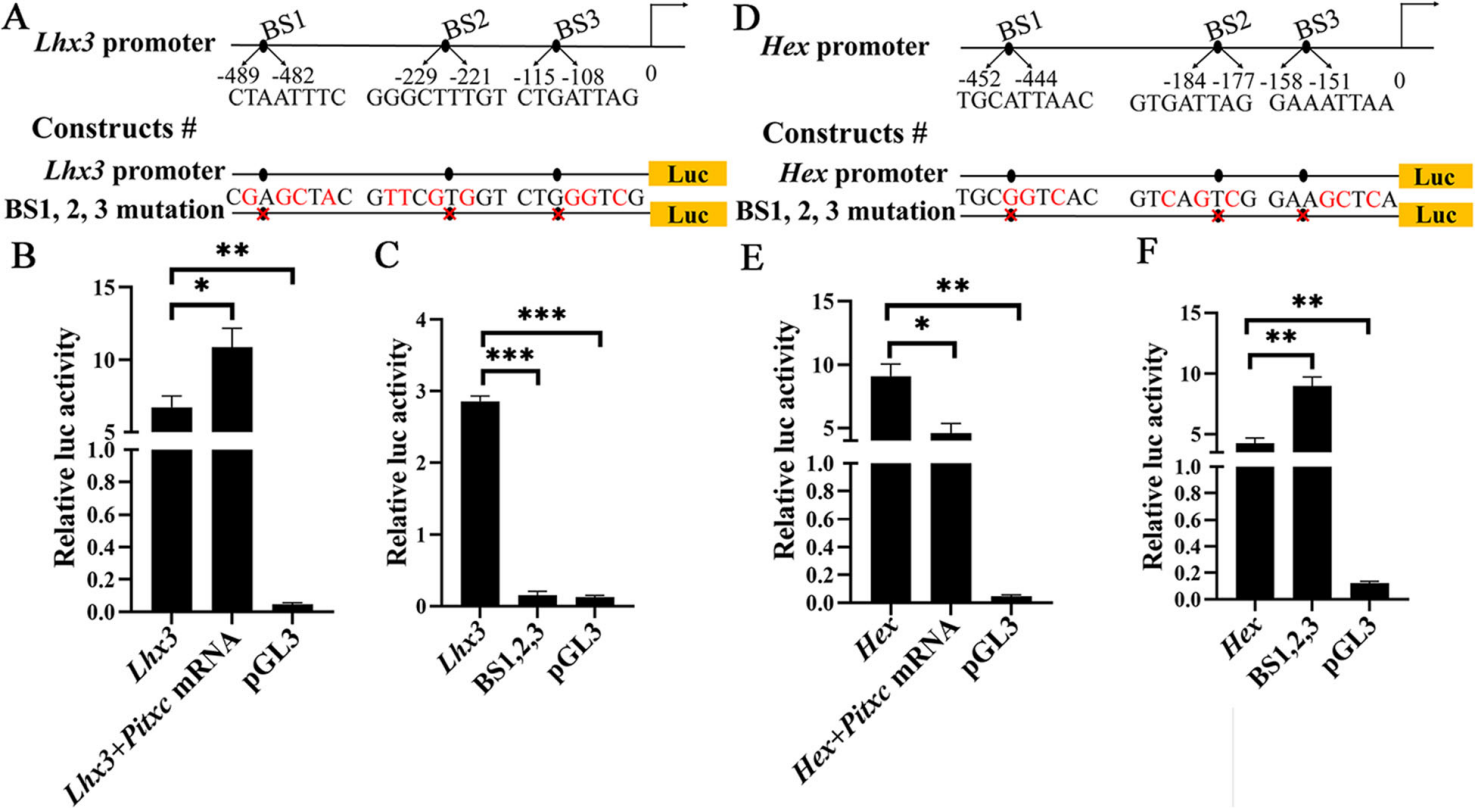
Luciferase assay analysis of *Lhx3* and *Hex* regulatory sequences. **a**, **d** Region 5′ of *Lhx3* gene (**a**) or *Hex* gene (**d**) shows three putative *Pitx* binding sites (BS1, BS2, BS3). Numbers show the distance before the start codon. Two luciferase reporter gene constructs are tested in amphioxus embryos with 0 or 3 *Pitx* binding sites mutated in *Lhx3* (**a**) or *Hex* (**d**) promoter. **b**, **e** Histogram shows relative levels of luciferase expression for a construct including 0 *Pitx* binding sites mutated, a group co-injection of *Pitxc* mRNA with the construct, a control luciferase vector pGL3 without *Lhx3* or *Hex* sequence. **c**, **f** Histogram shows relative levels of luciferase expression for each construct (0 or 3 *Pitx* binding sites mutated), a control luciferase vector pGL3. Significance determined by *t*-test is indicated by asterisks: *P < 0.05, **P < 0.01, ***P < 0.001

*Lhx3* and *Hex* are upstream transcription factors for the development of vertebrate pituitary and thyroid, respectively [49, 50], and are among the earliest genes activated in the amphioxus preoral pit and endostyle, respectively [45, 47]. We therefore speculated that *Pitx* regulated the development of the preoral pit and endostyle through directly regulating the expression of *Lhx3* and *Hex*. To test this, we analyzed upstream sequences of the two genes and identified three *Pitx* binding sites for each gene (Fig. 9a, d, Additional file 1: Table S1). Luciferase assay showed that both sequences were able to drive significantly higher levels of luciferase activity than the control (Fig. 9b, c, e, f). Co-injection of *Pitxc* mRNA with the constructs increased luciferase activity of *Lhx3* construct (Fig. 9b), but decreased luciferase activity of *Hex* construct (Fig. 9e). Moreover, mutation of the *Pitx* binding sites abolished activity of *Lhx3* sequence (Fig. 9c), but increased activity of *Hex* sequence (Fig. 9f). These results suggested that *Lhx3* and *Hex* were likely direct transcriptional targets of *Pitx* in amphioxus, and *Pitx* binding activated *Lhx3* expression, while *Pitx* binding repressed *Hex* expression.

## Discussion

Requirement of *Pitx2* for LR morphogenesis has been demonstrated in mice by gene knockout studies [7–10] and in chick and frogs using gene knockdown strategies [7–10]. However, its role in zebrafish LR morphogenesis is still under debate. Using a morpholino knockdown method, Liu and Semina found no LR defects in zebrafish *Pitx2* morphants [16], while Garric et al. reported a weak phenotype in habenular asymmetry [17]. In a later study, Ji et al. confirmed the finding of Liu and Semina by examining several lines of *Pitx2* mutants and revealed that these mutants are able to survive to adults without any LR defects [15, 16]. Outside vertebrates, *Pitx* function has not been examined, although its asymmetric expression and regulation by Nodal signaling pathways have been reported in several invertebrate animals [24–28]. We here demonstrate that *Pitx* is required for LR morphogenesis in the chordate amphioxus. This indicates that involvement of *Pitx* in LR development predates chordate diversification, and also strengthens the importance of the gene in this process.

Although *Pitx* function in asymmetric development is generally conserved among chordates, the dependence of the gene in their LR morphogenesis is apparently different. In amphioxus, *Pitx* mutation affects asymmetries of most if not all LR organs, and causes loss of all left-side organs and ectopic formation of right-side structures on the left side. Whereas *Pitx2* deficiency in zebrafish leads to no (or very weak) LR phenotype as mentioned above, and *Pitx2* loss-of-function in mice causes right isomerism of the lungs and atrium, and defects in other visceral organs, but the position of the stomach and heart looping remain unaffected [8, 51–55]. These differences reflect that LR organogenesis in amphioxus relies more heavily on *Pitx* than that in vertebrates, and that LR organogenesis in vertebrates may involve more regulatory factors.

*Pitx2c* is the only isoform of *Pitx2* showing an asymmetric expression pattern in vertebrates [34]. Mice specially lacking asymmetric *Pitx2c* expression manifest LR defects comparable to *Pitx* knockout mice [8, 11], indicating that *Pitx2c* is a major if not the only player out of the three *Pitx2* isoforms in mouse LR development. Similarly, we identified two *Pitx* isoforms in amphioxus: *Pitxa/b* and *Pitxc*, among which the amphioxus *Pitxc* showed asymmetric expression in embryos while *Pitxa/b* did not. However, unlike *Pitx2c* in mice, *Pitxc* mutation in amphioxus does not cause any LR defects. One possible interpretation of this discrepancy is that the mutation we introduced is hypomorphic. Indeed, *Pitxa/c* homozygotes from a cross between *Pitxc* heterozygotes and *Pitx* heterozygotes manifested LR defects at the larval stages, and importantly these defects were much milder compared to that observed in *Pitx*^−/−^ mutants. Furthermore, there are two alternative in-frame start codons immediately after the mutation site we introduced in the *Pitxc* locus (Additional file 1: Table S2). These findings suggest that the *Pitxc*^−/−^ mutants may use one of the two alternative start codons to make a dampened version of *Pitxc* protein. Interestingly, *Pitxc* expression was slightly upregulated in *Pitxc*^−/−^ mutants (Additional file 1: Fig. S14d) compared to their WT/*Pitx*^−/−^ sibling (Additional file 1: Fig. S14c), while *Pitxa/b* expression remained unchanged (Additional file 1: Fig. S14a, b). We suggest this upregulation can, to some extent, compensate dampened *Pitxc* function and contribute toward normal development of *Pitxc*^−/−^ embryos.

Our study provides several lines of evidence indicating that *Pitx* controls amphioxus LR morphogenesis by promoting development of organs on the left side and repressing formation of organs on the right side. First, *Pitx* mutation in amphioxus resulted in loss of left-side organs and ectopic formation of right-side organs on the left side. Second, genes essential for left-side organs like *Lhx3* and *Prop1* were activated after *Pitx* expression and their expression was observed in the region where *Pitx* was highly expressed, while genes required for the formation of organs on the right side, at least for the endostyle and CSG, were initiated either earlier (like *Hex*) or later (like *FoxE4*) than *Pitx*, and their expression was observed in the region where *Pitx* was not expressed. Importantly, the right-side genes were initially symmetrically expressed, and they became asymmetrically expressed (decreased expression on the left side) only when *Pitx* expression expanded into their expression domain. Third, while *Pitx*^−/−^ mutants showed no expression of left-side genes and nearly bilaterally symmetric expression of right-side genes, *Pitx* overexpression induced expression of the left-side genes and inhibited that of the right-side genes. Fourth, *Hex* and *Lhx3* genes, upstream regulators of the endostyle and preoral pit, respectively, had *Pitx* binding sites in their regulatory sequences, and mutation of these sites increased the activity of the *Hex* sequence, but decreased that of the *Lhx3* sequence. Lastly, although BMP signaling pathway was thought to be a determinant of right-side development in vertebrates [20], our results demonstrated that this was not the case in amphioxus, as inhibition of BMP signaling after the N1 stage did not affect the asymmetrical expression pattern of *Pitx*, but reduced its expression on the left side (Additional file 1: Fig. S15d-i, d’-i’). However, it should be noted that blocking the BMP signal from late gastrula stage in amphioxus embryos eliminated *Pitx* expression on both sides (Additional file 1: Fig. S15b, b’, c, c’), which is probably caused by ectopic induction of Nodal inhibitor *Dand5* on the left side [56]. We are uncertain if vertebrates use a similar mechanism to pattern their LR organs in general, but we speculate that this may be the case in lungs and atria since *Pitx2* is expressed in the left side of these organs in mice, and its deficiency results in right isomerism of these organs at the expense of their left-sided structures [8, 51–55]. Consistent with this, a previous study showed that *Pitx2c* inhibited the development of sinoatrial node (a structure derived from the right atrium) by directly repressing *Shox2*, a transcriptional regulator of sinoatrial node gene program [57].

We showed that, as in vertebrates [3], LR morphogenesis in amphioxus required *Pitx*-independent signals (factor X) downstream of Nodal signaling. *Bmp2/4* is recently shown to be asymmetrically expressed in amphioxus embryos from N1 to N2 stage in a L > R manner, and inhibition of Nodal signaling at the gastrula stage eliminates *Bmp2/4* expression [56]. This suggests a possibility for *Bmp2/4* to be the X factor. To test this, we treated *Pitx* mutants with BMP inhibitor DMH1 from N0 to N5 stage or N0 to T1 stage and assayed the expression of *Krox* (marker of the glandular region of the CSG), *Nkx2.1* (marker of the endostyle), and *m-actin* (marker of the somites). The treatment abolished the expression of the *Krox* gene in *Pitx*^−/−^ mutants and their siblings at the N5 stage (Additional file 1: Fig. S16m, n), indicating that BMP signaling pathway was required for *Krox* expression. However, the treatment did not affect the expression of *Nkx2.1* and *m-actin* in either *Pitx*^−/−^ mutants (Additional file 1: Fig. S16p, t) or their siblings (Additional file 1: Fig. S16o, s). As a positive control, the treatment of mutants with the Nodal signaling inhibitor SB505124 at the same stages caused symmetric expression of *Krox* and *Nkx2.1* in all examined embryos (*Pitx*^−/−^ mutants and their sibling) (Additional file 1: Fig. S16e-h). NOTCH, FGF, and RA play essential roles in the generation of somites from the tailbud of vertebrate embryos [33, 58]. Specifically, inhibition of RA signaling activity in zebrafish, mouse, and chicken led to asymmetric somites [59, 60]. Different from vertebrates, RA and FGF signaling are not required for the formation and asymmetric patterning of somites from the tailbud in amphioxus [33]. However, this does not exclude the possibility of the signals as the X factor. To test this, we conducted similar experiments as described above and found that none of them appeared to be the X factor as treatment of *Pitx* mutants with inhibitors of NOTCH (DAPT), FGF (SU5402), and RA (BMS493) signaling pathways did not affect the expression patterns of *m-actin* (Additional file 1: Fig. S17k-p) and *Nkx2.1* (Additional file 1: Fig. S16c-h) genes. Further studies are required to identify the nature of the X signal in the future.

The homologous relationship between the amphioxus preoral pit and vertebrate adenohypophysis was first indicated by anatomical studies [61], and later strengthened by gene expression data [43, 62]. We here demonstrate that similar to vertebrates [63], *Pitx* is able to direct the development of amphioxus preoral pit through controlling expression of *Lhx3* and/or *Prop1* genes. This further confirms the homologous relationship of the two organs. However, it is striking that the preoral pit is located on the left side of amphioxus while the adenohypophysis is formed at the anterior midline region of vertebrate embryos [43, 64]. As *Pitx* is a master regulator of the two organs, we speculate that this difference is probably related to changes in *Pitx* expression between amphioxus and vertebrates. Previous studies showed that inhibition of Nodal signaling before 8S stages abolished *Pitx* expression in amphioxus embryos [24]. We here further showed that *Pitx* expression relies on the Nodal signaling pathway at least until stage T1. This demonstrates that Nodal signaling is absolutely required for *Pitx* expression in amphioxus not only in the LR patterning phase, but also in early stage of LR morphogenesis. Vertebrates have evolved three *Pitx* genes (*Pitx1*, *Pitx2* and *Pitx3*) by two rounds of whole genome duplications after their divergence from cephalochordates [65]. Among them, *Pitx1* and *Pitx2* are expressed in the adenohypophysis and are both required for its development [66, 67]. However, their expressions in the adenohypophysis are regulated by signaling pathways other than Nodal [68]. Acquirement of Nodal-independent regulation of *Pitx* genes during vertebrate evolution is therefore likely a key event enabling the shift of the adenohypophysis to the midline in vertebrates.

Somites are unique to amphioxus and vertebrates within the chordates. Although vertebrate somites and the posterior somites of amphioxus are both generated from the tailbud, their formation mode and the patterning of them along the LR axis are different. In vertebrates, somites are formed symmetrically under a clock and wavefront mechanism [33, 58], whereas amphioxus lacks the mechanism and develops the posterior somites asymmetrically [69]. RA signal is required for symmetric somitogenesis of vertebrates through buffering the lateralizing influence of the LR machinery [59, 60]. Application of RA or BMS009 (an antagonist of the RA receptor) does not affect the asymmetric patterning of amphioxus somites, indicating RA signaling pathway is not involved in the process [33]. However, different from vertebrates, *Pitx* is continuously expressed in the left side of tailbud from the 8-somite stage during amphioxus development, and this expression relies strictly on the Nodal activity there. Moreover, inhibition of the Nodal activity after 8-somite stages or knockout of *Pitx* affects the asymmetry of amphioxus somites. Therefore, Nodal signal and *Pitx*, but not RA signal, are responsible for the asymmetric generation of somites in amphioxus. Considering that vertebrates have evolved a much more sophisticated process in somitogenesis than amphioxus, we speculate that the acquisition of RA signal, together with the loss of Nodal-Pitx cascade, in vertebrates might be the cause for their symmetric somitogenesis.

## Conclusions

**Fig. 10:**
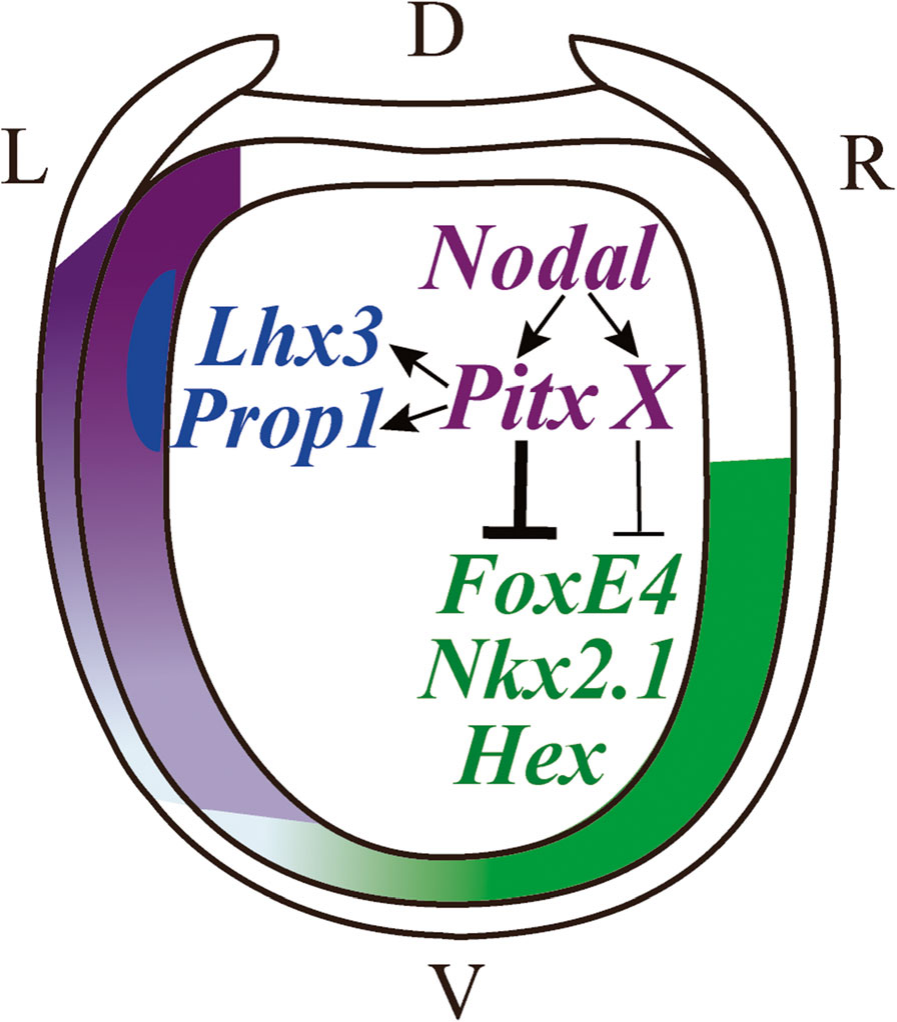
A model for *Pitx* controlling the asymmetry of pharyngeal organs. At early neurula stage (before N4 stage), *Pitx* expression (gradient purple region) is in a gradient manner along the dorsal-ventral axis. During the process, *Pitx* promotes the development of organs on the left through directly activating expression of *Lhx3*, *Prop1*, or/and *Pit* (blue region). Meanwhile, *Pitx* is a dominant factor together with an unknown factor X, and both factors are located downstream of Nodal signaling pathway, inhibiting the formation of right-side organs by directly repressing expression of *Hex*, *Nkx2.1*, or/and *FoxE4* (gradient green region) on the left side. D, dorsal; V, ventral; L, left side; R, right side

In summary, our study reveals that *Pitx* is a dominant (but not the only) factor downstream of Nodal signaling, in the LR morphogenesis of amphioxus (Fig. 10). During the development of amphioxus pharyngeal organs, *Pitx* promotes the development of left-side organs by directly activating expression of genes like *Lhx3* (and/or *Prop1* and *Pit*) (Fig. 10). Simultaneously, it acts together with an unidentified factor X, also downstream of Nodal signaling, to inhibit formation of right-side organs by directly repressing expression of genes like *Hex* (and/or *Nkx2.1* and *FoxE4*) in a dosage manner (Fig. 10). This provides essential clues for understanding the mechanisms of *Pitx2* in the regulation of asymmetric organogenesis in vertebrates, and also sheds important insights on why some organs like adenohypophysis are asymmetrically positioned in the amphioxus, but are symmetrically placed in vertebrates.

## Methods

### Animals and embryo cultivation

Amphioxus *Branchiostoma floridae* were obtained from Dr. Jr-Kai Yu’s laboratory at the Institute of Cellular and Organismic Biology, Academia Sinica, Taiwan. They were maintained and induced to spawn following the protocol as we described and used in *B*. *belcheri* [70, 71], which is slightly different from the protocol recently developed for *B*. *floridae* [37]. Egg fertilization and embryo culture were carried out according to our previous report [72]. Embryos are staged according to a recent study unless otherwise stated [73, 74].

### Isolation of *Pitx* isoforms in amphioxus

*Pitxc* isoform sequence was assembled from in-house generated transcriptome data [75], and *Pitxa/b* isoform (accession number: FE578121) was acquired by searching publicly available EST data in the NCBI database using Blast with the *Pitxc* sequence as the query. Three primers, two located in the first exons of *Pitxc* and *Pitxa/b* and one located in their common region (2nd exon of *Pitxc* and 3rd exon of *Pitxa/b*) were designed to verify their existence in amphioxus embryos. Exon/intron structures of the two isoforms were determined by aligning their mRNA sequences to the corresponding genome sequence on UCSC (genome-asia.ucsc.edu).

### Mutant generation and genotyping

Generation and detection of *Pitx* mutants was conducted using the TALEN method as previously described [24, 36, 37, 75]. TALEN target sites of *Pitx* and mutation types used in the study are shown in Fig. 1. Primers for amplification of the flanking regions of the *Pitx* target sites and their amplicon sequences and sizes are listed in Additional file 2: Table S1. Genotyping of live embryos and embryos examined by whole mount in situ hybridization was performed as previously described [32, 37].

### Whole-mount in situ hybridization, histology, and immunofluorescence analysis

pGEM-T-Easy constructs containing *Cer*, *Nodal*, *Lefty*, *Pitxc*, *Lhx3*, *Krox*, *FoxE4*, *Nkx2.1*, *Pit*, *Pou4*, *m-actin*, *Mop*, and *Pax2/5/8* mRNA sequences were obtained in previous studies [24, 31, 76, 77]. pGEM-T-Easy constructs containing partial or complete mRNA sequences of *Hex*, *Prop1*, *Gata1/2/3*, *Pdvegfr*, *Scl*, *Hand*, *Pitxa/b*, and *Pitxc* genes were constructed using protocols as previously reported [24, 31]. Primers used for gene cloning are listed in . Digoxigenin (DIG)-labeled antisense riboprobes (Roche) of all above genes were synthesized with Sp6 or T7 RNA polymerase (Thermo). Sequences are deposited in GenBank and their accession numbers are available in Additional file 2: Table S2.

Embryos at desired stages were fixed overnight with 4% (wt/vol) PFA-MOPS-EGTA (pH 7.5) at 4 °C, and then stored at − 20 °C in 70% ethanol (vol/vol) for use. Whole-mount in situ hybridization (WISH) was performed following the description by Yu and Holland [78]. For the three *Pitx* gene probes, the one (called *Pitx* probe) transcribed from *Pitxc* isoform (indicated by the green line in Fig. 1a) was used for most experiments unless otherwise stated. Stained embryos were photographed using an inverted microscope (Olympus, IX71). After imaging, embryos were embedded in agarose-paraffin, sectioned at 5 μm with a Leica RM2016 microtome, and then photographed using a brightfield microscope (Zeiss, AX10).

Acetylated α-tubulin immunofluorescence staining for detection of peripheral nerves was performed as previously described [32]. Before staining, embryos and larvae were de-ciliated by a short incubation in 0.83 M NaCl seawater and then fixed in 4% (wt/vol) PFA-MOPS-EGTA (pH 7.5) at 4 °C overnight [32]. DAPI (Invitrogen, 1 μg/ml in PBST) was used for nuclear staining. Images of stained embryos were acquired using a Zeiss LSM 780 confocal microscope.

### Quantification and statistical analysis

The distance between somites or peripheral axons was measured using ImageJ software. Scatter boxplots were drawn using OriginPro (OriginLab Corporation). Statistical significance was tested by independent-samples *t*-tests with SPSS Statistics version 19 (IBM Corporation).

### mRNA synthesis and embryo microinjection

A pXT7 construct containing *Pitxc* partial 5′UTR (207-bp), full-length coding sequence, and partial 3′UTR (217-bp) was conserved in a previous study [24]. *Pitxc* mRNAs was synthesized from the construct with T7 mMESSAGE mMACHINE kit (Ambion) following the kit manual. The mRNAs (300 ng/μL), together with 3 mg/ml fluorescein-labeled dextran (10,000 MW, Thermo Fisher Scientific) and 12.5% glycerol, were injected into amphioxus unfertilized eggs or one blastomere of 2-cell embryos following steps described in previous reports [72].

### Luciferase activity assays

Regulatory sequences of *Lhx3* (a 500-bp fragment upstream of its transcriptional start) and *Hex* (a 554-bp fragment upstream of the transcriptional start) genes were determined according to the ATAC-seq data of *B. lanceolatum* [79]. They were respectively inserted upstream of the firefly luciferase gene in pGL3 (Promega) and modified by PCR to generate mutant versions (Additional file 2: Table S3). *Pitx* binding sites in the regulatory sequences of *Lhx3* and *Hex* were predicted using JASPAR profiles at http://jaspar.genereg.net/. Injection solutions were prepared to contain 60 ng/μL of *Lhx3* or *Hex* constructs, or *Renilla* luciferase vector pRL-SV40 (control), and 3 mg/ml fluorescein-labeled dextran (10,000 MW, Thermo Fisher Scientific), 12.5% glycerol, with or without 100 ng/μL *Pitxc* mRNA. Embryos were injected at unfertilized egg stage [72] and assayed at N4 stage (eight somites, 60 embryos for each experiment); uninjected embryos from the same batch were used as the negative control. Firefly luciferase expression levels from pGL3 and *Renilla* luciferase from pRL-SV40 were detected with the Dual Luciferase Kit (Promega) using a GloMax luminometer with an integration of 15 s, and the level of firefly luciferase was normalized to *Renilla* luciferase activity for each experiment.

### Pharmacological treatments

SB505124 (Sigma), dorsomorphin homolog-1 (DMH1, Sigma), DAPT (Calbiochem), SU5402 (Calbiochem), and BMS593 (Sigma) were used respectively to inhibit Nodal, BMP, NOTCH, FGF, and RA signaling pathway. They were dissolved in DMSO following standard protocols and used at the following concentrations: 50 μM for SB505124, 40 μM for DMH1, 150 μM for DAPT, 50 μM for SU5402, and 1 μM for BMS593. SB505124 treatment was conducted at N0 (neurula with zero somite), 8S, 10S, T0, and T1 stages; DMH1, DAPT, SU5402, and BMS593 treatments were all conducted at N0 stage. For each treatment, an equal volume of DMSO was applied to the same batch of embryos and used as a negative control. All treatments were performed in six-well plates containing 3 mL of filtered seawater and 200–300 embryos. The treated embryos were fixed at required stages for in situ hybridization or cultured continuously for morphological observation.

## Supplementary Information

**Additional file 1: Figure S1.** Location of *Pitxa/c* positive cells. **Figure S2.** Mutation rates of *Pitx* TALEN1, TALEN2 and TALEN3 in F0 embryos. (**Figure S3, S4 and S5**) Genotype analysis of *Pitx* TALEN1, TALEN2 or TALEN3 mutants. **Figure S6.** The expression pattern of *Dand5-Nodal-Lefty-Pitx* in *Pitx* mutants. **Figure S7.** The expression pattern of *Pax2/5/8* in *Pitx* mutants. **Figure S8.** The dependence of *Pitx* gene on Nodal signaling. (**Figure S9 and S10**). Genotype analysis of *Pitxa/c* homozygotes carrying mutations at TALEN1 and TALEN2 or TALEN3. (**Figure S11, S12 and S13**)-Expression pattern of *Pitx* and asymmetrical pharyngeal organ markers in wildtype embryos and *Pitx* mutants. **Table S1.** DNA sequence upstream of start codon of *Lhx3* and *Hex* gene. **Table S2.** *Pitxc* sequence. **Figure S14**-Expression of *Pitxa/b* and *Pitxc* in *Pitx*c mutants. **Figure S15.** The dependence of *Pitx* gene on BMP signaling after N1 stage. (**Figure S16 and S17**) Studies for identifying the X factor.

**Additional file 2: Table S1.** Primers used for amplifying gene fragments including TALEN target sites and the amplicon sequences. **Table S2.** Primers used for making pGEM-T-Easy constructs. **Table S3.** Primers used for cloning and mutagenesis of *Lhx3* and *Hex* promoter sequence.

**Additional file 3.** Uncrooped gel images. Uncropped gel images shown in Additional file 1 figures.

## Data Availability

All relevant data are within the paper and its Supporting Information.
