# Supplementary material for: Pitx controls amphioxus asymmetric morphogenesis by promoting left-side development and repressing right-side formation
Source: BMC Biol. 2021 Aug 20;19:166. doi: 10.1186/s12915-021-01095-0 (PMC8377849; doi:10.1186/s12915-021-01095-0)
Supplement: Supplementary file 1 — Additional file 1: Figure S1. Location of Pitxa/c positive cells. Figure S2. Mutation rates of Pitx TALEN1, TALEN2 and TALEN3 in F0 embryos. (Figure S3, S4 and S5) Genotype analysis of Pitx TALEN1, TALEN2 or TALEN3 mutants. Figure S6. The expression pattern of Dand5-Nodal-Lefty-Pitx in Pitx mutants. Figure S7. The expression pattern of Pax2/5/8 in Pitx mutants. Figure S8. The dependence of Pitx gene on Nodal signaling. (Figure S9 and S10). Genotype analysis of Pitxa/c homozygotes carrying mutations at TALEN1 and TALEN2 or TALEN3. (Figure S11, S12 and S13)-Expression pattern of Pitx and asymmetrical pharyngeal organ markers in wildtype embryos and Pitx mutants. Table S1. DNA sequence upstream of start codon of Lhx3 and Hex gene. Table S2. Pitxc sequence. Figure S14-Expression of Pitxa/b and Pitxc in Pitxc mutants. Figure S15. The dependence of Pitx gene on BMP signaling after N1 stage. (Figure S16 and S17) Studies for identifying the X factor. [file 12915_2021_1095_MOESM1_ESM.docx]

**Additional file1**

***Pitx* controls amphioxus asymmetric morphogenesis by promoting left-side development and repressing right-side formation**

**SECTION 1: Location of *Pitxa/c* positive cells.**

**SECTION 2: Mutation rates of *Pitx* TALEN1, TALEN2 and TALEN3 in F0 embryos.**

**SECTION 3: Genotype analysis of *Pitx* TALEN1, TALEN2 or TALEN3 mutants.**

**SECTION 4: The expression pattern of *Dand5-Nodal-Lefty-Pitx* in *Pitx* mutants.**

**SECTION 5: The expression pattern of *Pax2/5/8* in *Pitx* mutants.**

**SECTION 6: The dependence of *Pitx* gene on Nodal signaling.**

**SECTION 7: Genotype analysis of** ***Pitxa/c* homozygotes carrying mutations at TALEN1 and TALEN2 or TALEN3.**

**SECTION 8:** **Expression pattern of *Pitx* and asymmetrical pharyngeal organ markers in wildtype embryos and *Pitx* mutants.**

**SECTION 9: DNA sequence upstream of start codon of *Lhx3* and *Hex* gene.**

**SECTION 10: DNA sequence of *Pitxc*.**

**SECTION 11: Expression of *Pitxa/b* and *Pitxc* in** ***Pitx*c mutants.**

**SECTION 12: The dependence of *Pitx* gene on BMP signaling after N1 stage.**

**SECTION 13: Studies for identifying the X factor.**

**SECTION 1: Location of *Pitxa/c* positive cells.**

**
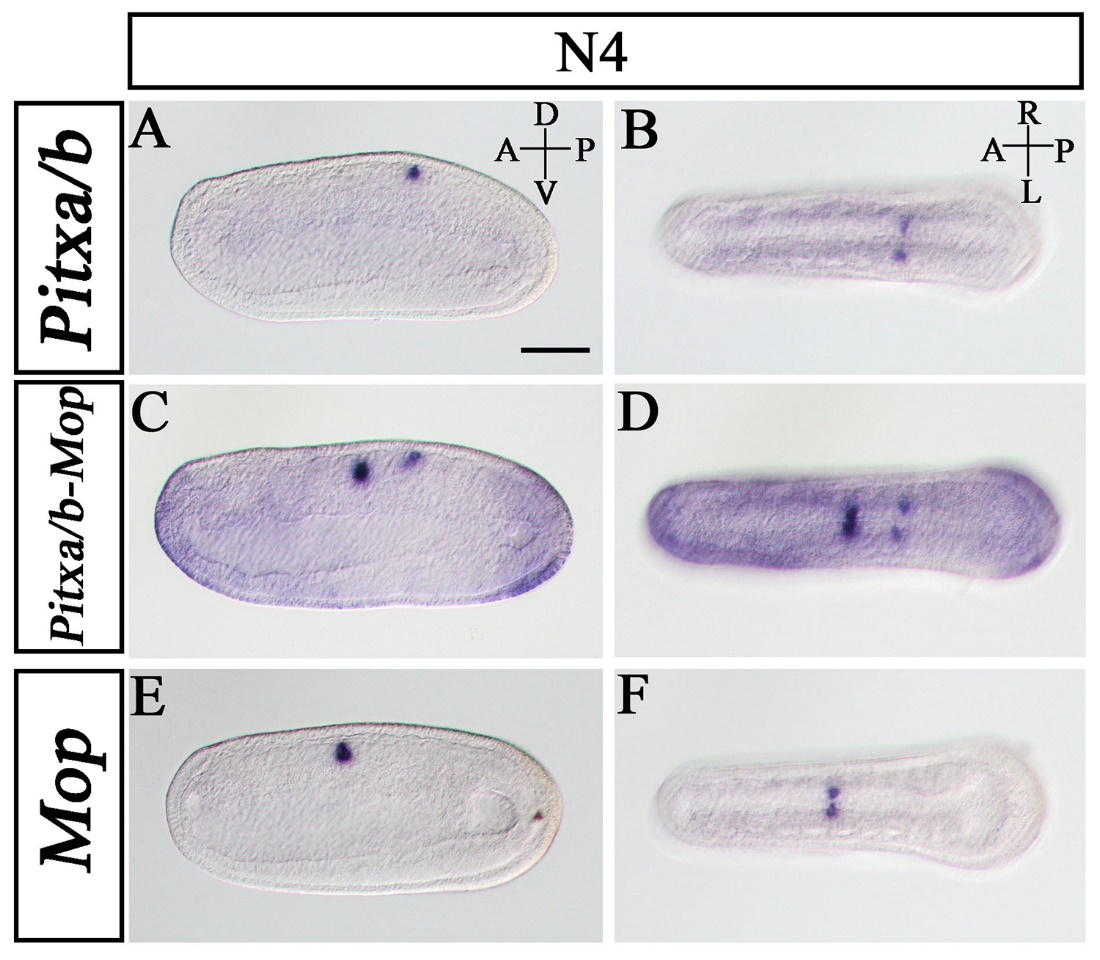
**

**Fig. S1** (a-f) *Pitxa/b* is expressed more posteriorly than the dorsal Hesse organ marked by *Mop* through double in situ hybridization at N4 stage, as shown in lateral (a, c, e) and dorsal (b, d, f) views. A, anterior; P, posterior; D, dorsal; V, ventral; L, left side; R; right side. Scale bars: 50 μm.

**SECTION 2: Mutation rates of *Pitx* TALEN1, TALEN2 and TALEN3 in F0 embryos.**

**
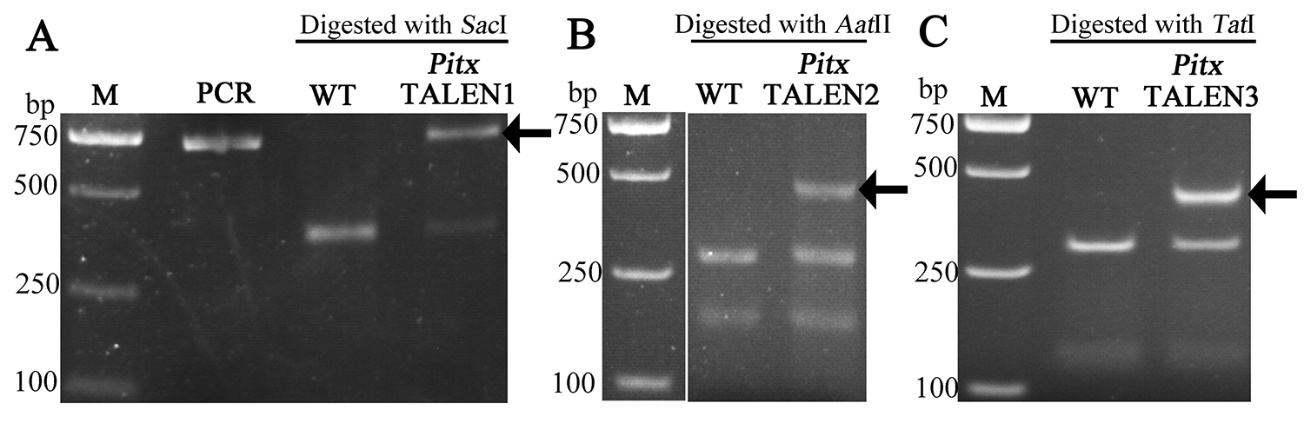
**

**Fig. S2** Around 15 wild type (WT) embryos or embryos injected with TALEN mRNA are randomly collected at T1 stage, for DNA extraction and PCR amplification. PCR products from wild type and injected embryos are both digested with *Sac*I for *Pitx* TALEN1(left hand gel), *Aat*II for *Pitx* TALEN2 (middle gel), *Tat*I for *Pitx* TALEN3 (right hand gel). Arrows indicate the PCR products from the TALEN mRNA injected embryos are uncut. The mutation rate of *Pitx* TALEN1 mRNA injected embryos is approximately 50%, *Pitx* TALEN2 mRNA injected embryos is approximately 30% and *Pitx* TALEN3 mRNA injected embryos is approximately 40%. M, DNA size marker; PCR, undigested PCR product control.

**SECTION 3: Genotype analysis of *Pitx* TALEN1, TALEN2 or TALEN3 mutants.**

**
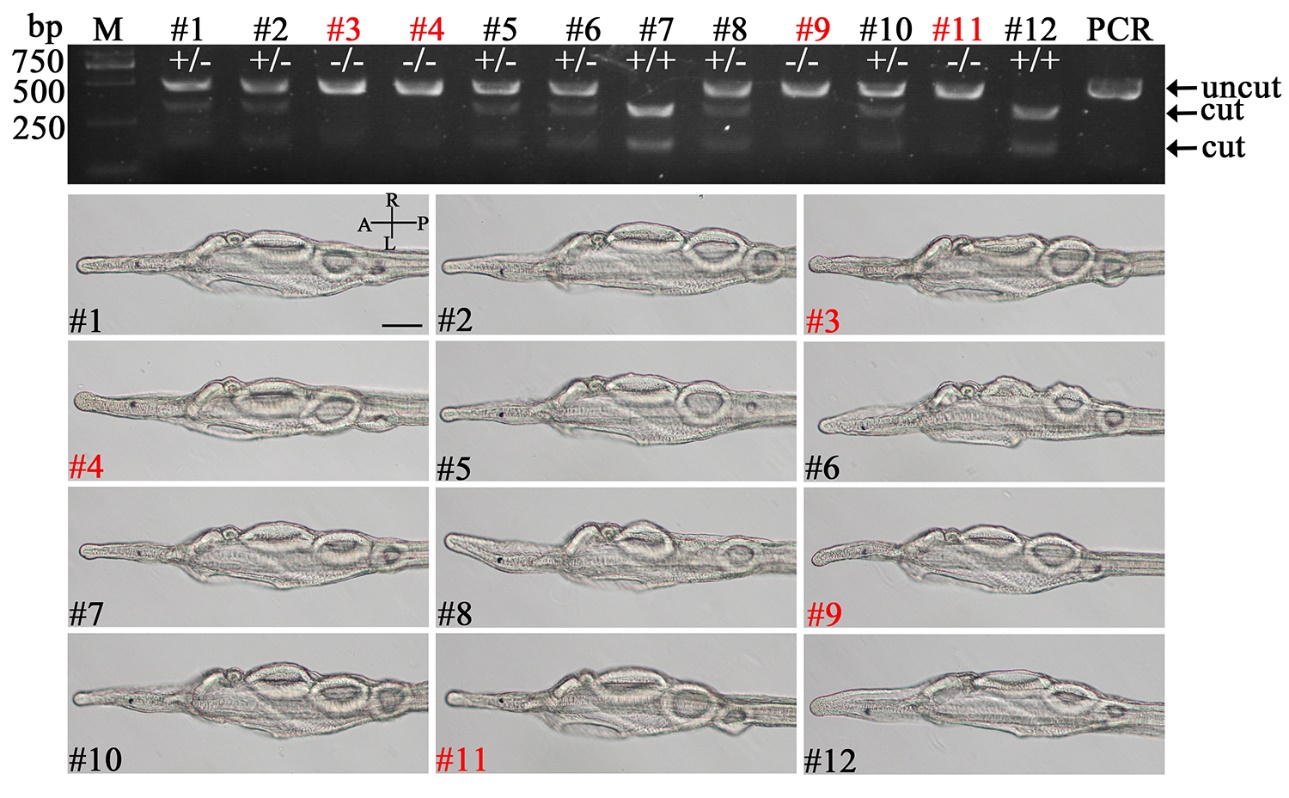
**

**Fig. S3** Genotype analysis of *Pitx* TALEN1 mutants. Gel electrophoresis of *Sac*I digested PCR products containing the TALEN1 target site amplified from *Pitx* TALEN1 F2 larva genomic DNA. The genotype of #1-#12 individuals corresponds to the number #1-#12 in this photo. Individuals of number #3, #4, #9 and #11 are *Pitx* TALEN1^-/-^ mutants and the numbers are marked in red. M, DNA size marker; PCR, undigested PCR product control. A, anterior; P, posterior; L, left side; R; right side. Scale bars: 50 μm.


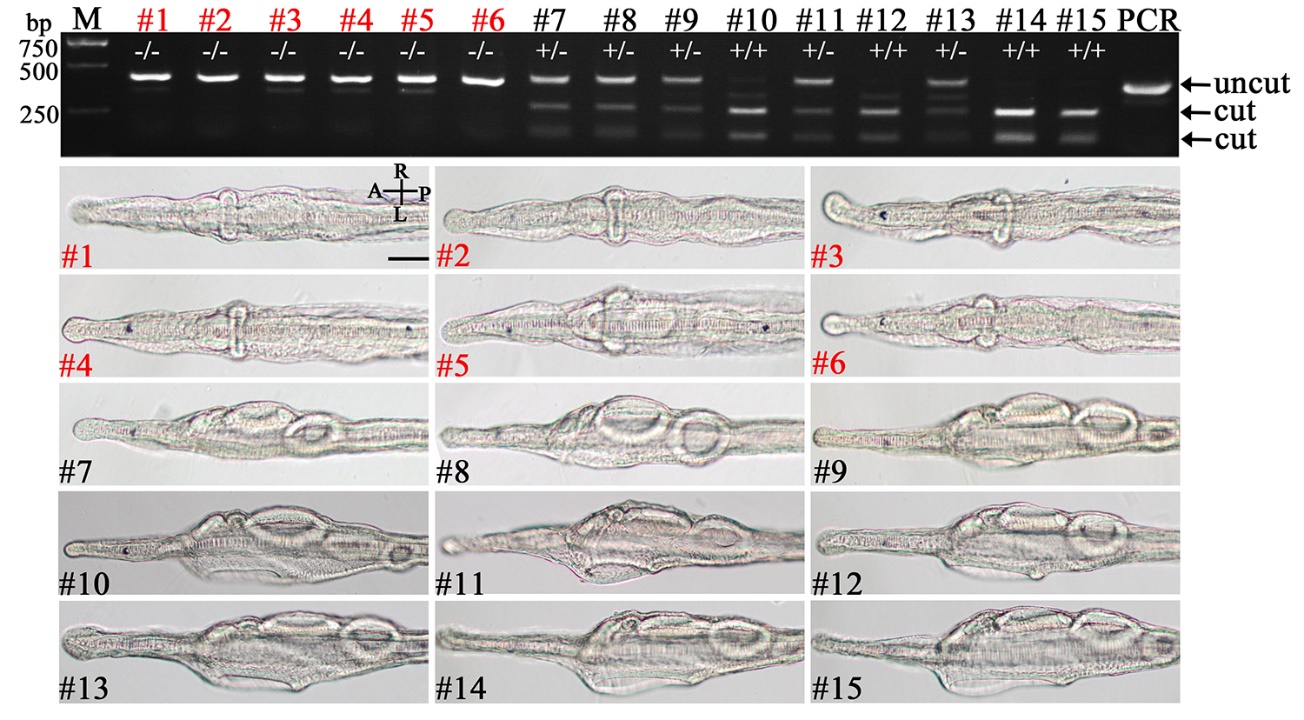


**Fig. S4** Genotype analysis of *Pitx* TALEN2 mutants. Gel electrophoresis of AatII digested PCR products containing the TALEN2target site amplified from *Pitx* TALEN2 larvae genomic DNA. The genotype below #1-#15 individuals corresponds to the number #1-#15 in this photo. (#1-#6) individuals with bilaterally symmetric phenotype are marked in red, (#7-#8) individuals with no mouth phenotype and (#9-#15) individuals with normal phenotype. M, DNA size marker; PCR, undigested PCR product control. A, anterior; P, posterior; L, left side; R; right side. Scale bars: 50 μm.


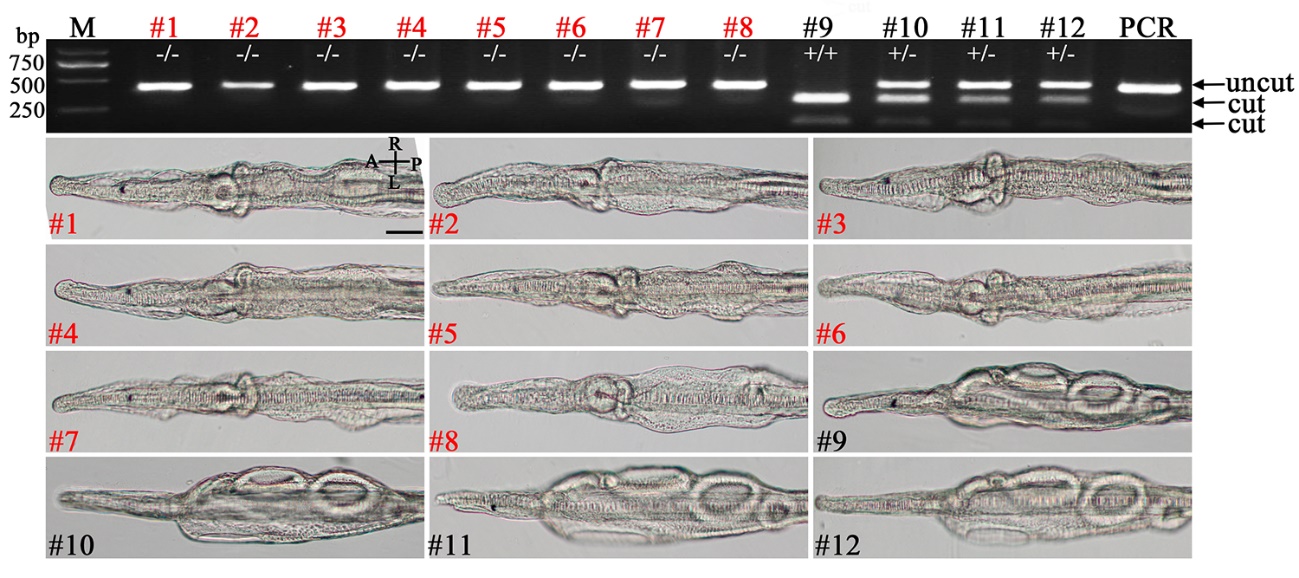


**Fig. S5** Genotype analysis of *Pitx* TALEN3 mutants. Gel electrophoresis of *Tat*I digested PCR products containing the TALEN3 target site amplified from *Pitx* TALEN3 F2 larvae genomic DNA. The genotype below #1-#12 individuals corresponds to the number #1-#12 in this photo. (#1-#8) individuals with bilaterally symmetric phenotype are marked in red, ((#9-#12) individuals with normal phenotype. M, DNA size marker; PCR, undigested PCR product control. A, anterior; P, posterior; L, left side; R; right side. Scale bars: 50 μm.

**SECTION 4: The expression pattern of *Dand5-Nodal-Lefty-Pitx* in *Pitx* mutants.**

**
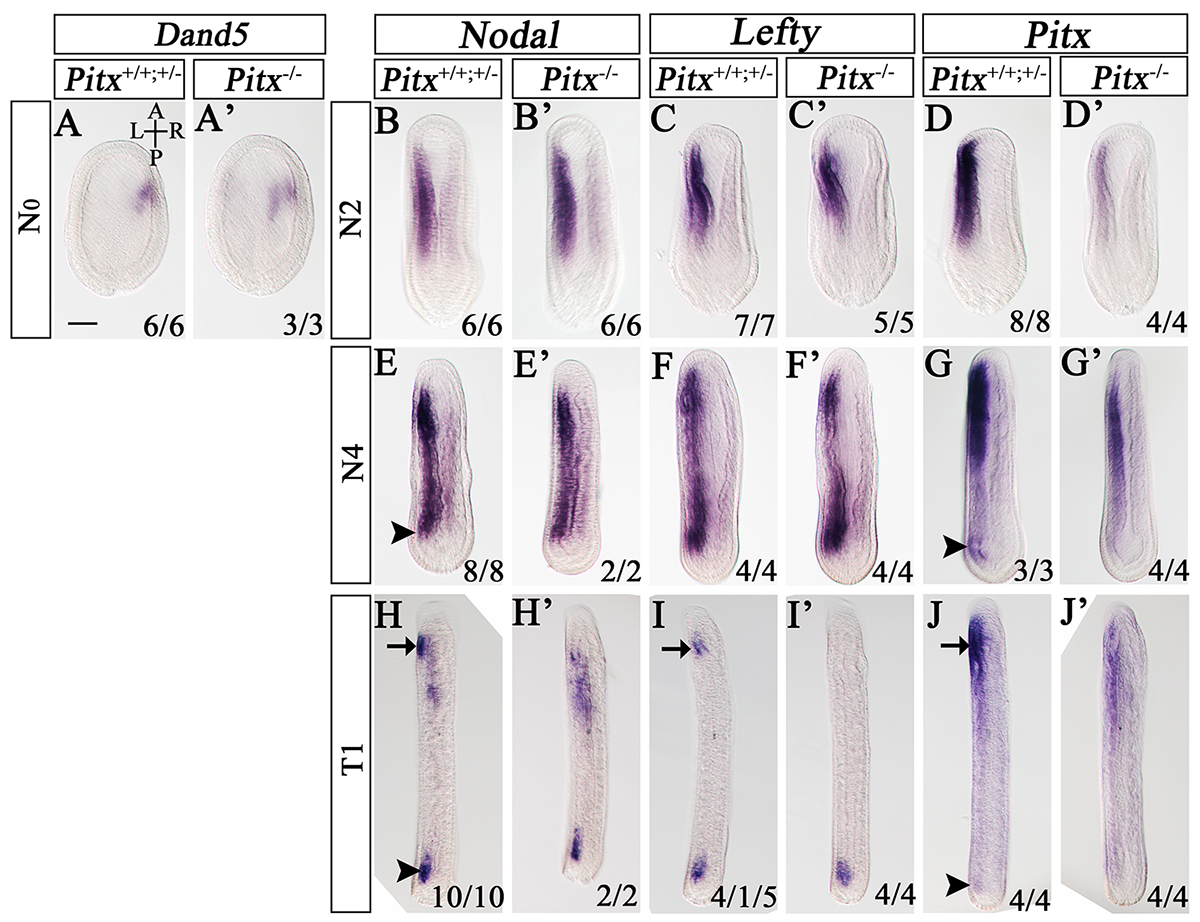
**

**Fig. S6** (a-j’) These embryos are obseved at N0 stage (neurula with zero somites) (a, a’), N2 stage (neurula with 4⁓5 somites) (B-D, B’-D), N4 stage (e-g, e’-g’) and T1 (h-j, h’-j’). Arrowheads indicate the expression of *Nodal* (e, h) and *Pitx* (g, j) on the left tail bud in *Pitx^+/+; +/-^* embryos. Arrows show the expression of *Nodal*, *Lefty* and *Pitx* expression in the forming preoral pit (h, i), which is disappeared in *Pitx^-/-^* mutants (h’, i’). Numbers in the bottom right corner of a panel show the number of times the phenotype was seen, out of the total number of embryos from that genotype analysed. All images are dorsal views. A, anterior; P, posterior; L, left side; R; right side. Scale bars: 50μm.

**SECTION 5: The expression pattern of *Pax2/5/8* in *Pitx* mutants.**


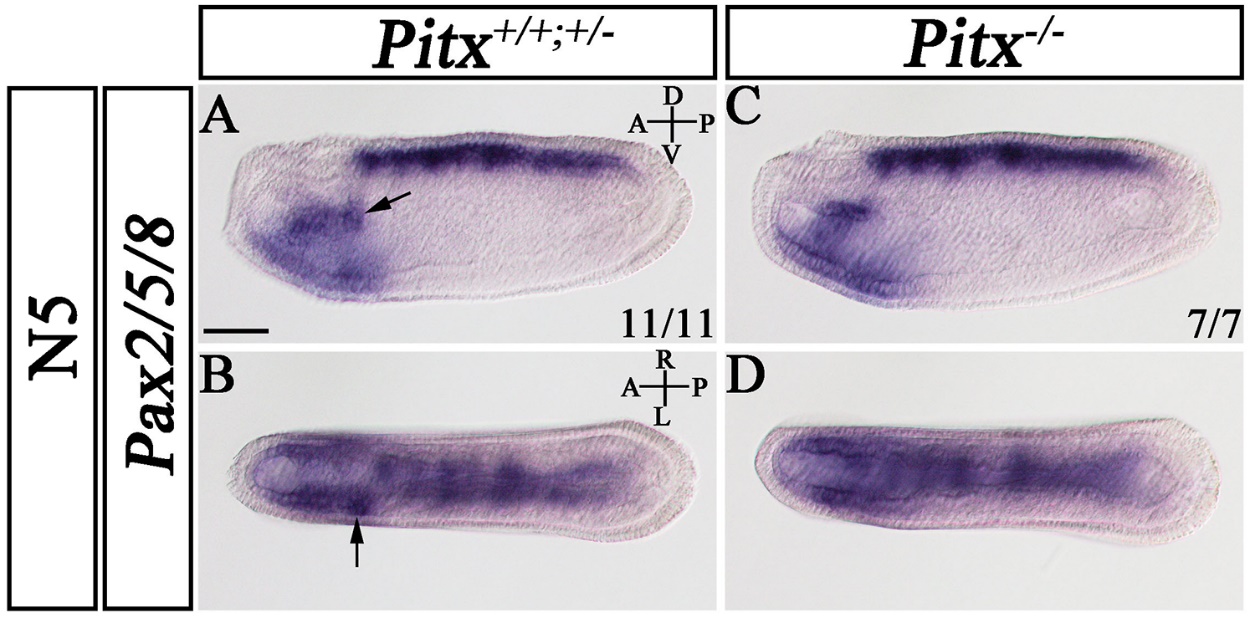


**Fig. S7** (a-d) Expression of *Pax2/5/8* in the Hatschek’s nephridium (arrows) is shown in *Pitx*^+/+; +/-^ embryos (a, b) and lost in *Pitx^-/-^* mutants (c, d). The embryos in (a, c) are lateral views, in (b, d) are dorsal views. All embryos are at N5 stage. Numbers in the bottom right corner of a panel show the number of times the phenotype was seen, out of the total number of embryos from that genotype analysed. A, anterior; P, posterior; D, dorsal; V, ventral; L, left side; R; right side. Scale bars: 50μm.

**SECTION 6: The dependence of *Pitx* gene on Nodal signaling.**

**
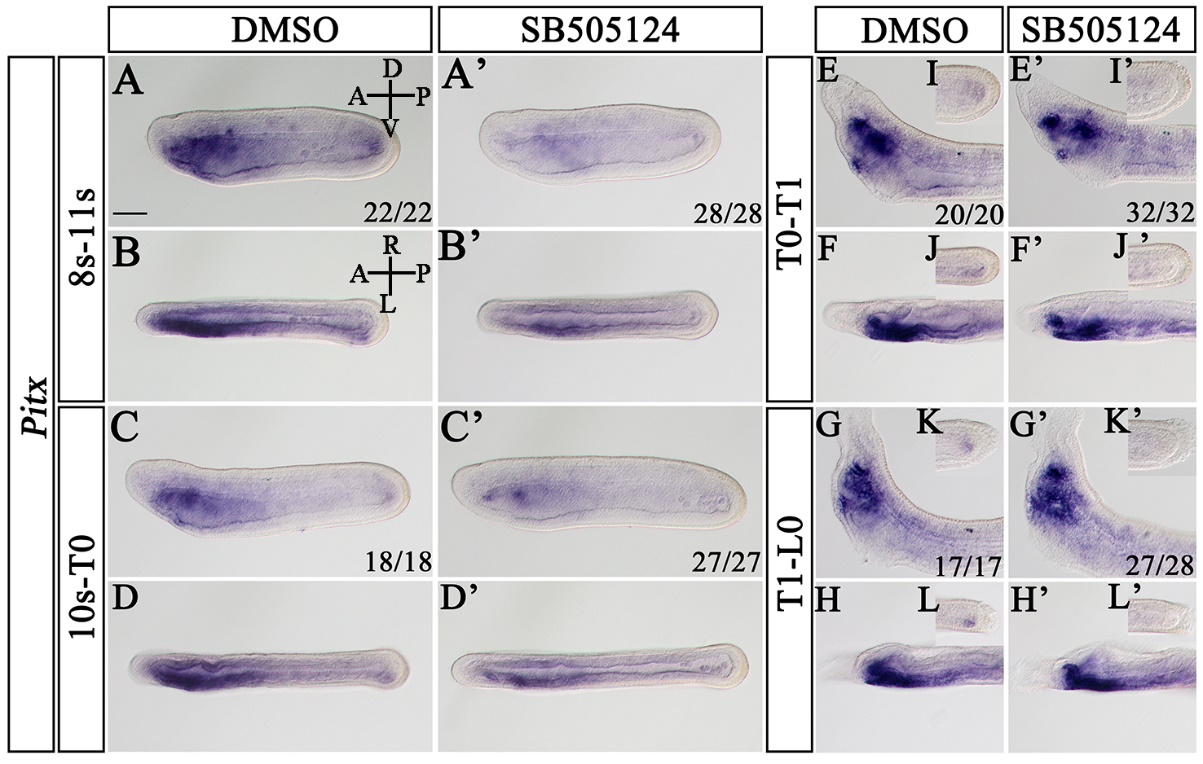
**

**Fig. S8** (a-d’) Compared to expression in control embryos (treated with DMSO) (a, b, c, d), *Pitx* expression is decreased in embryos treated with SB505124 from 8S to 11S stage (a’, b’) and from 10S to T0 stage (c’, d’). (e-h’) *Pitx* expression is detected in the anterior endoderm in control embryos treated from T0 to T1 (e, f) stages and from T1 to L0 stages (g, h), but weakly decreased in embryos treated with SB505124 (e’, f’, g’, h’). (i-l’) The expression of *Pitx* in left tail (i, j, k, l) is eliminated in embryos treated with SB505124 (i’, j’, k’, l’). Images in (a, a’, c, c’, e, e’, g, g’, i, i’, k, k’) are left lateral views, (b, b’, d, d’, f, f’, h, h’, j, j’, l, l’) are dorsal views. Numbers in the bottom right comer of a panel show the number of times in phenotype was observed in total number of embryos examined. A, anterior; P, posterior; D, dorsal; V, ventral; L, left side; R; right side. Scale bars: 50 μm.

**SECTION 7: Genotype analysis of *Pitxa/c* homozygotes carrying mutations at TALEN1 and TALEN2 or TALEN3.**

**
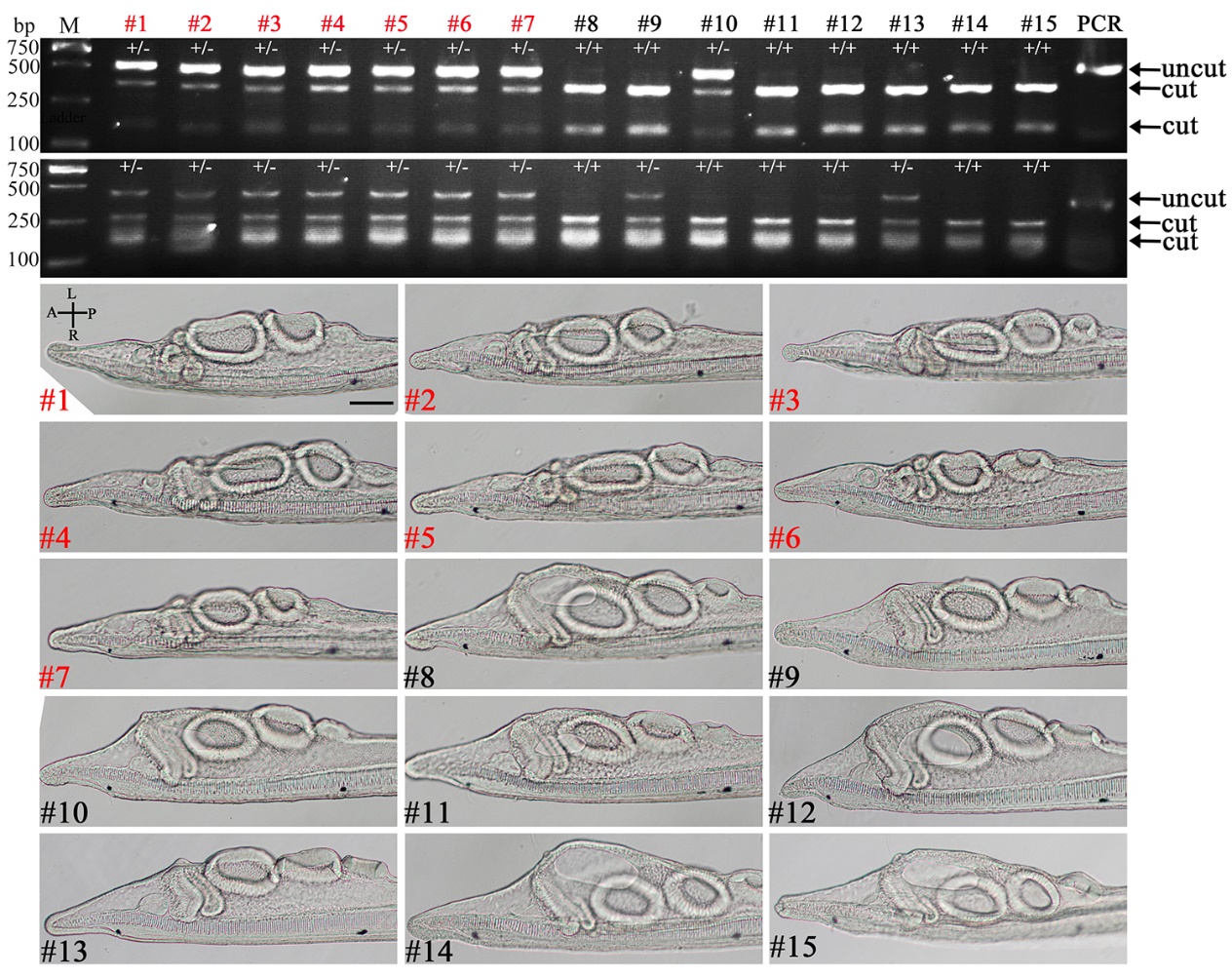
**

**Fig. S9** Genotype analysis of *Pitxa/c* homozygotes carrying mutations at TALEN1 and TALEN2. Gel electrophoresis of *Sac*I or *Aat*II digested PCR products containing *Pitx* TALEN1 target site (the first line of gel) or *Pitx* TALEN2 target site (the second line of gel), respectively, amplified from embryos with normal or abnormal phenotype. The genotype below #1-#15 individuals is corresponding to the number #1-#15 in gel electrophoresis (both first line and second line). Individuals of number (#1-#7) are *Pitxa/c* homozygotes and the numbers are marked red. M, DNA size marker; PCR, undigested PCR product control. Scale bars: 50 μm.


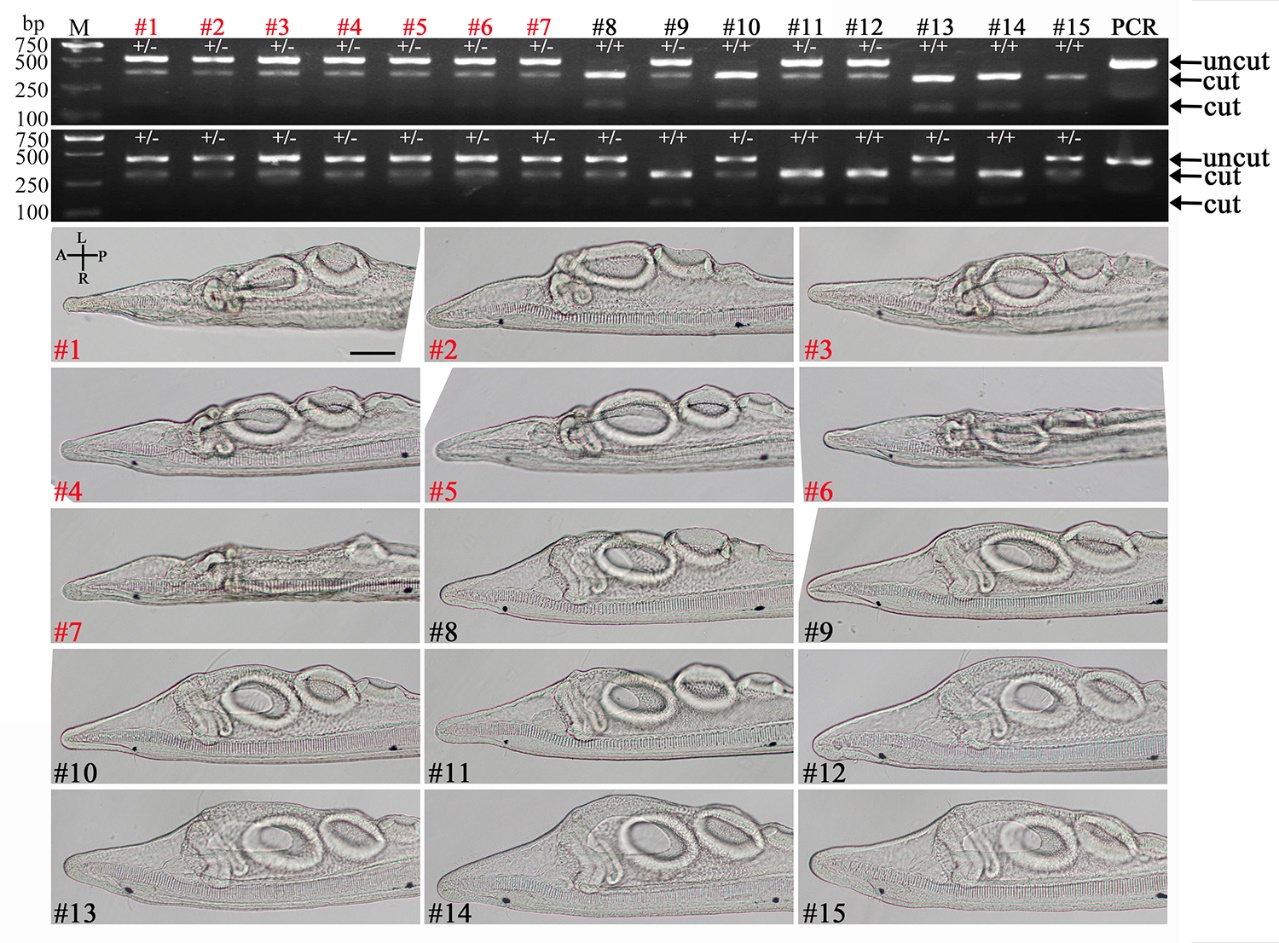


**Fig. S10** Genotype analysis of *Pitxa/c* homozygotes carrying mutations at TALEN1 and TALEN3. Gel electrophoresis of *Sac*I or *Tat*I digested PCR products containing *Pitx* TALEN1 target site (the first line of gel) or *Pitx* TALEN3 target site (the second line of gel), respectively, amplified from embryos with normal or abnormal phenotype. The genotype below #1-#15 individuals corresponds to the number #1-#15 in gel electrophoresis (both first line and second line). Individuals of number (#1-#7) are *Pitxa/c* homozygotes. ((#8-#15) individuals with normal phenotype and the numbers are marked red. M, DNA size marker; PCR, undigested PCR product control. Scale bars :50 μm.

**SECTION 8: Expression pattern of *Pitx* and asymmetrical pharyngeal organ markers in wildtype embryos and *Pitx* mutants.**

**
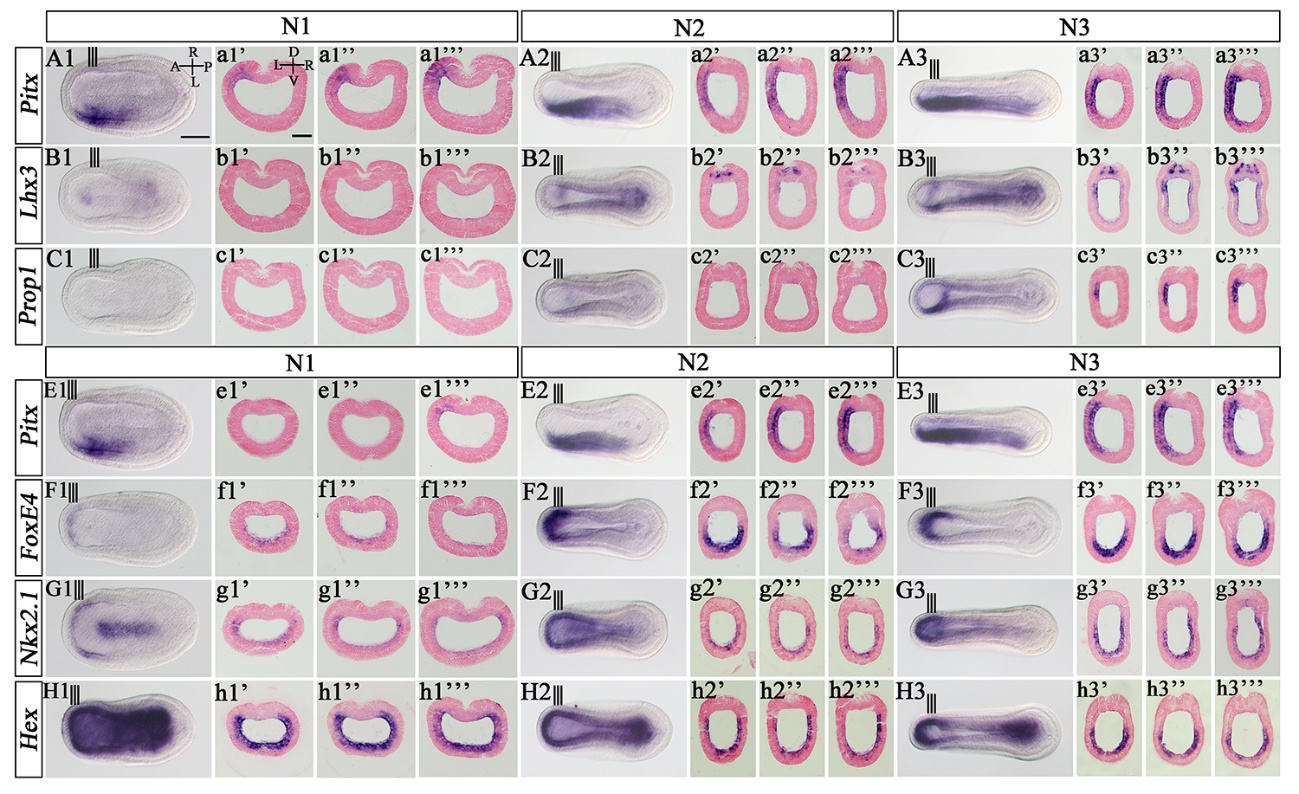
**

**Fig.** **11** Expression pattern of *Pitx* and asymmetrical pharyngeal organ markers. (a1-c3, e1-e3) *Pitx* is expressed in the left anterior-dorsal half of embryos from N1 neurula to N3 neurula stage (a1-a3, e1-e3) and *Lhx3*, *Prop1* is activated in the prospective preoral pit region from N2 neurula to N3 neurula stage (b1-b3, c1-c3) as shown by WISH and transvers sections in (a1’- c3’’’). (f1-h3) The expression of right-sided organ maker, *FoxE4* (f1-f3), *Nkx2.1* (g1-g3), *Hex* (h1-h3) from N1 neurula to N3 neurula stage, as shown by WISH and transvers sections in (e1’-h3’’’). The lines in (a1-h3) indicate the position of transverse sections of the embryos. The embryos detected by WISH are all in dorsal views. A, anterior; P, posterior; D, dorsal; V, ventral; L, left side; R; right side. Scale bars: 50 μm.


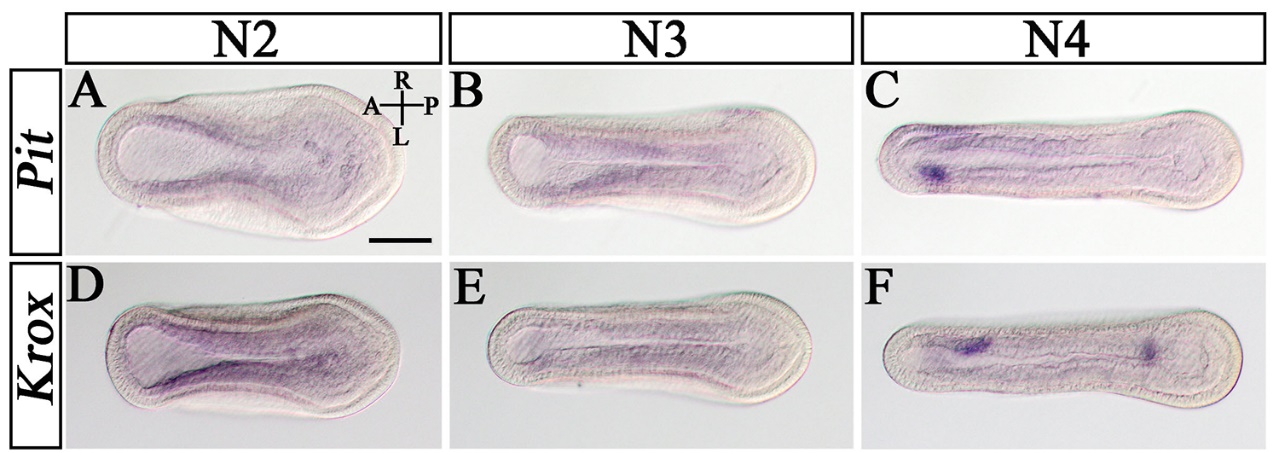


**Fig. S12** Expression pattern of *Pit* and *Krox* at neurula stage. (a-f) The expression of *Pit* and *Krox* are both not initiated at N2 stage (a, d) and N3 stage (b, e) until N4 stage (c, f). All images are dorsal views. A, anterior; P, posterior; L, left side; R; right side. Scale bars are 50 μm.


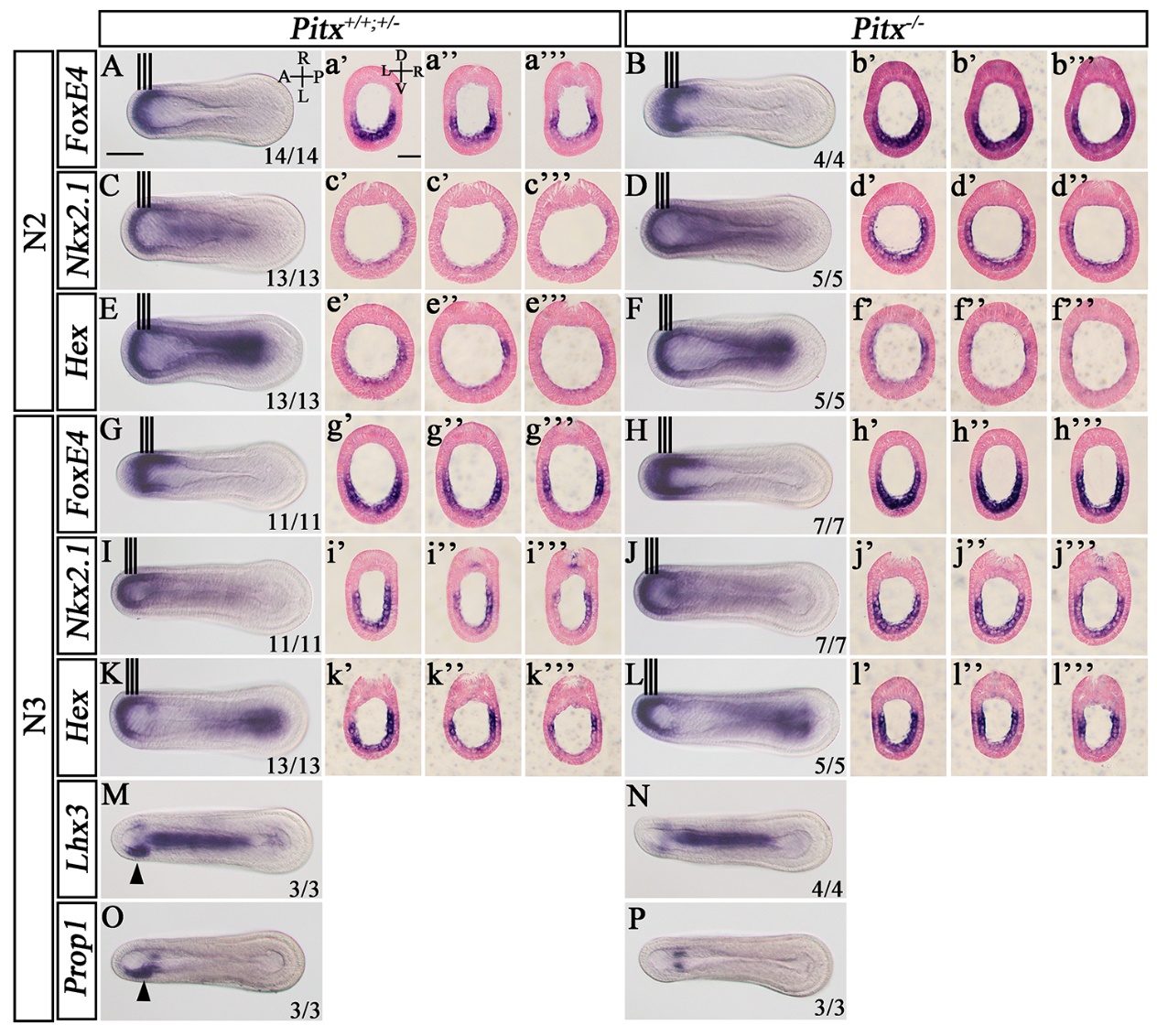


**Fig. S13** Expression pattern of pharyngeal organs markers in *Pitx* mutants at early neurula stage. **(**a-f, a’-f’’’**)** *FoxE4* is expressed in the CSG (a, b), *Nkx2.1* (c, d) and *Hex* (e, f) are expressed in the endostyle and transversal sections are shown in (a’-a’’’, c’-c’’’, e’-e’’’) for *Pitx*^+/+; +/-^ embryos and in (b’-b’’’, d’-d’’’, f’-f’’’) for *Pitx*^-/-^ embryos at N2 stage. (g-l, g’-l’’’) At N3 stage, the expression pattern of *FoxE4* (g, h), *Nkx2.1* (i, j) and *Hex* (k, l) is maintained as N2 stage, and corresponding transverse sections are shown in (g’-g’’’, I’-i’’’, k’-k’’’) for *Pitx*^+/+; +/-^ embryos and in (h’-h’’’, j’-j’’’, l’-l’’’) for *Pitx*^-/-^ embryos. (m-p) *Lhx3* and *Prop1* expression are activated in the prospect preoral pit at N3 stage (m, o), while the expression in the preoral pit is lost in *Pitx*^-/-^ mutants (n, p). The lines in (a-l) indicate the position of transverse sections of the embryos. The embryos used for WISH are all dorsal views. Numbers in the bottom right corner of a panel show the number of times the phenotype depicted was seen, out of the total number of embryos from that genotype analysed. A, anterior; P, posterior; D, dorsal; V, ventral; L, left side; R; right side. Scale bars: 50 μm.

**SECTION 9: DNA sequence upstream of start codon of *Lhx3* and *Hex* gene.**

| **Bf *Lhx3* promoter** |
| --- |
| CTGGTGATGATCTAATTTCATATCAGTACATGACTTTCTGACAGACCACCTTGGAACTGTGACCTCAGGGCCTAAGCACTGTCTTCCACCACAGCATTAAGTAGGCCATCTGTAGAACCTGTGTTACACCTGCTCGTGTGCAGTCCCTCTGTGCAATCTTCGCTCAACACTTGCATAACAACAGGTAGAACAAGAGCGGGGTATCAGCAGCAGTACGTAAACAATGCAGCGGCCCCGCCTGTTGTCCCCTGCGGCGGCAGCTTCCAGAAAGGGGCTTTGTTTAGCTTTTCTTGCCCTGTGATTGGTGGAGCTCGGCGCAGAGGCATCGCGGTCTGCTTTAGATGCATGCTACTAAACAAGTAGCTAAACACGCGAGCGCACGGCTCTGATTAGTAGTCCCGGGACTTCTGTAGGACCTGTCCTCCCCAACTCGCCAGCTGGATACAACAATAGACTTCTATCTGGTGTGGAACAGTGCTAGCTGCAACAAGCAGAGCAAAATG |
| **Bf *Hex* promoter** |
| GTTTTACGTCTGATTTTTCTTCAGAGGTTGTGTGCTTAATATCACTTCACGTAAACTTACGACTTTCTAAAAAGGGTTGTATAGGTGTATGCAAGCTTAGTATGCATTAACCATAGCATACTTACGTTATGAATATGCATTTCGTATGTCTGGTGTACTAAGTAGTGTTGCCGTTACCCAAATGGCCACCAGACGACTAGAATAATGTATAATAATGCGTGTACGAATTGTCCCATTGTTTTGGAGCCAGGCGTCGAAACACACGGGGTGCCCCCCATGCACAAAAAATGGGCGGAGTTACCGAGAGCGAGCATGTTTGCACAACTCGCCAATCACCTCTTGACCAATCAGAGGGCTTGTTGAGAGTAAAGTGATTAGTGGCTATTGTTCTCGAGTGAAATTAATTGATATTGATTGGCCCCGCCTCCGATGGGGTTGAGTTATGTACTTATTGGGACGTGAGGTCAGTGTTCTCTACGACTTCGTACAGTCAAGATCGTCTCTCTTGTGTTTATTACTTGCAGTACGAGCCCGAACGTTACGTACCGAGGAAGATG |

**Table S1:** Red ATG = start codon; blue text = 5’ untranslated region; blue highlight = putative *Pitx* binding site; underlined blue highlight = binding sites chosen for mutagenesis

**SECTION 10: DNA sequence of *Pitxc***

| **GCTAAGCGACCATAAGCCGATTACAAACATATCTAAGGAGGACATCGTGCAGTGCGAGTATGTCGTTCCCGTCGCTTCGTCGTTCTTGATTTATCGTTCGTCGACAGATCGGCCGCTCCGCCAGCTCGTGTCGGACGCCGTACGGGGAAAGACCTGGCATTCCTGACATCGCAGCACAGTGCACCGTTCCCATCGTTAACCGAGCTAAAAACTTAATCCATAATAATTGATGAATGGACACCTTGAACGACTCGCTGAGCTTAGAGCAGCTTGTGAGCGTAAGCCACGGAGAAGCCAACTGACTATGGCCGGGATGGCGCCGATCAACAACAGTGGCACAAGTACGGGCAGCGCATTGGACAGCACGGCGGTAGCGGGAGCTCACACCAGCATGGCCGGGACAGATAGCAGCATGGACTCCACTCACGGCTCCGGAGgtacagaccaactccgtaactactgtactacgtactgctactacacgccttagccggcgtgcgacggtgcccgactcctgtaatttctgtggtttgtgcgacgttaggtctgctcgcgagcttgtcgagcgtggccccgagcacgagtaaggtgccgccgaggcccggcgagccaacctgctcctggggtgtgtagttgttaggcaaaaatcctggccctgccggtaccgcgatgagagcccgtcccctaacctcgctccaaattacccgtgtcgatcctttcaatcatgggaattttcacggcagttgtttttgttgtgccaacagttactttgtctcgagtttttatttttaatggagaagtgtacagattacaagtctgatgtgagatacaggaaagagagagaaaaagtccaatgcctttgcggtagatgtaggctgcgctttacgtggatgtgtattccgaaattggaccaagtcttcgggtgtgcgacagggcactgcgctcgggtctaaagcgttagagaggtgtagtaactgttcctacctgtctacctatctctgtgtatcacagccaaacaaatctcggacatccactggcaaaaagcccactggcctcccgcattatcttgcctaaaatgacttcttgatcctttttcaacaaacgacaagtttgcagtgctgcctgtttatatttattcttcccactttcgtcctgtcatatttatgtttccggatgtgcacttgtcgcgatcgtggcagcgcgctatcctaacccaaaaccgatttttcctaccctaatcatgactctgtttgacgttgctgaatcacaaaacatagagagcttggctcggacattgtgttgcaaatgtgtggggtcaggtccccgccgtaaggagtagatgtgaatcgtccatctgaagtggcaccccgggtaggtgtacgacggggagtgtgtgtcggaatgtcagcctagttctagcccatgtaaaccctgctaaccgccgtcttctctctctctctctctcccccctacagCAGCCACTGTGTCGGCAGGCTCGCCCGTCGGGAAGGACTCCGGTAGCGGGTCCACGCCGTCTACGCAGGACGTCACGCAAGACGACGACGAGATGCGGAAGCGACGGCGGCAGCGTCGGCAGAGGACGCACTTCACGTCGCAGCAGCTCCAGGAGCTAGAGGCCTCCTTCGCGCGGAATCGCTACCCCGACATGGCGACACGGGAGGAGATCGCCGCCTGGACCAACCTCACAGAGGCCAGAGTTAGGgtaagtcttacattacaactccattttgtaatagctttaatacagtaaaacagagtcagctatcgtatttgaagtcgtgcggttgtctcgtcatttgttcagggcgctactcgtcaatctagatcaacaagcccgctctgtaggccggagattttcctcacataaccgacagaccgcagacaggcctggaagattgatatcatattcggggaaatcacagaaaattgagagcccttactgggttagccctgttaatttttgaccccagttcgaattatccaattgccctaactcgccttccattcataaatatcagacattggacctctttccaattgttgcttaatgttttcattagacgggggatttaaaacatttttaacattcgcttcacggttcagttccaaatggtgcgtggcaaaaaagagagaagcaagttgggatagactgagcgtgcatgtactgagacttgttagtagtactggagcatgctgcaggtctccaaagccgggatagtttaccggctcagggcctggaccccgcggtttgttctcaggcaccccgccgatttgttaagacctaacatttgtcgaatttgttttactttagttctactcgccatagagatgtaacaacttcaataggccccagctcgtaaaacatgccacaaagtctttagcggggggaaagtttagcccgaatataaaggctcagatttttactcccgtggatttctgtgttttgacgaggccgcacgcctgtccctttgtcaccccgcggagtcataaatattgcttgtggtcgtaatgcagagtttgtgttcccggcctccaaagtatttatctcgccatattgctaatgagattttctgaacaattgtgttatgaaccctggaataatagagcccatcaatcgtgtgtgtgagcaggggacggcggcggacagcccatgtcgtctcaaccgcgctctgcctcaaaaccccggtttggcgatcggaatcccccacttcaaagcagagcttatcccgtaaattttactatagtcttgatcatgggggtaatatacggcgtctgataaaagtagagtagttgcttttgacatggtagttagctttattacgtattcagggtgcgtccgtggagtatggaacttgccgggggtgtggcttctggggaatcgacgggagtaaaacgctcgattgtccgattaagattgttttataaaagcagataaaagtaatttttgtcaactgttaaaacaaagcctttaggggtgattgcttgagtgtctgtggttttgtgagtccgtgcttgggctggacggcccggcagtatcagtagctgcgagctgatccctctaaatccctttccgcgcctggtcttgagaacaggctaccgtcaacaaatgtgtcaaattcagacaaattttgatgaatgacaattggaagaagtcttggcagcaccctggggctggagtactctagccacctagatgggtggattgtttggaaagctttccgcagtgtttatgttgacttacgtgcttttttttctgtgtctgttgccgacagGTCTGGTTCAAGAACCGGCGGGCCAAGTGGCGGAAGCGCGAGCGAAATCAGCTGGGAGAATTCAAGAACGGCTTCGGTCCTCACTTCAACGGGTTGATGCAACCGTTCGACGACGGACTGTACTCCGGCTACTCGCCGGCCTACAACAACTGGGCGGCGAAGGTGCCGAGCCCGCTCACCGCCAAGTCCTTCCCGTGGGGACTCAACTCCAGCGGCGTGCCCAACGTCAACCCGCTGTCGTCTCAGGCCATGTGCTTCACCCCGCCGACCACCATCGGCACCGCCACCACCATGGTGCCGAGCATGAACGTCGGCAACGGCTTGAATTCCCTGAGCAGCCTGCAGAACCCGACGGTCGCGCCGTGCCCCTACGCCTCCCCCGGGCAGCCTTACGCTTACCGTGAGCAGTGCAATTCCAGTATCGCGGCCCTGCGACTGAAGGCCAAGCAACACTCCACGTCGGTCGCGTCCAGCTTCTCCTACCCGAGCCCCGTCCGCCAACAAACCCTCTCCGCCTGCCAGTACGCCGTCGACCGGCCCGTCTGACTGTCCTTCCCGACCCCCCTACTTCATCAACCGTTCGTAGGACATTTCCCCGCAGACAGAAAAAAGACTCTGCACACATTCTCCTCTATCTACAAACTCTGAGACTATACCGCTGAATCGACTCGATTTTTCGTGGAGAAATCATGTAAATATCATCAGAGAAAACAAAGAGAATGGACTCTTTATTTAAACGTTGGCGTATGGGATTTGTTTGCTAAAGAAATGAAATGAAGACAAATTATTTTAAGTTAGAACAAAGTAATAGTTCAGTAGATACACTCCTTGTTTCACGCACTTCAAAGTTGAAGGAAATGTTCTCTGTTTTTTTTCCTGCACGGAAAAAAGGAATTCCTAAAGAGTATTCAGCTGTTCAGAGTTCGTCGATTCCAGTATTCAACATTTTATCGATGTTTTGTATCGAAAATAGAGTATAACACATAACAGATGTAGCTTGTTCTTAGATTTTATATATTTTATCACACGTATTGTAAGAGATCATTCTATTTATGTGGTGTCAGCTGTCGATATTTAAAGCACAACAATCCACGCTTTCGCTGCGTAGTTGGAGCTGTACGTTCAGTAGATGTTGTTGTTATTTTCTTTTCCCTTGTTGTTCAATGCTTATTGATTCCAGAGTGCATCCAATCGTCGCTTGTATATCTTCTATTGTGCCTACAGAAAGTAAAACTCCTTGTGTATAGATGTTCCGTATTTGTTTCGCAGAGAGACATCCTCGCTCAATGGACGAAAAAAATCTCTGTACATTAAAAGAGGGTTTAAAATCT** |
| --- |

**Table S2:** Bold red underlined ATG : alternative start codon; blue text : 5’ or 3’untranslated region; black text: introns

**SECTION 11:** **Expression of *Pitxa/b* and *Pitxc* in *Pitx*c mutants.**

**
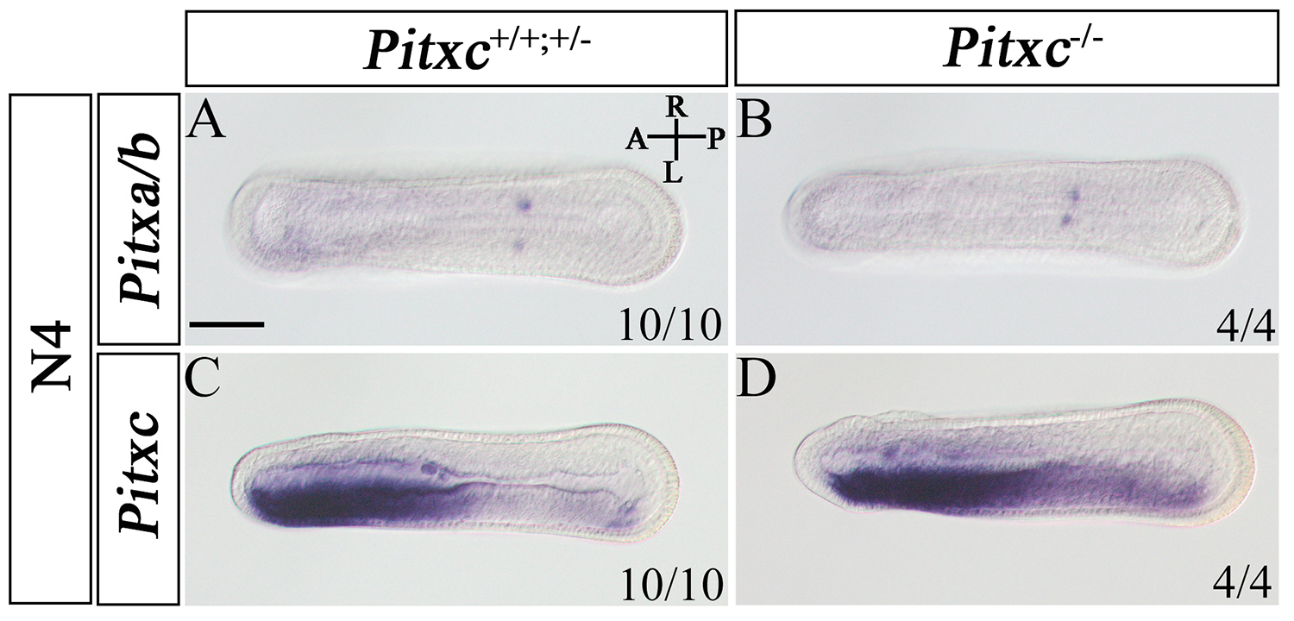
**

**Fig. S14** (a-d) Expression of *Pitx a/b* is not affected in *Pitx*c^-/-^ embryos(b), while *Pitxc* expression is slightly enhanced (d), compared with *Pitx*c^+/+; +/-^ embryos (c). Embryos are detected at N4 stage. All images are dorsal views. Numbers in the bottom right corner of a panel show the number of times the phenotype was seen, out of the number of embryos of that genotype analyzed. A, anterior; P, posterior; L, left side; R; right side. Scale bars: 50 μm.

**SECTION 12:** **The dependence of *Pitx* gene on BMP signaling after N1 stage.**

**
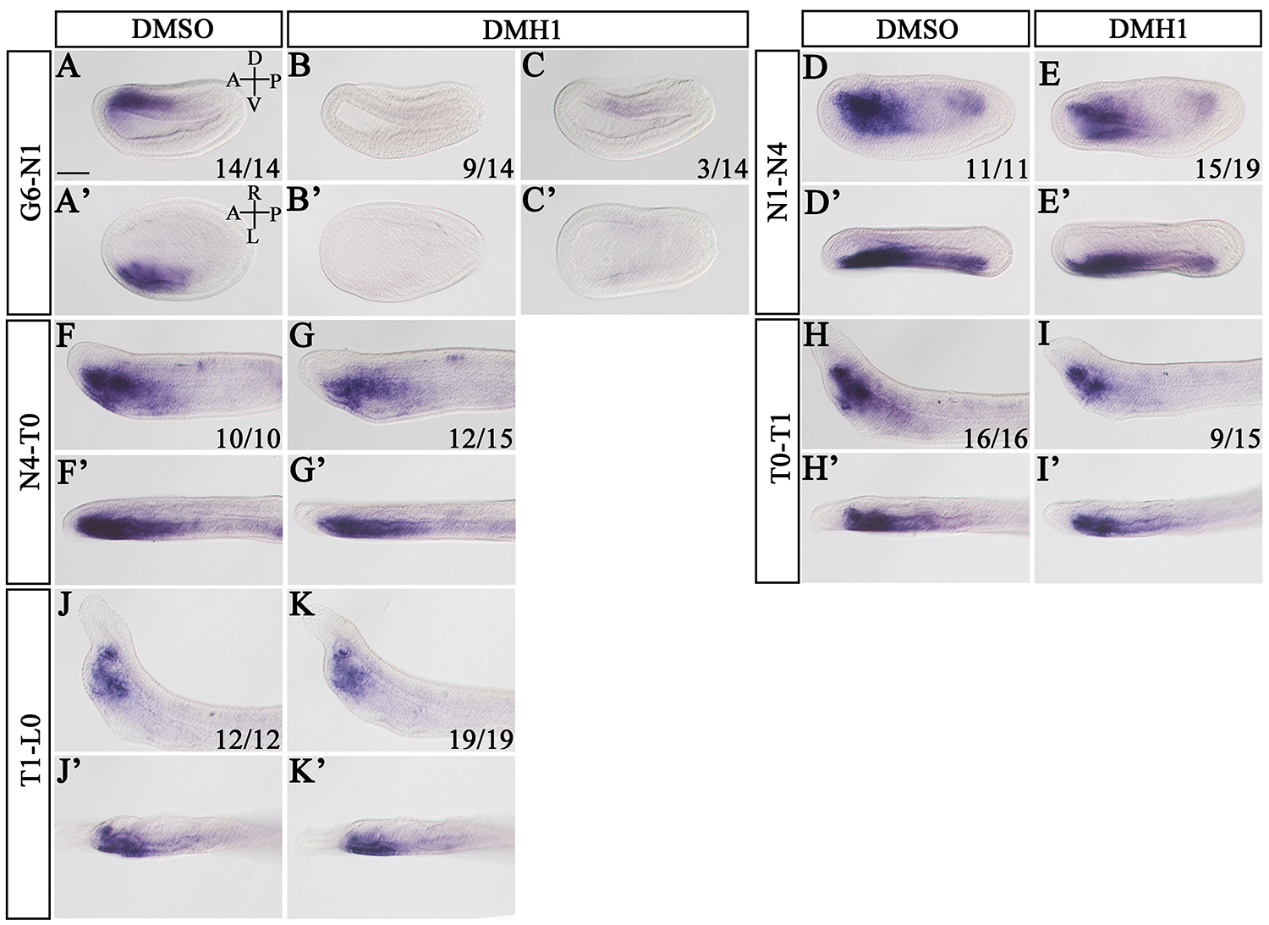
**

**Fig. S15** (a-c’) Expression of *Pitx* is undetected or transformed to a symmetrical pattern after treatment with DMH1 (inhibitor of BMP signaling) from G6 (late gastrula) to N1 stage (b, b’, c, c’). (d-g’) *Pitx* expression is reduced in embryos treated with DMH1 (inhibitor of BMP signaling) from N1 to N4 stage (e, e’), N4 to T0 stage (g, g’) or T0 to T1 (i, i’). (j-k’) The expression of *Pitx* is unaffected in embryos treated DMH1 from T1 to L0 (k, k’). Embryos in control groups are treated with DMSO. Embryos in (a-k) are shown in lateral views, in (a’-k’, l-o) are shown in dorsal views. Numbers in the bottom right corner of a panel show the number of times the phenotype was seen, the phenotype observed in total number of embryos examined. A, anterior; P, posterior; D, dorsal; V, ventral; L, left side; R; right side. Scale bars: 50 μm.

**SECTION 13:** **Studies for identifying the X factor.**

**
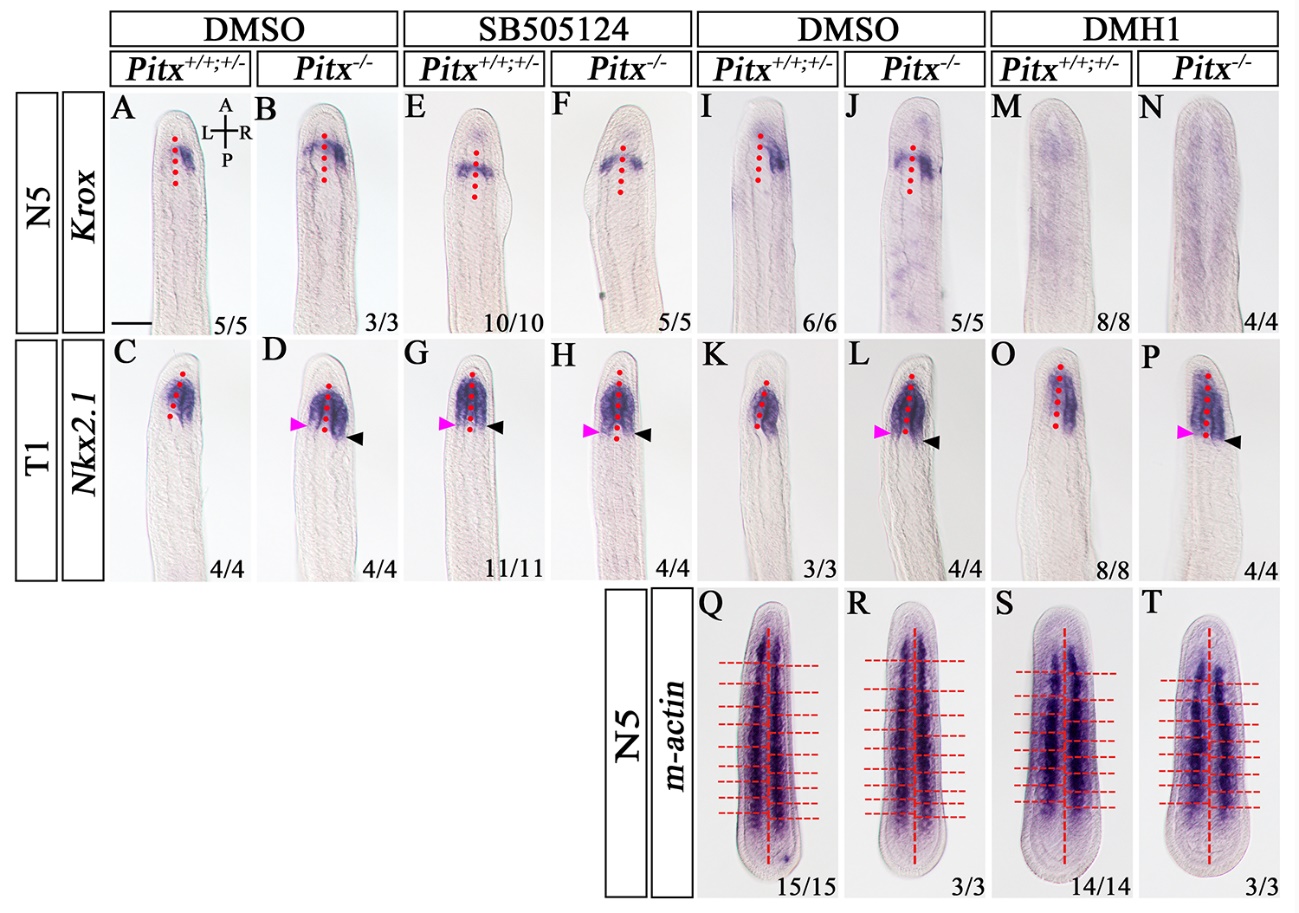
**

**Fig. S16** A non-BMP Factor X exists together with *Pitx* downstream of Nodal signaling pathway. (a-d, i-l) *Krox* expression is limited to the right-side organ CSG (a, i), *Nkx2.1* expression is in a L< R manner in the endostyle (c, k) in *Pitx*^+/+/+/-^ embryos and the expression of *Krox* (b, j) and *Nkx2.1* (d, l) is still incompletely symmetrical in *Pitx^-/-^* mutants. All the groups are treated with DMSO. (e-h) Embryos treated with SB505124, *Krox* (e, f) and *Nkx2.1* (g, h) are both L=R expressing pattern. (m-p) After inhibiting BMP signaling, the expression of *Krox* is lost (m, n) and *Nkx2.1* expression is unchanged either in *Pitx*^+/+/+/-^ embryos (o) or in *Pitx*^-/-^ embryos (p). Red and black arrowheads indicate the posterior expression border of *Nkx2.1* in endostyle on the left and right side, respectively. (q-t) Blocking BMP signaling is also not affecting the arrangement of somites either in *Pitx*^+/+; +/-^ (q, s) or *Pitx*^-/-^ embryos (r, t). Longitudinal dashed lines mark notochord, transversal dashed lines mark somite outlines. All the chemical treatment windows are from N0 to N5 stage to detect *Krox* and *m-actin* expression, and from N0 to T1 stage to detect *Nkx2.1* expression. All images are dorsal views. Numbers in the bottom right corner of a panel show the number of times the phenotype depicted was seen, out of the total number of embryos from that genotype analysed. A, anterior; P, posterior; L, left side; R; right side. Scale bars: 50 μm.


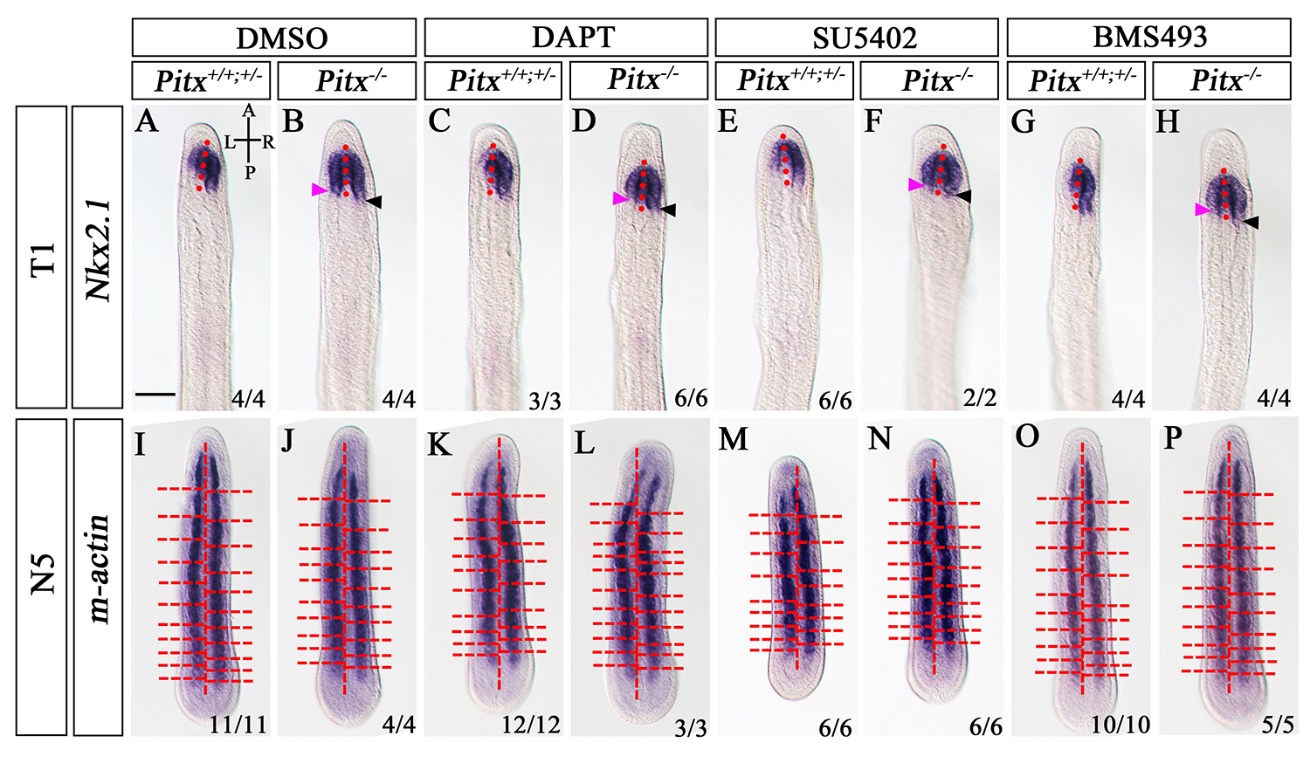


**Fig. S17** The factor X factor is not NOTCH signaling, FGF signaling or RA signaling. (a-h) In DMSO treated groups, *Nkx2.1* expression is in a L<R asymmetrical manner (a, c, e, g). *Nkx2.1* expression is still incomplete symmetrical in *Pitx* knocking out mutants in embryos treated with DAPT (d), SU5402 (f), or BMS493 (h) from N0 to T1 stage. Red and black arrowheads indicate the posterior expression border of *Nkx2.1* in endostyle on the left and right side, respectively. (i-p) The treatments from N0 to N5, do not affect the arrangement of somites marked by m-actin (k-p), compared with the DMSO-treated embryos (i, j). Longitudinal dashed lines mark notochord, transversal dashed lines mark somite outlines. All images are dorsal views. Numbers in the bottom right corner of a panel show the number of times the phenotype depicted was seen, out of the number of embryos of that genotype analyzed. A, anterior; P, posterior; L, left side; R; right side. Scale bars: 50 μm.
