# Supplementary material for: Pitx controls amphioxus asymmetric morphogenesis by promoting left-side development and repressing right-side formation
Source: BMC Biol. 2021 Aug 20;19:166. doi: 10.1186/s12915-021-01095-0 (PMC8377849; doi:10.1186/s12915-021-01095-0)
Supplement: Supplementary file 2 — Additional file 2: Table S1. Primers used for amplifying gene fragments including TALEN target sites and the amplicon sequences. Table S2. Primers used for making pGEM-T-Easy constructs. Table S3. Primers used for cloning and mutagenesis of Lhx3 and Hex promoter sequence. [file 12915_2021_1095_MOESM2_ESM.docx]

**Additional file 2**

***Pitx* controls amphioxus asymmetric morphogenesis by promoting left-side development and repressing right-side formation**

**This file contains:**

**1-Table S1.** Primers used for amplifying gene fragments including TALEN target sites and the amplicon sequences.

**2-Table S2.** Primers used for making pGEMT-Easy constructs.

**3-TableS3.** Primers used for cloning and mutagenesis of *Lhx3* and *Hex* promoter sequence.

**Table S1:** Primers used for amplifying gene fragments including TALEN target sites and the amplicon sequences.

| **Genes** | **Primers sequences (5′-3′)** | **Amplicon sizes (bp)** | **Amplicon sequences (5′-3′)** |
| --- | --- | --- | --- |
| ***Pitx* TALEN1** | Forward: ATGGACACCTTGAACGACTCGC  Reverse: CGACACGGGTAATTTGGAGC | 480 | ATGGACACCTTGAACGACTCGCTGAGCTTAGAGCAGCTTGTGAGCGTAAGCCCACGGAGAAGCCAACTGACTATGGCCGGGATGGCGCCGATCAACAACAGTGGCACAAGTACGGGCAGCGCATTGGACAGCACGGCGGTAGCGGGAGCTCACACCAGCATGGCCGGGACAGATAGCAGCATGGACTCCACTCACGGCTCCGGAGgtacagaccaactccgtaactactgtactacgtactgctactacacgccttagccggcgtgcgacggtgcccgactcctgtaatttctgtggtttgtgcgacgttaggtctgctcgcgagcttgtcgagcgtggccccgagcacgagtaaggtgccgccgaggcccggcgagccaacctgctcctggggtgtgtagttgttaggcaaaaatcctggccctgccggtaccgcgatgagagcccgtcccctaacctcgctccaaattacccgtgtcg |
| ***Pitx* TALEN2** | Forward: GGGAGTGTGTGTCGGAATGT  Reverse: AGACAACCGCACGACTTCAA | 426 | gggagtgtgtgtcggaatgtcagcctagttctagcccatgtaaaccctgctaaccgccgtcttctctctctctctctctcccccctacagCAGCCACTGTGTCGGCAGGCTCGCCCGTCGGGAAGGACTCCGGTAGCGGGTCCACGCCGTCTACGCAGGACGTCACGCAAGACGACGACGAGATGCGGAAGCGACGGCGGCAGCGTCGGCAGAGGACGCACTTCACGTCGCAGCAGCTCCAGGAGCTAGAGGCCTCCTTCGCGCGGAATCGCTACCCCGACATGGCGACACGGGAGGAGATCGCCGCCTGGACCAACCTCACAGAGGCCAGAGTTAGGgtaagtcttacattacaactccattttgtaatagctttaatacagtaaaacagagtcagctatcgtatttgaagtcgtgcggttgtct |
| ***Pitx* TALEN3** | Forward: AGGTCTGGTTCAAGAACCG  Reverse: TCACGGTAAGCGTAAGGCTG | 406 | agGTCTGGTTCAAGAACCGGCGGGCCAAGTGGCGGAAGCGCGAGCGAAATCAGCTGGGAGAATTCAAGAACGGCTTCGGTCCTCACTTCAACGGGTTGATGCAACCGTTCGACGACGGACTGTACTCCGGCTACTCGCCGGCCTACAACAACTGGGCGGCGAAGGTGCCGAGCCCGCTCACCGCCAAGTCCTTCCCGTGGGGACTCAACTCCAGCGGCGTGCCCAACGTCAACCCGCTGTCGTCTCAGGCCATGTGCTTCACCCCGCCGACCACCATCGGCACCGCCACCACCATGGTGCCGAGCATGAACGTCGGCAACGGCTTGAATTCCCTGAGCAGCCTGCAGAACCCGACGGTCGCGCCGTGCCCCTACGCCTCCCCCGGGCAGCCTTACGCTTACCGTGA |

**Table S2:** Primers used for making pGEMT-Easy constructs

| **Genes** | **Primer sequences (5′-3′)** | **GenBank** | **Amplicon sizes (bp)** |
| --- | --- | --- | --- |
| *Pitxa/b* | Forward: GTTGGGCCGACTGTCA  Reverse: CTGGCTTGATATCGTCGGTG | MW653803 | 316 |
| *Pitxc* | Forward: GCGAGTATGTCGTTCCCGTC  Reverse: CTCCGGAGCCGTGAGTGGAG | MW653804 | 385 |
| *Hex* | Forward: ATGAGTCTCCAGTACGGGACC  Reverse: CTATGAACTCTGCTTCTTGTCC | EU296398.1 | 855 |
| *Prop1* | Forward: ACGAACGGCTGGGAAACAA  Reverse: CGAAGAGAGACGCCCTATG | MW650862 | 874 |
| *Gata1/2/3* | Forward: GACGTGTTCTTCCACCACCT  Reverse: GGTACTGGAGCGTTTCGTCT | FJ615537.1 | 678 |
| *Pdvegfr* | Forward: ACTGACCAATCAGAGCTCACG  Reverse: CCCGAGTCTTCCTCTTTCTTGC | MW650861 | 773 |
| *Scl* | Forward: CGGATATGGAACCTGCGGAA  Reverse: AAGCTAAGCTCCAACCTCGC | MW650860 | 1136 |
| *Hand* | Forward: ACCATCCCAGCAGTACCAG  Reverse: CACCGACACAAGGAGTGGA | HQ605708.1 | 1072 |

**Table S3:** Primers used for cloning and mutagenesis of *Lhx3* and *Hex* promoter sequence.

| ***Lhx3*** | **Primer sequences** |
| --- | --- |
| Cloning primers | Forward: TCGAGATCTGCGATCTAAGTCTGGTGATGATCTAATTTCA  Reverse: ACCAACAGTACCGGAATGCCTTTGCTCTGCTTGTTGCAGC |
| Binding site 1 mutagenesis primers | Forward: CTAAGTCTGGTGATGATCgAgcTaCATATCAGTACATGACTT  Reverse: AAGTCATGTACTGATATGtAgcTcGATCATCACCAGACTTAG |
| Binding site 2 mutagenesis primers | Forward: CGGCAGCTTCCAGAAAGGttCgTgGTTTAGCTTTTCTTGCCC  Reverse: GGGCAAGAAAAGCTAAACcAcGaaCCTTTCTGGAAGCTGCCG |
| Binding site 3 mutagenesis primers | Forward: CGCGAGCGCACGGCTCTGggTcGTAGTCCCGGGACTTCTG  Reverse: CAGAAGTCCCGGGACTACgAccCAGAGCCGTGCGCTCGCG |
| ***Hex*** | **Primer sequences** |
| Cloning primers | Forward: TCGAGATCTGCGATCTAAGTGTTTTACGTCTGATTTTTCT  Reverse: ACCAACAGTACCGGAATGCCCTTCCTCGGTACGTAACGTT |
| Binding site 1 and 2 mutagenesis primers | Forward: TGTATGCAAGCTTAGTATGCggTcACCATAGCATACTTACGT  Reverse: ACGTAAGTATGCTATGGTgAccGCATACTAAGCTTGCATACA |
| Binding site 3 mutagenesis primers | Forward: GTGGCTATTGTTCTCGAGTGAAgcTcATTGATATT  Reverse: CACTCGAGAACAATAGCCACgAcTgACTTTACTCT |
