# Supplementary material for: Pitx controls amphioxus asymmetric morphogenesis by promoting left-side development and repressing right-side formation
Source: BMC Biol. 2021 Aug 20;19:166. doi: 10.1186/s12915-021-01095-0 (PMC8377849; doi:10.1186/s12915-021-01095-0)
Supplement: Supplementary file 3 — Additional file 3. Uncrooped gel images. Uncropped gel images shown in Additional file 1 figures. [file 12915_2021_1095_MOESM3_ESM.docx]

**Additional file 3**

**This file contains all the images of original agarose gel electrophoresis in this study.**

**
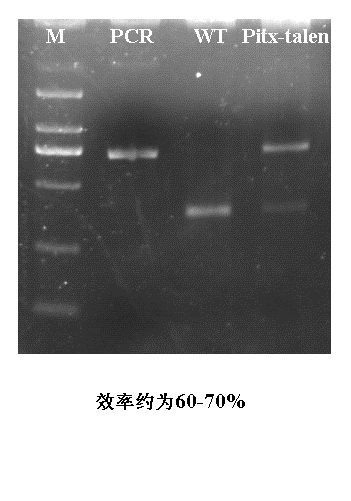
**

**Fig. S1**

**
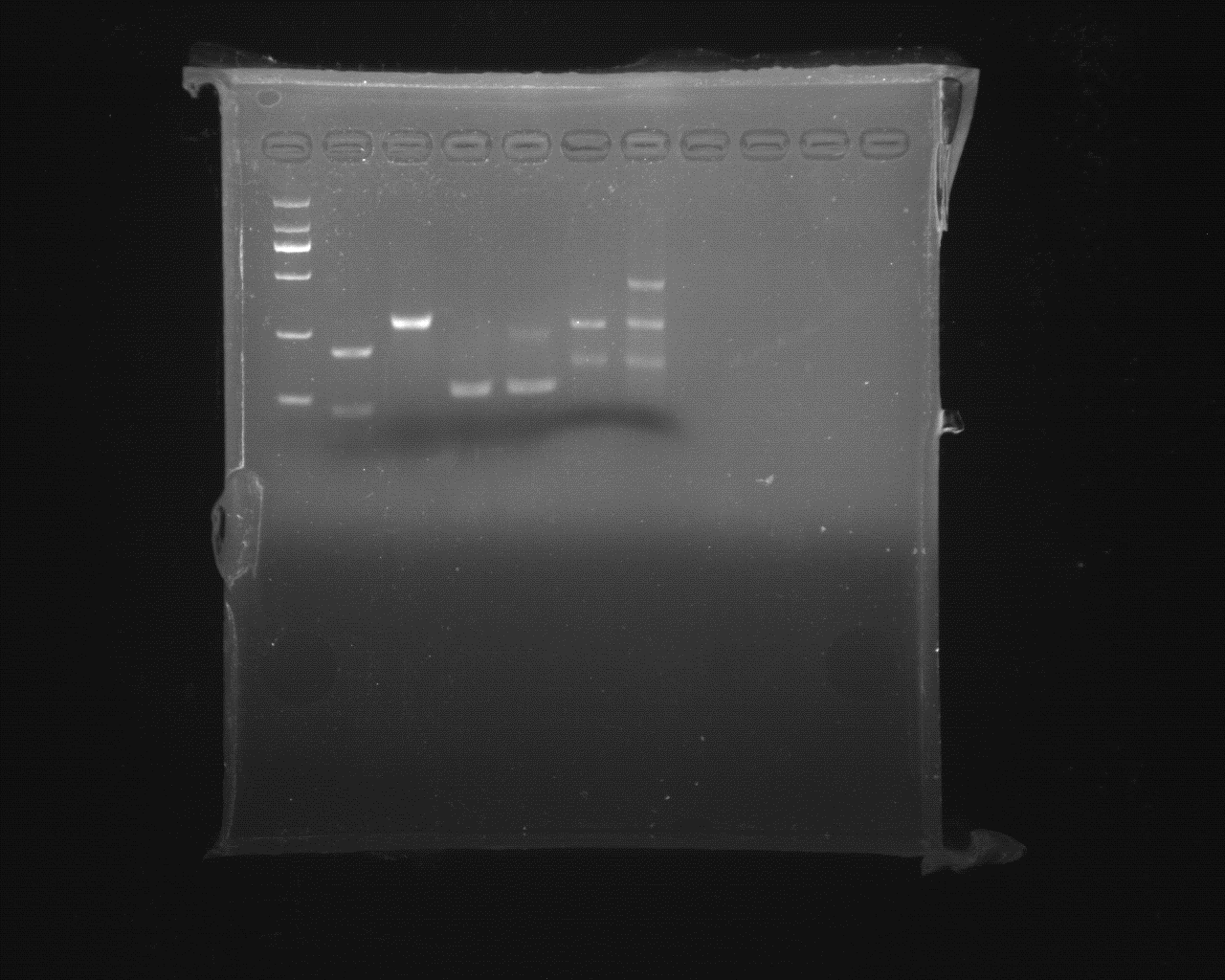
**

**Fig. S2** Original agarose gel electrophoresis image of mutation rates of *Pitx* TALEN2 in F0 embryos. Lane 1 is DNA marker, Lane 6 is PCR products from wild type digested with *Aat*II, Lane 7 is PCR products from injected embryos digested with *Aat*II. Lanes 2-5 are bands irrelevant to this study.

**
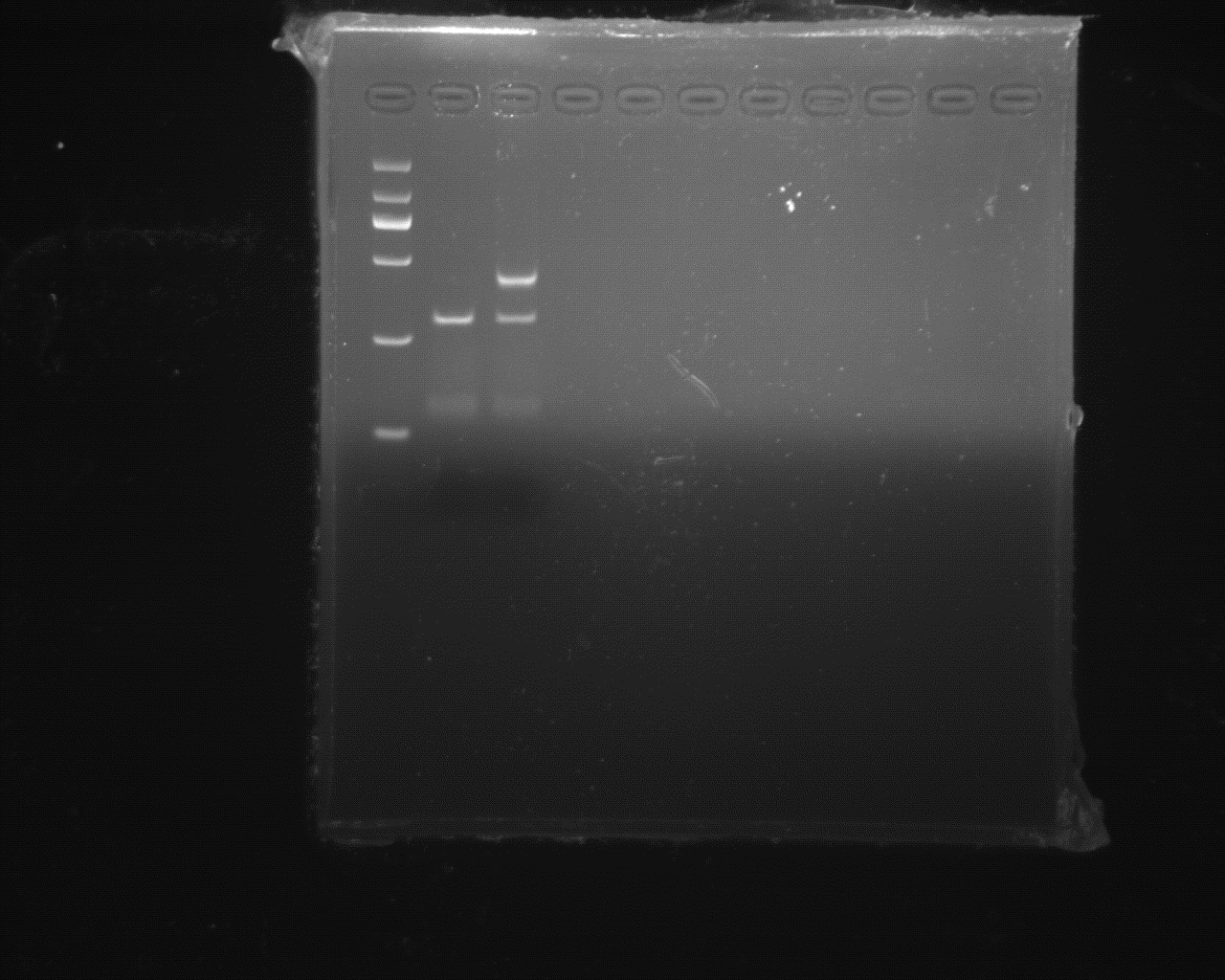
**

**Fig. S3** Original agarose gel electrophoresis image of mutation rates of *Pitx* TALEN3 in F0 embryos. Lane 1 is DNA marker, Lane 2 is PCR products from wild type digested with *Tat*I, Lane 3 is PCR products from injected embryos digested with *Tat*I.

**
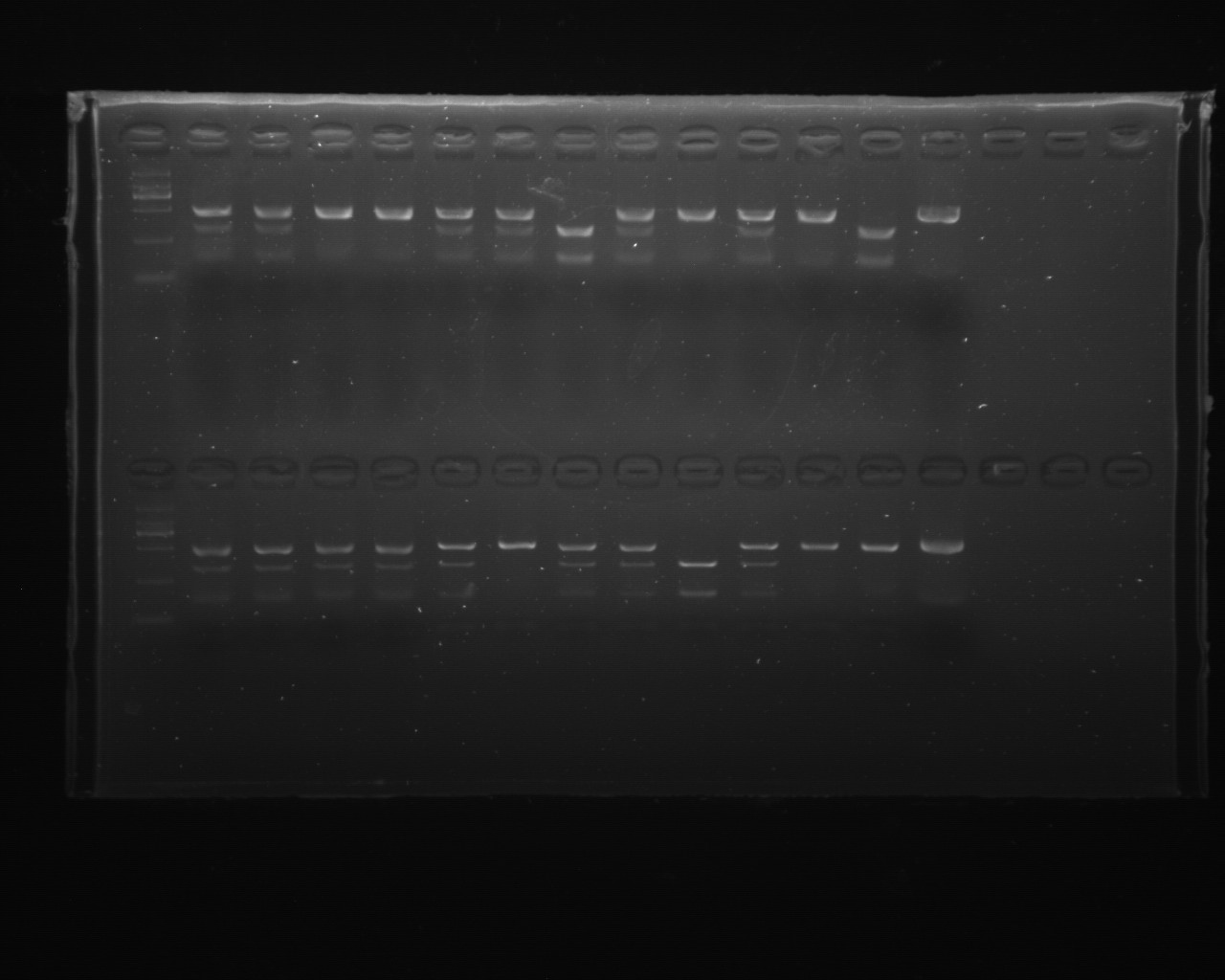
**

**Fig. S4** Original agarose gel electrophoresis image of genotype analysis of *Pitx* TALEN1 mutants. Lanes 2- 13 of the first line gel holes are PCR products containing the TALEN1 target site and digested by *Sac*I. The bands in second row of gel holes are irrelevant to this study.
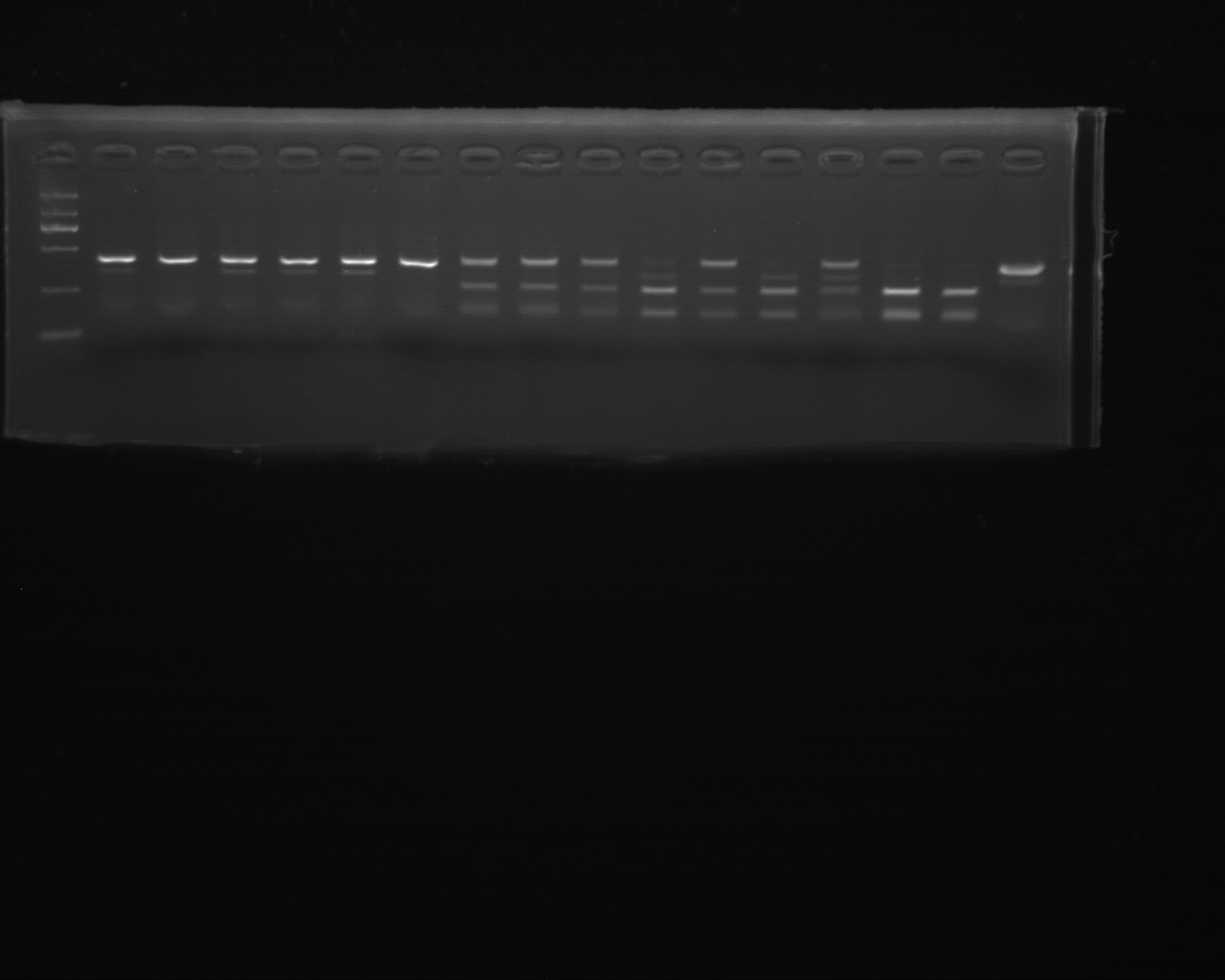


**Fig. S5** Original agarose gel electrophoresis image of genotype analysis of *Pitx* TALEN2 mutants. Lane 1 is DNA marker. Lanes 2-16 are PCR products containing the TALEN2 target site and digested by *Aat*II.

**
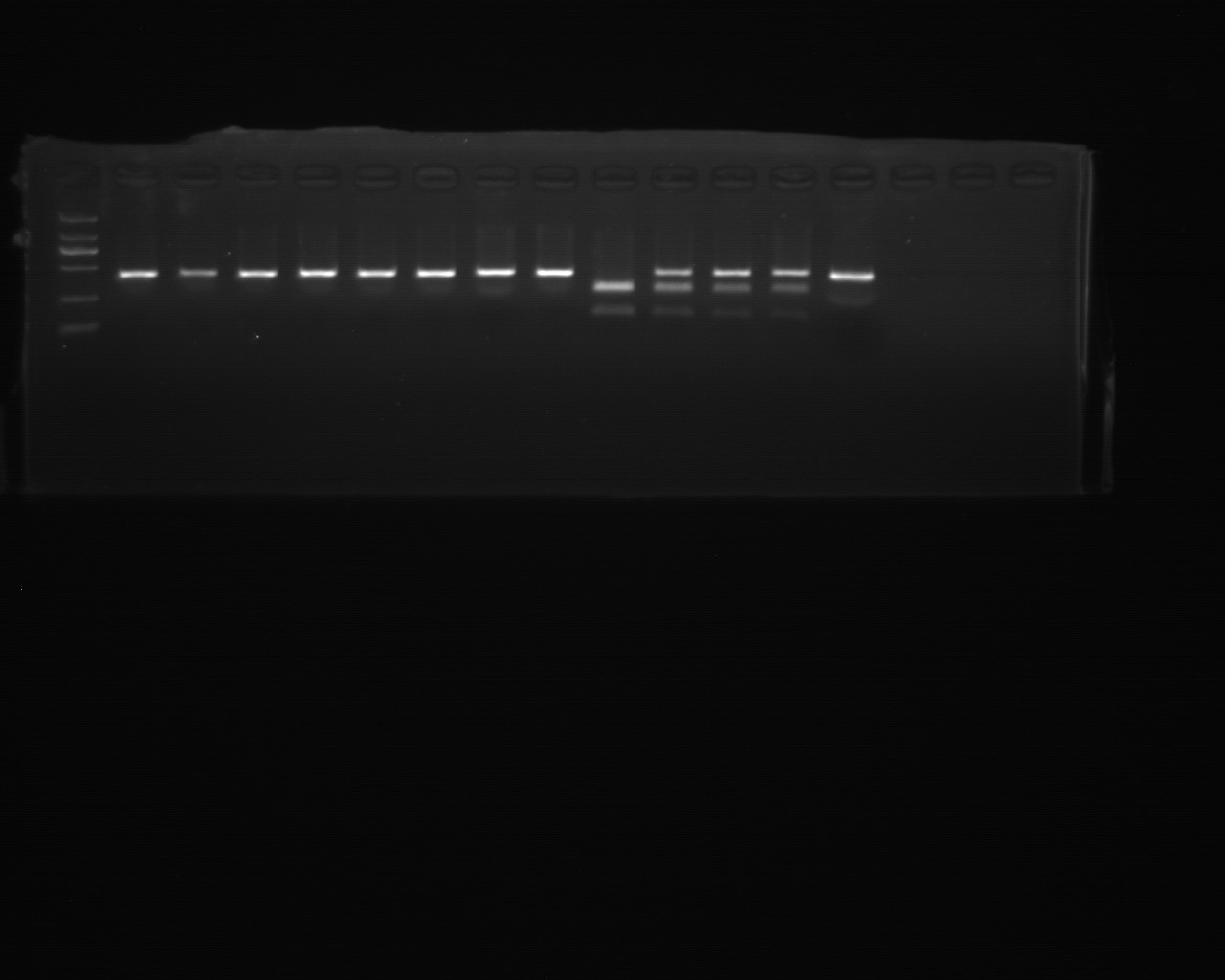
**

**Fig. S6** Original agarose gel electrophoresis image of genotype analysis of *Pitx* TALEN3 mutants. Lane 1 is DNA marker. Lanes 2-13 are PCR products containing the TALEN3 target site and digested by *Tat*I.


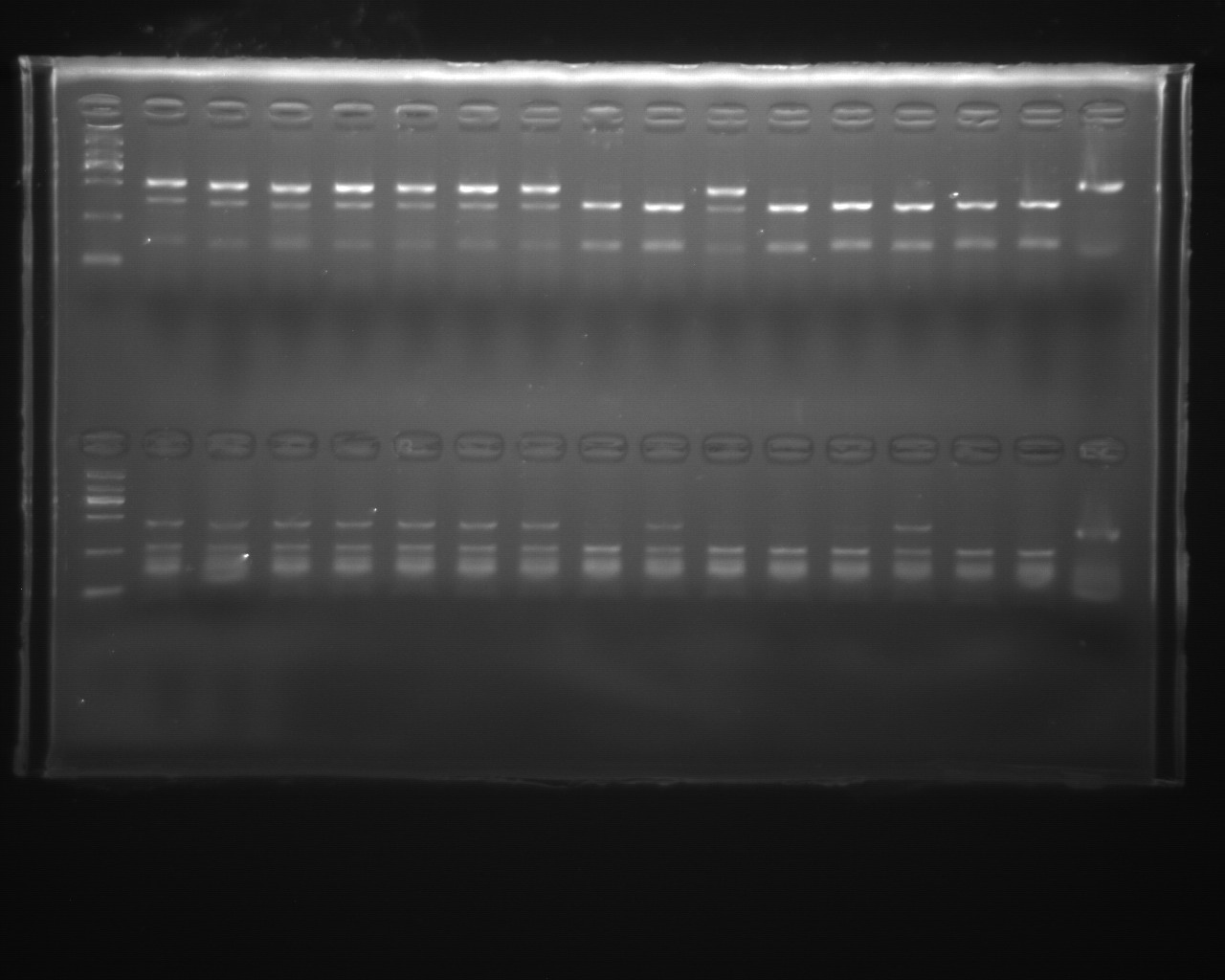


**Fig. S7** Original agarose gel electrophoresis image of genotype analysis of *Pitxa/c* homozygotes carrying mutations at TALEN1 and TALEN2. The first line gel holes are PCR products containing the TALEN1 target site and digested by *Sac*I. The second line gel holes are PCR products containing the TALEN2 target site and digested by *Aat*II.

**
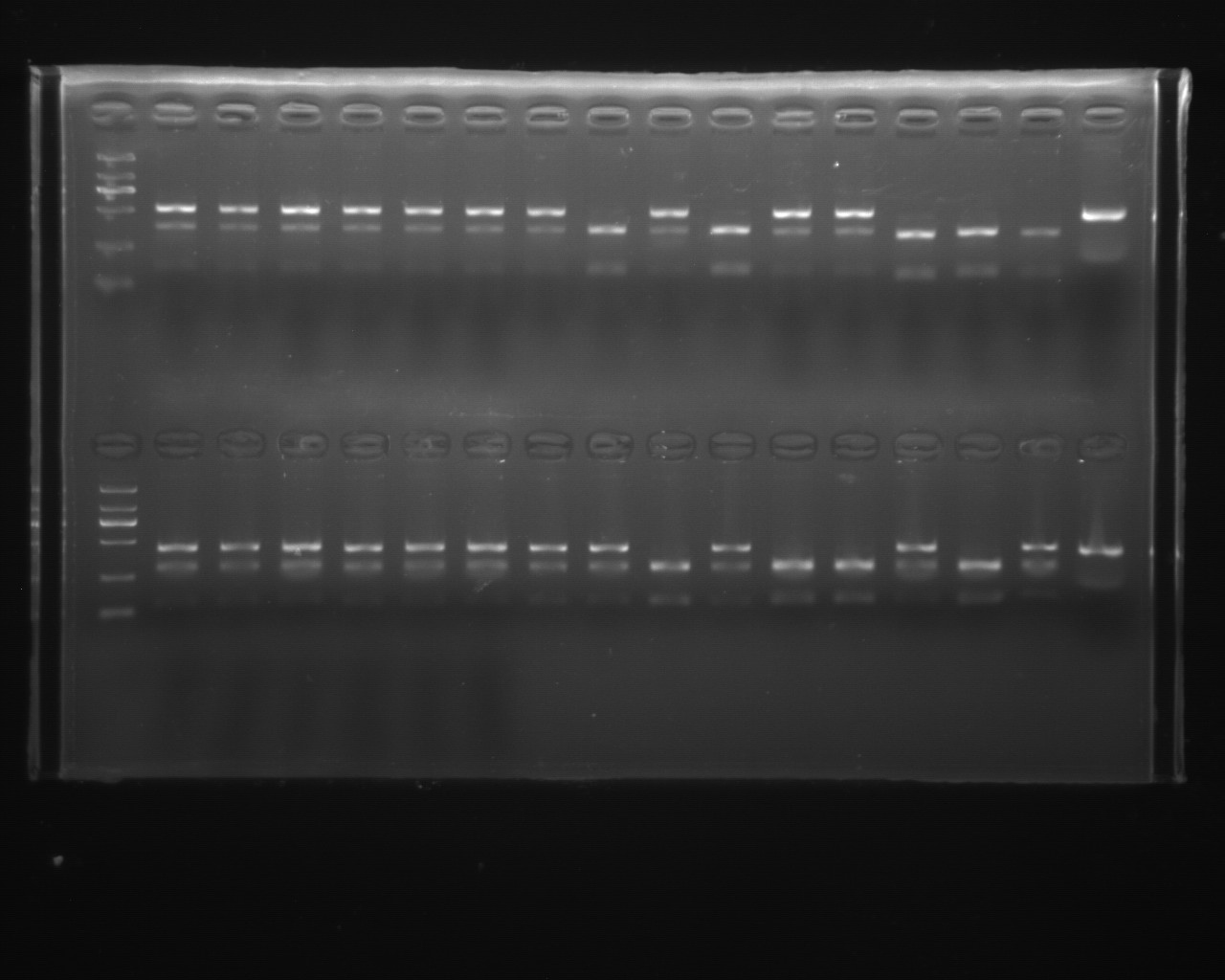
**

**Fig. S8** Original agarose gel electrophoresis image of genotype analysis of *Pitxa/c* homozygotes carrying mutations at TALEN1 and TALEN3. The first line gel holes are PCR products containing the TALEN1 target site and digested by *Sac*I. The second line gel holes are PCR products containing the TALEN3 target site and digested by *Tat*I.
